# Feasibility and Acceptability of a Novel Algorithm for Physicians to Prescribe Personalized Exercise Prescriptions to Patients with Cardiovascular Disease Risk Factors: Study Protocol for an Exploratory Randomized Controlled Crossover Trial

**DOI:** 10.3390/healthcare14020188

**Published:** 2026-01-12

**Authors:** Alexander J. Wright, Gregory A. Panza, Antonio B. Fernandez, Peter F. Robinson, Victoria R. DeScenza, Ming-Hui Chen, Elaine C. Lee, Margaux A. Guidry, Linda S. Pescatello

**Affiliations:** 1 Department of Kinesiology, University of Connecticut, Storrs, CT 06269, USA; tori.descenza@uconn.edu (V.R.D.); elaine.c.lee@uconn.edu (E.C.L.); linda.pescatello@uconn.edu (L.S.P.); 2 Hartford HealthCare Research Administration, Hartford Hospital, Hartford, CT 06106, USA; gregory.panza@hhchealth.org; 3 Hartford HealthCare Heart & Vascular Institute, Hartford Hospital, Hartford, CT 06106, USA; antonio.fernandez@hhchealth.org; 4 Pat and Jim Calhoun Cardiology Center, UConn Health, Farmington, CT 06030, USA; perobinson@uchc.edu; 5 Department of Statistics, University of Connecticut, Storrs, CT 06269, USA; ming-hui.chen@uconn.edu; 6 SalesStar, Charlotte, NC 28078, USA; maggie.zwierko@salesstar.com

**Keywords:** chronic, diabetes, dyslipidemia, exercise therapy, health behavior, hypertension, obesity, precision medicine, primary health care

## Abstract

**Background:** Approximately half of U.S. adults have ≥1 cardiovascular disease (CVD) risk factors. Exercise is universally recommended as a first-line lifestyle therapy to prevent and treat CVD. **Objective:** We will conduct a feasibility and pilot efficacy randomized controlled trial to test the usability and user satisfaction of an evidence-based digital health tool we developed for physicians—the *P*rioritizes *P*ersonalizes *P*rescribes *EX*ercise algorithm (P3-EX)—to treat patients with CVD risk factors (ClinicalTrials.gov: NCT07238556). **Methods:** We will recruit 24 physicians who do not prescribe written exercise prescriptions (ExRx) from two local CT hospitals. Physicians will recruit two patients each (N = 48); both patients must have CVD risk factors. Each physician will deliver a P3-EX ExRx to one patient (n = 24) and the Physical Activity Vital Sign ExRx to the other patient (n = 24) in a random sequence crossover design. Physicians and patients will rate the feasibility and acceptability of each ExRx method using validated questionnaires. Patients will perform their ExRx for 12 weeks and complete an exercise diary to monitor exercise adherence with weekly virtual oversight by Research Assistants. Before and after the exercise intervention, we will measure patient CVD risk factors and physical activity levels via accelerometry. **Results:** This trial has received Institutional Review Board approval (E-HHC-2025-0198) and will begin in January 2026, with findings published in 2027. **Conclusions:** This protocol provides the scientific rationale and methodology to test P3-EX within a real-world clinical setting, to inform the feasibility of using P3-EX as a digital health support tool by physicians, and preliminary efficacy of P3-EX to improve patient cardiovascular health and physical activity levels.

## 1. Introduction

### 1.1. Background

Cardiovascular disease (CVD) is the most prevalent and costliest chronic disease worldwide, accounting for one in four deaths per year in the U.S. [1]. The direct healthcare costs associated with CVD are projected to rise from USD 393 billion in 2020 to USD 1490 billion by 2050 [2]. Approximately half of U.S. adults have one or more of the major CVD risk factors of obesity, hypertension, dyslipidemia, and diabetes [1]. Due to the numerous health benefits of physical activity (PA) [3], professional organizations such as the American College of Sports Medicine (ACSM) and American Heart Association (AHA) recommend exercise as a first-line lifestyle therapy to prevent and treat CVD and its risk factors [4,5]. However, only about 25% of U.S. adults meet the PA recommendations of 150 min per week of moderate and/or 75 min per week of vigorous intensity aerobic exercise or a combination of both and 2 days per week of resistance exercise [6].

To address these significant public health challenges, there is an urgent call for clinicians to recommend PA to their patients [7]. Although a standard of care for PA recommendation is not established in medical practice, the ACSM advocates for clinicians to assess and prescribe PA as a vital sign (PAVS) in routine medical visits with every patient [8,9]. Indeed, a physician’s recommendation to exercise provides their patients with a strong incentive to be physically active [10,11]. However, only about 20% of U.S. adults and 44–56% of patients with CVD risk factors report being advised by their healthcare providers to exercise [12,13]. When they are advised, 80% of them are recommended to walk [14]. Although physicians are receptive to prescribing exercise and counseling their patients to exercise [15,16], they encounter barriers to do so such as a lack of time, training, confidence, and tools [17,18].

Exercise prescription (ExRx) is the process of structuring an individualized PA program by the *F*requency (How Often?), *I*ntensity (How Hard?), *T*ime (How Long?), and *T*ype (What Kind?) principle of exercise or FITT [19]. Personalized ExRxs are preferred to generic approaches because (1) they optimize effectiveness and minimize adverse effects [20,21]; (2) consider patient preferences and goals [22]; (3) align with patient-centered care [23]; and (4) inform shared decision making [24]. For these reasons, the ACSM recommends individualized FITT ExRxs for 26 different chronic diseases and health conditions that include the major CVD risk factors of obesity, hypertension, dyslipidemia, and diabetes [25,26,27,28].

The use of digital health tools has grown significantly among physicians due to their increased efficiency for providing clinical decision support and remote care for their patients [29]. Digital health tools show promise as clinical decision support systems to guide physicians in prescribing exercise to their patients with chronic diseases and cardiovascular disease risk factors [30,31]. L.S.P., assisted by G.A.P., has developed a clinical decision support tool that *P*rioritizes *P*ersonalizes *P*rescribes *EX*ercise (P3-EX) [32]. P3-EX includes (1) the ACSM exercise preparticipation health screening recommendations to determine if there is a need for medical clearance [33]; (2) an adapted AHA Life’s Essential 8 [34] cardiovascular health scoring system to determine the CVD risk factor posing the greatest risk [22]; and (3) the ACSM strategies for designing an ExRx for people with multiple CVD risk factors [25] to produce a prioritized FITT ExRx framed for the CVD risk factor posing the greatest risk. P3-EX is evidence-based, being founded on the ACSM and AHA ExRx standards, is time efficient, and enables clinicians with no ExRx training to produce personalized ExRx for their patients, to optimize their cardiovascular health [32]. The novelty of the P3-EX algorithm meets an unmet clinical need and unique market niche [35].

### 1.2. Objectives

What remains to be carried out is a feasibility and pilot efficacy randomized control trial (RCT) to test the usability and user satisfaction of P3-EX in a real-world clinical setting. Our primary aim is to evaluate the feasibility and acceptability of P3-EX for physicians to use to prescribe exercise to patients with CVD risk factors. Our secondary aim is to explore the preliminary efficacy of P3-EX to improve the patient CVD risk factors of blood pressure, body composition, blood lipid-lipoproteins and glucose, objective and subjective PA levels, and exercise adherence. We hypothesize P3-EX will be feasible for physicians to use to prescribe customized exercise routines for patients with CVD risk factors, and they and their patients will be satisfied with P3-EX. We also hypothesize that P3-EX will result in favorable improvement trends in patient CVD risk factors, PA levels, and exercise adherence.

## 2. Materials and Methods

This study is named the *P3-EX Feasibility Trial* and is reported accordingly with Standard Protocol Items from the Recommendations for Interventional Trials 2025 checklist [36], available as Supplementary File S1. This trial was registered on ClinicalTrial.gov (NCT07238556; https://clinicaltrials.gov/study/NCT07238556) on 19 November 2025. The trial will be managed by Co-Principal Investigators A.B.F., L.S.P., and P.F.R.; Co-Investigators G.A.P., V.R.D., M.-H.C., and E.C.L.; a Student Investigator and Graduate Research Assistant A.J.W.; and University of Connecticut (UConn) Research Assistants (RAs). Their planned roles are specified throughout the Materials and Methods (Section 2) and listed under Author Contributions.

### 2.1. Trial Design

**Figure 1:**
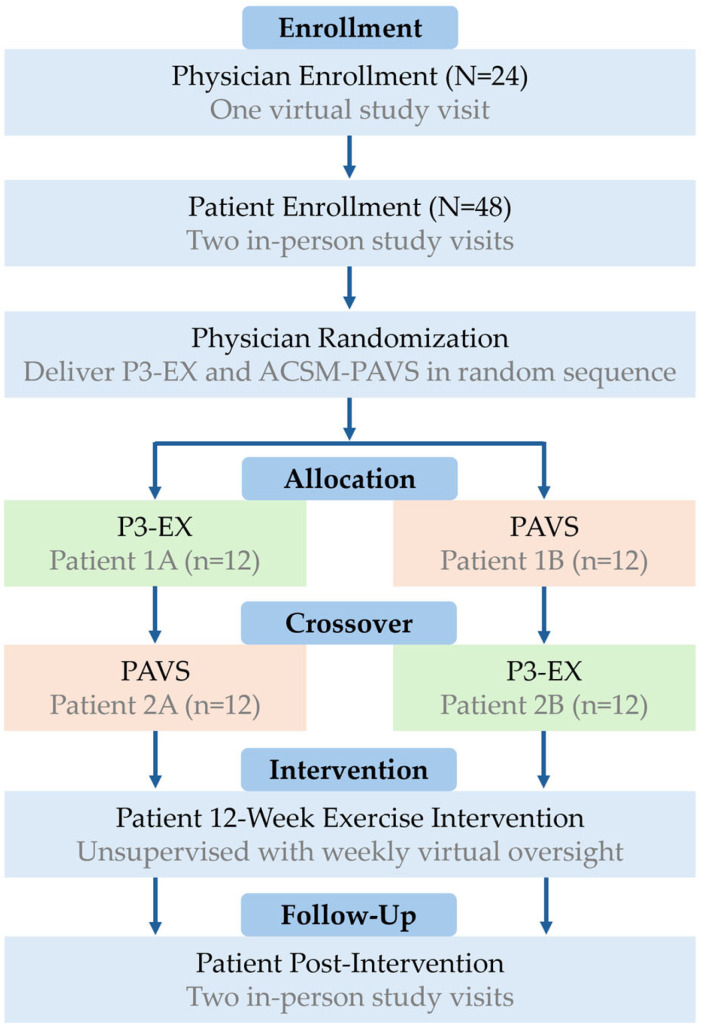
Overview of the study design. P3-EX: *P*rioritizes *P*ersonalizes *P*rescribes *EX*ercise, PAVS: Physical Activity Vital Sign.

The P3-EX Feasibility Trial is a feasibility and pilot efficacy RCT with a two-arm crossover exploratory design (Figure 1). Physicians (N = 24) will be individually randomized with a 1:1 allocation ratio to deliver P3-EX to one of their patients (n = 24) and PAVS to another patient (n = 24) in random sequence. Because physicians will deliver P3-EX and PAVS to patients during their routine healthcare appointments that are scheduled in advance of their enrollment, we will not implement a washout period for physicians between their delivery of P3-EX and PAVS, but will control for potential period or carryover effects in statistical analyses.

### 2.2. Trial Setting

Physicians and patients will be recruited from primary care, outpatient community, and hospital settings in CT, U.S. A.J.W. will conduct enrollment and data collection at two local hospital clinics in Hartford and Farmington, CT, U.S. [37,38]. Physicians will deliver P3-EX and PAVS at the medical setting where they practice, and patients will perform an unsupervised exercise intervention at a setting of their preference. There is no planned patient or public involvement to inform the design, conduct, or reporting of the trial.

### 2.3. Eligibility Criteria

The inclusion and exclusion criteria for physicians and patients are listed in Table 1. We will recruit primary care physicians and preventive care specialists, and their patients who will be physically inactive healthy adults with ≥1 CVD risk factor. Patients will agree to maintain their medication routine and habitual diet and not follow other exercise or nutrition programs.

### 2.4. Physician and Patient Recruitment and Enrollment

A.B.F., G.A.P., and P.F.R. will email their physician colleagues, and physicians will be notified using listservs and newsletters distributed by Hartford HealthCare, UConn Storrs, and UConn Health. Flyers and leaflets will be used at the hospital clinics and community locations. Physicians will complete an online screener and attend a one-on-one virtual study orientation visit led by A.J.W. and an RA over Microsoft Teams to provide consent; confirm eligibility; provide demographics including age, gender, ethnicity, medical specialty, the number of years in practice, prior experience using digital health tools; assess barriers to and confidence with ExRx and PA levels; be orientated to the study procedures; and receive brief ExRx delivery training regarding their patients. A.J.W. will use a standardized script from our laboratory to verbally and uniformly train physicians on the study procedures to deliver the P3-EX and PAVS ExRxs, detailed in their ExRx Instruction Manual (Supplementary File S2), and physicians will not practice using P3-EX or PAVS prior to using each ExRx method.

The enrolled physicians will identify patients who have upcoming appointments with them through the hospitals’ scheduling or electronic medical record systems. Physicians will briefly screen health history profiles of their upcoming patients using the study inclusion criteria. Physicians will inform patient candidates by uploading an electronic flyer to their MyChart account and by phone call. Patients will complete a phone screening and attend two one-on-one in-person study visits led by A.J.W. and an RA to provide consent; confirm eligibility; provide demographics including age, sex, gender, ethnicity, education level, household composition, salary, and technology use; and undergo pre-intervention assessments of PA levels and CVD risk factors.

### 2.5. Physician Randomization

#### 2.5.1. Sequence Generation

We will use block randomization [43] to assign the ExRx delivery sequence among physicians (N = 24), stratified by those who meet the PA guidelines [44] (n = 12) and those who do not meet them (n = 12). Within each PA level, physicians will be randomly assigned to one of two delivery sequences using permuted blocks of four: (1) P3-EX followed by PAVS, or (2) PAVS followed by P3-EX. This approach aims to ensure equal allocation of the ExRx delivery sequences across PA levels to control for the potential confounding effect of physician PA levels on the primary aim of the study [45].

#### 2.5.2. Allocation Concealment and Implementation

V.R.D., who is not involved in data collection or outcome assessment to maintain allocation concealment, will create a computer-generated randomization schedule (https://www.sealedenvelope.com/, 30 November 2025) [46] maintained on a university-owned desktop computer with password protection. V.R.D. will prepare sequentially numbered, sealed envelopes in advance, and will only open them after physicians and patients are enrolled. V.R.D. will inform physicians of their random assignment via email after their first patient is enrolled.

#### 2.5.3. Blinding

A.J.W., who will conduct physician and patient enrollment and pre- and post-intervention outcome assessments, will not have access to the random allocation sequence. A.J.W. will be blinded to group assignments during patient pre-intervention outcome assessments, and laboratory technicians will be blinded when assessing all blood lipid-lipoprotein and glucose values. Patients will disclose details of their healthcare appointment to A.J.W. after completing all pre-intervention outcome assessments.

### 2.6. Interventions and Comparators

Each patient will attend their scheduled healthcare appointment with their physician and receive their healthcare as planned and permitted. During each appointment, in random sequence, the physician will deliver P3-EX to one patient and PAVS to the other patient.

#### 2.6.1. Delivery of the *P*rioritizes *P*ersonalizes *P*rescribes *EX*ercise (P3-EX) ExRx

**Figure 2:**
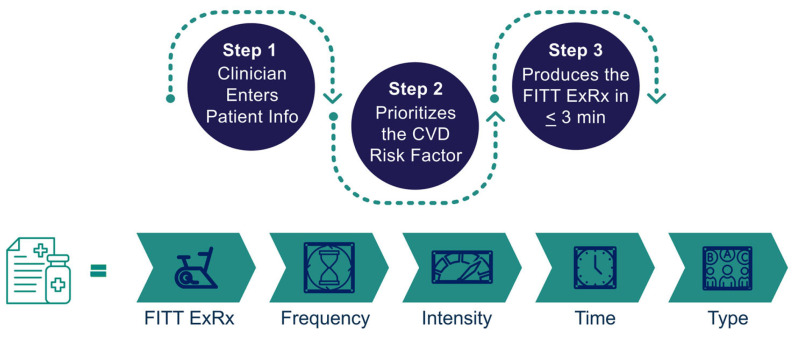
The steps of the *P*rioritizes *P*ersonalizes *P*rescribes *EX*ercise (P3-EX) algorithm. CVD: cardiovascular disease, ExRx: exercise prescription.

Physicians will use P3-EX (version 1.0.0, P3-EX LLC, Wethersfield, CT, USA) hosted on a web-based platform to deliver a personalized ExRx for improving cardiovascular health [32]. Figure 2 provides a workflow of the P3-EX algorithm.

In Step 1, the physician will enter the patient’s PA levels, presence of signs/symptoms of or having cardiovascular, metabolic, or renal disease, and desired exercise intensity. P3-EX will determine the need for medical clearance [33]. The physician will then enter the patient’s CVD risk factor values related to obesity, hypertension, dyslipidemia, and diabetes. In Step 2, P3-EX will score the patient’s CVD risk factors using an adapted AHA Life’s Essential 8 cardiovascular health scoring system to determine the CVD risk factor posing the greatest risk [22]. If ≥2 CVD risk factors are tied for the greatest risk, P3-EX will prompt the physician to choose an ACSM strategy, using their clinical judgment, to prioritize 1 CVD risk factor to personalize the FITT ExRx that is either (A) the most limiting, (B) the most conservative, or (C) encompasses the FITT of other CVD risk factors [22]. In Step 3, P3-EX will produce a personalized FITT ExRx and special exercise considerations for the prioritized CVD risk factor [25]. The physician will print and give the ExRx to the patient.

#### 2.6.2. Delivery of the Physical Activity Vital Sign (PAVS) ExRx

Physicians will use a hard copy instruction manual adapted from the Exercise is Medicine HealthCare Providers’ Action Guide [47] to deliver a generic ExRx for improving general health [48]. In Step 1, the physician will ask exercise preparticipation health screening questions to the patient to determine the need for medical clearance [33]. In Step 2, the physician will assess their patient’s PA levels as a vital sign (i.e., minutes per week of moderate to vigorous intensity exercise and days per week of resistance exercise) [8]. In Step 3, the physician will give the patient a PAVS handout [48], which recommends the PA guidelines for Americans of 150 min per week of moderate and/or 75 min per week of vigorous intensity aerobic exercise or a combination of both and 2 days per week of muscular strengthening exercise [44].

#### 2.6.3. 12-Week Exercise Intervention

A.J.W., an ACSM-certified exercise professional, with assistance from RAs, will provide patients with virtual weekly oversight of their unsupervised ExRx for 12 weeks, performed at a location the patients prefer. During the first week, A.J.W. will email all patients to provide a 12-week ExRx information packet (Supplementary File S3) containing standardized and progressive FITT exercise recommendations for the ExRx they received [25], and patients will attend a one-on-one virtual study visit led by A.J.W. over Microsoft Teams to receive standardized exercise guidance. A.J.W. will use a script to walk patients through exercise definitions and examples and how to monitor exercise intensity and progress the FITT of exercise. Throughout the intervention, A.J.W. will email all patients weekly to provide their ExRx FITT exercise goals for the upcoming week. Patients will use a validated exercise diary called the *Timeline Followback for Exercise* (TLFB-E) [49] to record the FITT of exercise they perform daily for the 12-week exercise intervention. A.J.W., with assistance from RAs, will monitor the TLFB-E for each patient to assess their adherence to the intervention and challenges they may be having. A.J.W. will email all patients with weekly feedback by providing their TLFB-E summary report from the previous week. After completing 6 weeks of the intervention, patients will attend a second one-on-one virtual study visit led by A.J.W., using a script, to inform patients how they are faring following their ExRx by reviewing their TLFB-Es and strategies to improve exercise adherence.

### 2.7. Primary Outcome Assessment

#### Feasibility and Acceptability of P3-EX

The mHealth Application Usability Questionnaire, developed by Zhou et al. (2019) [50], is validated to measure the usability of mobile health apps for healthcare providers and/or patients on 21 items separated into three subscales: Ease of Use and Satisfaction, System Information Arrangement, and Usefulness. The three subscales and the overall scale demonstrate high internal consistency and strong correlation with other validated usability questionnaires [51,52]. Physicians and patients will complete the interactive versions of the questionnaire [50], including open-ended written questions, within 48 h following the use of P3-EX and PAVS, administered by A.J.W. via email. To appropriately rate P3-EX and PAVS, we adapted question 13 to remove language that is not applicable (e.g., “responding to reminders”), and for patients to rate their ExRx, we adapted questions 3 and 13 to replace the word “interface” with “look” and “format”, respectively. The rating of each item is scaled from 1 to 7 (strongly disagree to strongly agree), and responses are averaged to provide a single score, with 7 reflecting the highest usability and 0 reflecting the lowest [50]. P3-EX will be interpreted as feasible and acceptable for physicians to use if overall scores exceed the middle score of 4.0 on the questionnaire scale [50].

The System Usability Scale [51] is the most commonly used and validated questionnaire to assess the usability of mHealth apps [53], demonstrating acceptable reliability across numerous usability studies [52]. Physicians and patients will complete the original English version of the questionnaire developed by Brooke (1986) [51] within 48 h following the use of P3-EX and PAVS, administered by A.J.W. via email. Ratings for each of the 10-items range from 1 to 5 (strongly disagree to strongly agree), and the unique score contribution for each item is summed to yield a single score ranging from 0 to 100, with 100 reflecting the highest usability and 0 reflecting the lowest [51]. P3-EX will be interpreted as having above average usability if the overall score exceeds the normative reference score of 68 [54].

### 2.8. Secondary Outcomes Assessments

#### 2.8.1. Physician ExRx Barriers to and Confidence with ExRx

Physicians will complete an adapted ExRx self-reflection questionnaire developed by O’Brien & Fowles (2017) [55] before completing the brief ExRx training and within 48 h following the delivery of either P3-EX or PAVS, to assess the impact of the trial on their perceived barriers to and confidence with ExRx. O’Brien & Fowles (2017) developed the questionnaire from a national survey and educations programs, given there are no previously validated questionnaires assessing ExRx barriers and confidence [55]. We adapted one question to be applicable for the study (i.e., ability to conduct PA screening instead of PA referral). Responses will be rated on a Likert scale from 1 to 4, with 4 indicating the highest impact from a barrier or the highest confidence with a skill and 1 indicating the lowest impact or confidence, and responses will be averaged to provide overall scores for ExRx barriers and confidence.

#### 2.8.2. AHA Life’s Essential 8 Cardiovascular Health Score

Patients will complete the validated AHA Life’s Essential 8 questionnaire [56] developed by Lloyd-Jones et al. (2022) to predict cardiovascular health [34], administered by A.J.W., to assess the metrics of diet, PA, nicotine exposure, and sleep health via survey questions, and assessments of body mass index, blood pressure, blood lipid-lipoproteins, and blood glucose at pre- and 12 weeks post-intervention. Cardiovascular health scores ranging from 0 to 100 points will be calculated using the AHA Life’s Essential 8 criteria for each metric, to calculate an unweighted average of metrics and provide a single score [34]. Higher scores are associated with reduced risk of all-cause and CVD mortality [57].

#### 2.8.3. Resting Blood Pressure and Heart Rate

Patients will be asked to abstain from exercise for 2 days prior to, alcohol for 1 day prior to, and caffeine on the day of the blood pressure measurement. Resting systolic and diastolic blood pressure (mm Hg) and resting heart rate (bpm) will be assessed by A.J.W. at pre- and 12 weeks post-intervention via automatic monitors (OMRON HEM-705CP, Omron Corporation, Kyoto, Japan and Polar F7, Polar Electro Oy, Kempele, Finland, respectively) [40]. After patients have sat for 10 min, A.J.W. will take three heart rate and blood pressure measurements 1 min apart and average them [58]. A.J.W. will take blood pressure measurements in the patients’ nondominant arm until 3 measurements agree within 5 mmHg [59].

#### 2.8.4. Body Mass Index and Waist Circumference

Patient waist circumference (cm), height (m), and weight (kg), and body mass index (kg/m^2^) will be assessed by A.J.W. at pre- and 12 weeks post-intervention via a Gullick tape measure (M-22CII, Country Technology, Inc., Brookfield, WI, USA) and a mechanical beam balance scale with stadiometer (Health o meter, Pelion, IL, USA), respectively [39]. Body composition assessments will be measured in duplicate and averaged.

#### 2.8.5. Blood Lipid-Lipoproteins and Glucose

Fasting laboratory assessments will be conducted by phlebotomy technicians and processed at convenient Quest Patient Service Centers. Patient total cholesterol, triglycerides, high-density lipoproteins, low-density lipoproteins (mg/dL), glycated hemoglobin (%), and fasting plasma glucose (mg/dL) will be assessed at pre- and 12 weeks post-intervention [41,42].

#### 2.8.6. Subjective PA Levels and Exercise Adherence

Patient self-reported PA levels will be assessed at pre-intervention, and weekly for the 12-week exercise intervention via the TLFB-E. The TLFB-E is a validated calendar diary method [49] for recording the FITT of exercise, whereby, frequency is the number of days per week that PA is performed; type is the modality of PA performed (e.g., walking, lifting weights); time is the duration of each session performed in minutes for each modality; and intensity will be assessed using the Borg 6–20 rating of perceived exertion scale [60]. A.J.W. and RAs will cross reference self-reported frequency, intensity, and time values on the TLFB-E with the 2024 Adult Compendium of Physical Activities [61] and accelerometer data to determine the agreement and verify atypical or unrealistic values. For each recorded PA, A.J.W. and RAs will assign a metabolic equivalent value from the PA compendium to calculate the PA volume performed (MET·min/wk).

#### 2.8.7. Objective PA Levels

Patient energy expenditure (kcal), steps per day, sedentary behavior (minutes/wk) and light, moderate, and vigorous intensity PA (minutes/wk) [49] will be measured at pre- and 12 weeks post-intervention via accelerometers (ActiGraph wGT3X-BT, ActiGraph Corp., Pensacola, FL, USA) [62]. A.J.W. and an RA will instruct patients to wear the accelerometer using an elastic strap with placement aligned on the hip/anterior iliac crest of their nondominant hand side except when swimming, bathing, showering, or sleeping. The ActiLife6 full version software (version 6.0, ActiGraph Corp., Pensacola, FL, USA) [63] will be used to initialize and download accelerometer data. A.J.W. will verify complete accelerometer data, defined as having ≥60% of wear time during waking hours on four consecutive days including two weekends and two weekdays. Waking hours will be calculated based on the reported sleep hours on the TLFB-E. If patients have incomplete data, they will be asked to re-wear the accelerometer for a second attempt.

### 2.9. Other Outcome Measures

#### 2.9.1. Fidelity of P3-EX and PAVS Implementation

Each time physicians use P3-EX and PAVS with their patients, we will assess the degree to which these ExRx methods are implemented as intended by obtaining the usage time (minutes) to prescribe the FITT ExRx to each patient. For P3-EX, we will also obtain the frequency of each selected ACSM strategy used to prioritize the CVD risk factor chosen by the physician when there are multiple CVD risk factors tied for the greatest risk.

#### 2.9.2. Physician and Patient Trial Satisfaction

Physicians and patients will complete open-ended written questions, administered by A.J.W., to obtain qualitative feedback [64] on their satisfaction with the pilot trial and inform the design of a larger trial. Within 48 h after completing the delivery of both P3-EX and PAVS, physicians will provide their thoughts on the recruitment procedures and the brief ExRx training they received. Patients will provide their thoughts on use of the TLFB-E and virtual exercise guidance study visits after they complete the intervention. A.J.W. will work with RAs to independently and manually code responses and identify common themes for each question, and resolve any discrepancies with V.R.D. and L.S.P.

#### 2.9.3. Harms

Patients will be asked to report any adverse events they experience during the trial to A.J.W., who will document internal or external adverse events using the Institutional Review Board (IRB) Event Accumulation Tracking Log. A.J.W. will notify A.B.F., V.R.D., and P.F.R., who will be blinded to group assignments. A.B.F., V.R.D., and P.F.R. will grade the severity of patient adverse events according to their respective institution criteria, assess the need for disclosure to the physician, and report any serious events to the IRB within 7 calendar days. All other adverse events will be submitted at the time of continuing the IRB review.

### 2.10. Physician and Patient Trial Timeline

**Figure 3:**
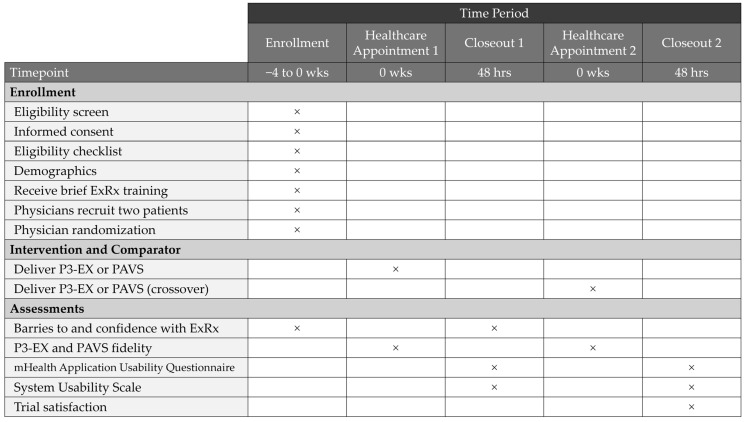
A time schedule for physician enrollment, intervention delivery, and assessments throughout the trial. P3-EX: *P*rioritizes *P*ersonalizes *P*rescribes *EX*ercise, PAVS: Physical Activity Vital Sign, ExRx: Exercise Prescription. Crosses indicate study activities occurring at discrete time points.

**Figure 4:**
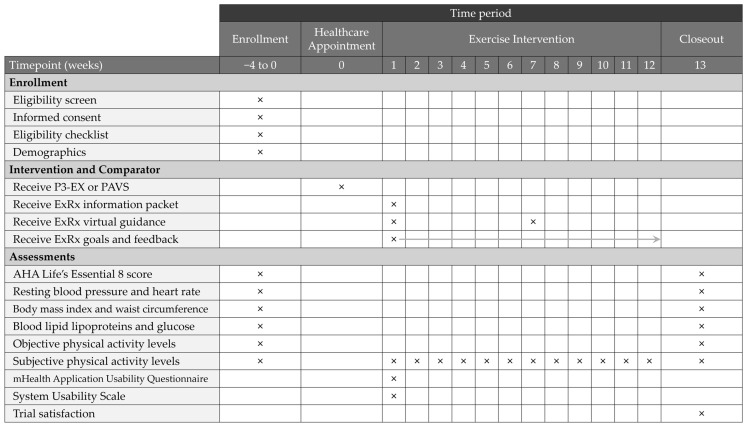
A time schedule for patient enrollment, intervention delivery, and assessments throughout the trial. P3-EX: *P*rioritizes *P*ersonalizes *P*rescribes *EX*ercise, PAVS: Physical Activity Vital Sign, ExRx: Exercise Prescription, AHA: American Heart Association. Crosses indicate study activities occurring at discrete time points. The arrow indicates continuous weekly delivery of the intervention component.

Schematic diagrams outlining the physician and patient schedule of enrollment, intervention delivery, and outcome assessments that were previously described are outlined in Figure 3 and Figure 4, respectively [36].

### 2.11. Data Collection Methods

Physicians and patients will record all survey responses and exercise diary recordings electronically in Research Electronic Data Capture (REDCap) forms, a secure web application contracted by the primary hospital institution responsible for the trial. A.J.W. will assess all vitals, body composition, and objective and subjective PA level outcomes using standardized operating procedures and record all measurements in REDCap case report forms. A.J.W. will follow standardized laboratory measurement protocols to assess each outcome [59,65,66], and provide patients with privacy during in-person study visits to complete their survey responses. RAs, trained by A.J.W., will assist A.J.W. with the assessment of exercise adherence and coding of open-ended survey questions. A.J.W., with assistance from RAs, will pilot test all instruments and REDCap surveys and forms before the start of the trial.

A.J.W. will send reminder emails to physicians and patients for scheduled study visits, and up to three reminder emails to encourage rescheduling or survey completion if there is noncompliance. We will discontinue data collection from physicians and patients who withdraw from the study but will retain any data obtained from them. We will continue data collection for patients regardless of their adherence to the intervention. A.J.W. will record any known reasons for withdrawal from the trial, noncompliance with study procedures, or non-adherence to the exercise intervention.

### 2.12. Data Management

Data entries in REDCap will include radio buttons, checkboxes, dropdown menus, and text entry with reference range checks to reduce data-entry errors, and in the P3-EX web-based interface, dropdown menus, toggle buttons, and text entry with guided inputs and reference range checks. A.J.W., V.R.D., and RAs will have access to de-identified data stored in REDCap, and only A.J.W. and V.R.D. will have access to participant identifiers stored in REDCap. A.J.W., V.R.D., and an RA will have access to all data entered in P3-EX via the Supabase backend [67]. The RA will maintain P3-EX data through weekly backups stored in Microsoft SharePoint and REDCap. All study data and records will be retained in Microsoft SharePoint for a minimum of 6 years after the completion of the study to comply with institutional policy.

### 2.13. Sample Size Estimation

We conducted a power analysis using G*Power (version 3.1.9.6, Heinrich Heine University Düsseldorf, Düsseldorf, Germany) [68] to obtain a conservative usability rating of P3-EX to evaluate the primary aim of the study. We obtained the lowest observable usability score of 4.650 ± 0.759 (mean ± SD) converted from our feasibility survey study pilot data [32] to compare to the null hypothesis middle score of 4.0 on the mHealth Application Usability Questionnaire [50]. Using a one-sided Wilcoxon signed-rank (one sample case) test [69] at the significance level (α) of 0.01 and calculated effect size between the observed and middle score of Cohen’s d = 0.856, we determined that 19 physicians are required to achieve 85% power. Therefore, we will use 19 physicians to achieve 85% power (α of 0.01), and recruit two patients per physician to complete 38 patients. Using an observed attrition of 20% for patients in the study [70,71,72,73], this trial will aim to recruit 24 physicians and 48 patients (24 patients per group). Because physicians’ PA levels could influence how they perceive and rate the delivery of an ExRx [45], we aim to recruit half of the physicians who meet PA guidelines (n = 12) [44] and half who do not (n = 12).

### 2.14. Statistical Analysis

The statistical analysis plan for this trial is accessible on ClinicalTrials.gov. Statistical analyses will be conducted by A.J.W. using Statistical Package for the Social Sciences (version 30, IBM Corp., Armonk, NY, USA) [74] with oversight from G.A.P., V.R.D., M.-H.C., E.C.L., and L.S.P., with G.A.P., M.-H.C., E.C.L., and L.S.P. being blinded to group assignments. We will first use descriptive statistics and graphical techniques to ensure all test assumptions are met, including the inspection for outliers, normal distributions [75], and homogeneity of variances [76]. Missing values will be addressed using model-based approaches and/or multiple imputations when appropriate to include the entire randomized sample [77]. If normality assumptions are not met for secondary outcomes, considerations will be made to transform the data to achieve a normal distribution or use alternative non-parametric approaches such as permutated tests. An alpha level of 0.05 will be used to determine statistical significance.

#### 2.14.1. Evaluation of the Primary Aim

We will use the following statistical approaches to evaluate the feasibility and acceptability of P3-EX for physicians to use to prescribe exercise to patients with CVD risk factors. We will use a one-sided Wilcoxon signed-rank (one sample case) test [69] to assess whether the physician mHealth Application Usability Questionnaire ratings of P3-EX and the PAVS are above the null hypothesis middle score of 4.0 on the Likert scale [50], and whether System Usability Scale ratings are above the average score of 68/100 [78]. We will use a linear mixed effects model adjusting for the delivery order of P3-EX and PAVS and the time between delivery as potential covariates and interactions between these two covariates to assess differences in the physician usability questionnaire scores between P3-EX and the PAVS. We will use normal linear regression to determine the strength of relationships between the three domains on the mHealth Application Usability Questionnaire and usage time of P3-EX and PAVS.

#### 2.14.2. Evaluation of the Secondary Aim

We will use the following statistical approaches to explore the preliminary efficacy of P3-EX to improve patient PA levels, CVD risk factors, and exercise adherence. A one-way Analysis of Variance will test if pre-intervention values are equal between groups, indicating if there is a need to adjust for potential covariates related to demographics, medication use, and/or pre-intervention PA level and CVD risk factor values. We will use a repeated measures two-way Analysis of Covariance using a linear mixed effects model to test patient differences in PA level and CVD risk factor changes over 12 weeks between the P3-EX and the PAVS groups, adjusting for potential covariates related to demographics, medication use, and/or pre-intervention values.

### 2.15. Trial Management and Monitoring

L.S.P. will work with an independent data safety monitoring committee within the university institution to oversee data collection, analysis, and interpretation plans (A.J.W., G.A.P., V.R.D., M.-H.C., and E.C.L.), to ensure the integrity of trial data related to P3-EX. Committee members will meet virtually with L.S.P., when appropriate, to discuss and validate protocol and procedural conduct related to data collection, and methods for statistical analysis and interpretation. There are no planned interim analyses or stopping guidelines for this trial.

A.J.W., with oversight from V.R.D., will keep records of physician and patient recruitment, enrollment, compliance with study procedures, attrition, and study completion throughout the trial, and report de-identified progress updates or issues to L.S.P. during weekly virtual meetings. If there are multiple adverse events or dropouts that are suspected of being associated with a specific study procedure or intervention, A.J.W., A.B.F., P.F.R., V.R.D., and L.S.P. will discuss and devise a course of action if deemed necessary. L.S.P. and V.R.D. will provide weekly virtual administrative oversight of RAs to ensure conduct and compliance with the study protocols.

### 2.16. Ethical Considerations

The P3-EX Feasibility Trial protocol was approved by the IRB (E-HHC-2025-0198) at the hospital institution of record on 24 December 2025, with sought reliance agreements at another hospital institution and a university institution. The P3-EX Feasibility Trial is sponsored by UConn (438 Whitney Road Extension, Storrs, CT 06269-1006; [860] 486-3619) with L.S.P. as the designated party responsible. The sponsor will not be responsible for the design, conduct, analysis, and reporting of the trial. Any protocol modifications will be decided by investigators jointly, with A.B.F., P.F.R., and L.S.P. providing final approval, reported to the IRBs at each institution and ClinicalTrials.gov.

#### 2.16.1. Consent Process

A.J.W., who will obtain consent from physicians and patients, has completed informed consent training required by the institutions and read all procedures. A.J.W. will obtain electronic informed consent in REDCap from physicians during the virtual orientation visit, and from patients during their first study visit. A.J.W. will provide a verbal study overview, and physicians and patients will read through the form and ask any questions before they decide to participate and e-sign. Physicians and patients will be informed that any data collected in the trial might be used in future studies and they may voluntarily withdraw from the study at any time without penalty or loss of benefits. Physicians and patients will receive a PDF copy of the informed consent for their records.

#### 2.16.2. Confidentiality

REDCap will collect and store physician and patient identifiers via eligibility screening and in separate forms, health information will be paired with de-identified study numbers instead of their names. A.J.W. will notify physicians of their patients’ health information, which they will enter into the P3-EX web-based platform from an encrypted de-identified PDF delivered via email. The P3-EX web-based platform will store de-identified patient health information paired with physician and patient de-identified study numbers. The Supabase [67] backend of P3-EX will store physician email addresses and encrypted passwords. REDCap and P3-EX are protected by institutional email and password login with multi-factor authentication. REDCap is compliant with the Health Insurance Portability and Accountability Act for secure data storage. Supabase is certified with System and Organization Controls 2 Type 2 and complaint with standards for secure data handling, encryption, and transit [67]. All results shared from the trial will be de-identified.

#### 2.16.3. Ancillary Care and Post-Trial Care

Patients will be compensated USD 50 after they complete 6 weeks of exercise intervention, and another USD 50 after they complete the study. Physicians will not receive compensation. Patients will receive healthcare during their healthcare appointment with their physician, and this trial is relatively low risk, therefore ancillary care during the trial or post-trial care will not be provided. The hospital institutions will not provide compensation for trial-related harm.

## 3. Results

The P3-EX Feasibility Trial protocol (version 1.4) was finalized on 24 December 2025. The P3-EX Feasibility Trial is expected to receive funding and begin in January 2026. Findings are expected to be available in 2027.

### Dissemination Plan

Each patient will receive their individual study results after they complete the study. Results of this trial will be presented by A.J.W. as a doctoral dissertation submitted to a university institution, and at regional and national conference meetings. Results of this trial will be reported at ClinicalTrials.gov and through manuscript writing by A.J.W. with review and editing from G.A.P., V.R.D., M.-H.C., and L.S.P., and they will be published as a preprint and article in an open access journal.

## 4. Discussion

### 4.1. Scientific Rationale

The novelty of P3-EX is supported by our systematic review which evaluated whether there are decision support tools on the market that utilize evidence-based ExRx standards of the ACSM and AHA to target CVD risk factors [35]. We evaluated 219 exercise apps that were rated ≥4 out of 5 overall with ≥1000 reviews, free to download, and not gender specific. Of the 219 apps, very few (0 to 4.3%) were evidence-based, had a preparticipation screening protocol, framed exercise plans by the FITT of ExRx, specified special considerations, or focused on chronic diseases or health conditions, and only 28% built CVD risk factor profiles. We concluded there are no evidence-based ExRx apps on the market like P3-EX.

The potential usability and user satisfaction of P3-EX in the healthcare setting is further supported by our feasibility survey study [32]. A total of 309 healthcare providers and allied health professionals, including 101 physicians, completed a timed case study using the P3-EX web-based algorithm, and then rated its satisfaction and usability using the Mobile Application Rating Scale [79]. Most of the respondents (93%) agreed they would recommend P3-EX to their colleagues and ~80% agreed P3-EX produced safe ExRx and were satisfied with P3-EX. Also, over 70% agreed P3-EX would make their patients healthier and could save them time, prescribing exercise in an average time of 4.6 min. These findings indicate P3-EX could be a viable solution to the challenges physicians face in prescribing exercise to patients with CVD risk factors [32].

### 4.2. Strengths

The P3-EX Feasibility Trial has several strengths. By contrasting the feasibility and acceptability of P3-EX to a generic approach of recommending exercise such as the PAVS, the findings effectively discern the usability and user satisfaction of P3-EX in routine medical care settings. The crossover design allows all physicians to gain exposure to both P3-EX and PAVS, which reduces potential interindividual variation in their ratings due to prior beliefs about exercise or their PA levels [45]. The trial also controls for the potential influences of past ExRx experience by recruiting physicians who do not provide written ExRx to patients, and controls for potential period or carryover effects between P3-EX and PAVS by randomizing the sequence of delivery and adjusting for these potential covariates in the statistical analyses. Regarding the efficacy of P3-EX, the trial draws on social ecological theory by targeting physician ExRx practices, influencing potential provider- and system-level determinants of patient PA levels and cardiovascular health [80]. We will also employ behavioral change techniques to target patient PA levels such as goal setting, feedback, and self-monitoring across both P3-EX and PAVS, which ensures a fair comparison and allows for exercise adherence to be depicted across the 12-week intervention period.

### 4.3. Limitations

We acknowledge potential limitations with the P3-EX Feasibility Trial. Physicians may experience burden of recruiting two of their patients to participate in the study. However, given that preventive care is one of the most common reasons for office-based physician visits [81], we anticipate that a high proportion of patients will be eligible. Concerning blinding, other than laboratory technicians assessing blood lipid-lipoproteins and glucose, we will not include other blinded outcome assessors. A.J.W. will have knowledge of patient group assignments when conducting patient exercise guidance virtual visits and assessing vitals, body composition, and objective and subjective PA levels at post-intervention. A.J.W. will therefore follow standardized laboratory operating procedures [59,65,66] to minimize observer bias, which include following outcome measurement protocols, providing privacy to patients during in-person survey completions, and reading standardized exercise guidance scripts. Although the TLFB-E is potentially susceptible to social desirability bias, we will cross reference the TLFB-E with the accelerometer at pre- and post-intervention. Last, concerning the study design, the trial does not include a non-exercise control group, which may hinder the ability to interpret the efficacy of P3-EX for improving patient PA levels or CVD risk factors. Nonetheless, this pilot data will inform the efficacy of P3-EX compared to generic ExRx approaches in routine medical care.

## 5. Conclusions

This protocol provides the scientific rationale and methodology to test P3-EX within a real-world clinical setting, to inform the feasibility of using P3-EX as a digital health support tool to be used by physicians to prescribe personalized FITT ExRx to their patients with CVD risk factors, and the preliminary efficacy of P3-EX to improve patient cardiovascular health and PA levels. If successful, this trial could demonstrate that P3-EX is a solution for physicians to overcome their barriers to ExRx, which includes lacking the required tools, training, time, and confidence [17,18]. We intend to use the pilot data for secondary outcomes to power a larger clinical trial to evaluate the efficacy of P3-EX for improving PA levels and CVD risk factors.

## Figures and Tables

**Table 1:** Physician and patient eligibility criteria.

Physician Inclusion Criteria
1. Practicing medical doctors employed at the study recruitment sites.
2. Do not currently recommend written exercise programs or plans to their patients, nor refer them to exercise clinics or exercise professionals.
3. Are willing to recruit two of their patients to deliver P3-EX to one patient and PAVS to the other.
**Patient Inclusion Criteria**
1. Have not performed planned, structured physical activity at moderate intensity for ≥30 min on ≥3 days per week in the last 3 months [25].
2. Adults: ≥18 and ≤64 yrs.
3. ≥1 CVD risk factors including obesity, hypertension, dyslipidemia, and/or diabetes (or prediabetes). Obesity defined as a BMI ≥ 30 kg/m^2^ or WC > 102 cm (40 in) for men and >88 cm (35 in) for women [39]. Hypertension defined as systolic BP ≥ 130 mm Hg and/or diastolic BP ≥ 80 mm Hg, or on antihypertensive medication [40]. Dyslipidemia defined as LDL-C ≥ 130 mg/dL (3.37 mmol/L), or on lipid-lowering medication [41]. Diabetes (or prediabetes) defined as FBG ≥ 100 mg/dL or HbA1c ≥ 5.7%, or on medication for diabetes [42].
4. Healthy having no signs or symptoms of or have CVD or renal disease, or other diseases or health conditions that significantly limit PA engagement.
5. Not pregnant or lactating.
6. Not a cigarette smoker or quit smoking ≥ 6 months ago.
7. Consume <2 alcoholic drinks daily.
8. Able to use a computer or phone with internet access.
9. Fluent in English.
**Patient Exclusion Criteria**
1. Have pain or discomfort in the chest, neck, jaw, or arms; dizziness or syncope; shortness of breath at rest or with mild exertion; unusual fatigue or shortness of breath with usual activities; orthopnea; ankle edema; intermittent claudication; palpitations; or known heart murmur. 2. Have CVD, cancer survivors or currently have cancer, chronic obstructive pulmonary disease, musculoskeletal injury, chronic back pain, depression, dementia, or other diseases or health conditions that are deemed to significantly limit physical activity engagement.

BMI: body mass index, BP: blood pressure, CVD: cardiovascular disease, FBG: fasting blood glucose, HbA1c: glycated hemoglobin, LDL-C: low-density lipoprotein cholesterol, P3-EX: *P*rioritizes *P*ersonalizes *P*rescribes *EX*ercise, PAVS: Physical Activity Vital Sign, WC: waist circumference.

## Data Availability

Data sharing is not yet applicable as the P3-EX Feasibility Trial is expected to begin in January 2026. De-identified participant-level datasets and statistical code will be made available by the authors upon reasonable request after data collection and analysis are completed.

## Supplementary Materials

The following supporting information can be downloaded at https://www.mdpi.com/article/10.3390/healthcare14020188/s1. Supplementary File S1 provides the SPIRIT 2025 Checklist [36]. Supplementary File S2 provides the ExRx Instruction Manual [47,48]. Supplementary File S3 provides ExRx Information Packets [25,48].

## Abbreviations

The following abbreviations are used in this manuscript:

ACSM	American College of Sports Medicine
AHA	American Heart Association
CVD	Cardiovascular Disease
ExRx	Exercise Prescription
FITT	*F*requency, *I*ntensity, *T*ime, *T*ype
IRB	Institutional Review Board
P3-EX	*P*rioritizes *P*ersonalizes *P*rescribes *EX*ercise
PA	Physical Activity
PAVS	Physical Activity Vital Sign
RA	Research Assistant
RCT	Randomized Controlled Trial
REDCap	Research Electronic Data Capture
TLFB-E	Timeline Followback for Exercise
UConn	University of Connecticut
