# Supplementary material for: Feasibility and Acceptability of a Novel Algorithm for Physicians to Prescribe Personalized Exercise Prescriptions to Patients with Cardiovascular Disease Risk Factors: Study Protocol for an Exploratory Randomized Controlled Crossover Trial
Source: Healthcare (Basel). 2026 Jan 12;14(2):188. doi: 10.3390/healthcare14020188 (PMC12840794; doi:10.3390/healthcare14020188)
Supplement: Supplementary file 1 [file healthcare-14-00188-s001.zip › Supplementary File S2 - ExRx Instruction Manual.pdf]

Exercise Prescription Instruction Manual

Study ID:

Principle Investigators:

Dr. Antonio Fernandez, MD, FACC, FAHA
Medical Director of Preventive Cardiology, Hartford Hospital

Dr. Linda Pescatello, PhD, FACSM
Board of Trustees Distinguished Professor of Kinesiology, University of Connecticut

Dr. Peter Robinson, MD
Assistant Professor of Cardiology, UConn Health

UConn Graduate Research Assistant:

Alexander Wright, MS
Email: Alexander.Wright@hhchealth.org
Phone: (860) 486-6814

This research is approved by the Hartford HealthCare Institutional Review Board, with reliance agreements at UConn Storrs and UConn Health. Institutional Review Board: E-HHC-2025-0198.

Purpose:

By using this manual, you, the physician, will be able to prescribe each of the exercise prescriptions you have been assigned in this study.

Instructions for physicians:

There are two different exercise programs that you will deliver in this study, one for each of your two patients enrolled.

- ***Prioritize Personalize Prescribe EXercise* [P3-EX]**
- ***American College of Sports Medicine Physical Activity Vital Sign* [ACSM-PAVS]**

1. Following your randomization, please circle which exercise program (**P3-EX** or **ACSM-PAVS**) you were assigned by the Co-Investigator (Dr. Victoria DeScenza) to deliver to Patient #1 and Patient #2.
2. Please write the Study ID of the patient provided by the Co-Investigator (Dr. Victoria DeScenza).
3. When you are ready to deliver an exercise program to a patient, turn to the page for the exercise program that you will deliver.

Patient #1	Study ID:	**P3-EX** (page 3)	**ACSM-PAVS** (pages 4-8)	Check off when prescribed (✓)
Patient #2	Study ID:	**P3-EX** (page 3)	**ACSM-PAVS** (pages 4-8)	Check off when prescribed (✓)

Please hand-write the password you were provided during your orientation to access the Patient Health Profile PDF:

____

Prioritize Personalize Prescribe EXercise [P3-EX]

Before you begin, ensure you have the Patient Health Outcome Values PDF by your side as you complete P3-EX. If it is an electronic PDF, use the password on page 2 of this manual to open the PDF.

Follow the below **script** *to assist you with delivering P3-EX to the patient.*

What you will say is written in **bold.**

Start below the line.

I will now provide you with an exercise program using a web-based application. I will ask you a series of questions. Please wait a moment as I sign in.

STEP 1: UTILIZE THE P3-EX WEB-BASED APPLICATION

1. *Click on the link that you were provided to access the P3-EX web-based algorithm. The link is also here: https://p3-ex-ui.vercel.app*
2. *Log into your account using your email and password.*
3. *Create a new patient and proceed to follow the instructions.*

Proceed to Step 2.

STEP 2: PROVIDE THE EXERCISE PROGRAM

After you have completed P3-EX, print out the exercise program and hand it to the patient.

Here is your exercise program that I encourage you to perform for 12 weeks.

You will now receive oversight from the study coordinator. You can expect to receive an email from them for the next steps.

American College of Sports Medicine Physical Activity Vital Signs [ACSM-PAVS]

Follow the below **script** *to assist you with delivering ACSM-PAVS to the patient.*

What you will say is written in **bold.**

Start below the line.

I will now provide you with an exercise program using this instruction manual. I will ask you a series of questions.

STEP 1: CONDUCT SAFETY SCREENING

I will now apply the American College of Sports Medicine Exercise Preparticipation Screening Algorithm.

Question 1: Participation in regular exercise (circle Yes/No).

Have you performed planned, structured physical activity for at least 30-minutes at moderate* intensity on at least 3 days per week for at least the last 3-months? Yes / No

**moderate intensity, causes noticeable increases in heart rate and breathing.*

Question 2: Identifying known cardiovascular, diabetes, or renal disease (Mark Yes/No).

Have you had or do you currently have?

____ a heart attack
____ heart surgery, cardiac catheterization, or coronary angioplasty
____ pacemaker/implantable cardiac defibrillator/rhythm disturbance
____ heart valve disease
____ heart failure
____ heart transplantation
____ congenital heart disease
____ diabetes
____ renal disease

Continue to the next page.

Question 3: Identifying signs and symptoms of underlying cardiovascular or renal disease (Mark Yes/No).

Have you experienced any of the following signs/symptoms?

____ chest discomfort with exertion
____ unreasonable breathlessness
____ dizziness, fainting, blackouts
____ ankle swelling
____ unpleasant awareness of a forceful, rapid or irregular heart rate
____ burning or cramping sensations in your lower legs when walking short distance
____ known heart murmur

If your patient does not have signs or symptoms suggestive of cardiovascular or renal disease.

→ *Continue to the next page.*

If your patient has one or more signs or symptoms suggestive of cardiovascular or renal disease.

→ **Based on your health history, further medical evaluation is needed before you can begin regular exercise. I will inform the study coordinator who will let you know the next steps. I will not be able to provide you with an exercise program.** *Please* ***discontinue*** *this instruction manual. Do not provide the patient with an exercise program.*

Question 4: Is medical clearance recommended?

Use the answers above to complete the flow diagram.

Reference:

Health Care Providers' Action Guide. ACSM Exercise is Medicine. 2021. Available from: https://www.exerciseismedicine.org/wp-content/uploads/2021/02/EIM-Health-Care-Providers-Action-Guide-clickable-links.pdf

Figure on Page 5.

Copyright Notice: *Copyright ® 2021 Exercise is Medicine.*

* KNOWN DISEASE = cardiovascular, diabetes, or renal disease.

Is medical clearance recommended?

Circle your answer.	Yes	No

Go to the next page.

If ***No*** → **Based on your health history, it is safe for you to begin regular exercise at light to moderate intensity.** *Go to STEP 2 below.*

If ***Yes*** →

If your patient has diabetes but does not have cardiovascular or renal disease.

→ **Based on your health history, it is safe for you to begin regular exercise at light to moderate intensity.** *Go to STEP 2 below.*

STEP 2: PROVIDE THE EXERCISE PROGRAM

Proceed to the last page of this instruction manual, and complete questions 1, 2, and 3 on this page with the patient. Provide the last page to the patient.

Here is your exercise program that I encourage you to perform for 12 weeks.

You will now receive oversight from the study coordinator. You can expect to receive an email from them for the next steps.

Reference:

Physical Activity Vital Sign. ACSM Exercise is Medicine. 2025. Available from: https://www.exerciseismedicine.org/wp-content/uploads/2021/04/EIM-Physical-Activity-Vital-Sign.pdf

Copyright Notice: *Copyright ©2025 Exercise is Medicine.*
