# Supplementary material for: Feasibility and Acceptability of a Novel Algorithm for Physicians to Prescribe Personalized Exercise Prescriptions to Patients with Cardiovascular Disease Risk Factors: Study Protocol for an Exploratory Randomized Controlled Crossover Trial
Source: Healthcare (Basel). 2026 Jan 12;14(2):188. doi: 10.3390/healthcare14020188 (PMC12840794; doi:10.3390/healthcare14020188)
Supplement: Supplementary file 1 [file healthcare-14-00188-s001.zip › Supplementary File S3 - ExRx Information Packet.pdf]

Participant 12-Week Exercise Program Information Packet

Participant ID:

Date Provided:

12-Week Start Date:

12-Week End Date:

Principle Investigators:

Dr. Antonio Fernandez, MD, FACC, FAHA
Medical Director of Preventive Cardiology, Hartford Hospital

Dr. Linda Pescatello, PhD, FACSM
Board of Trustees Distinguished Professor of Kinesiology, University of Connecticut

Dr. Peter Robinson, MD
Assistant Professor of Cardiology, UConn Health

UConn Graduate Research Assistant:

Alexander Wright, MS
Email: Alexander.Wright@hhchealth.org
Phone: (860) 486-6814

This research is approved by the Hartford HealthCare Institutional Review Board, with reliance agreements at UConn Storrs and UConn Health.

Contents Page

Exercise Program for Physical Activity as a Vital Sign ... 3
- Exercise Type Definitions ... 4
- Exercise Type Examples ... 5
- Exercise Intensity Definitions ... 7
- How to Take Your Pulse ... 8
- Self-Monitor Your Physical Activity ... 8
- Guidelines for Exercise Progression ... 9
- Contact Details for the UConn Graduate Research Assistant ... 9

12 Week Exercise Program Progression Guidance ... 10
- Week 1 ... 11
- Week 2 ... 13
- Week 3 ... 15
- Week 4 ... 17
- Week 5 ... 19
- Week 6 ... 21
- Week 7 ... 23
- Week 8 ... 25
- Week 9 ... 27
- Week 10 ... 29
- Week 11 ... 31
- Week 12 ... 33

Exercise Program for Physical Activity as a Vital Sign

FITT	Aerobic	Resistance	Neuromotor*	Flexibility
Frequency	3-5 days per week	≥2 days per week	2 days per week	2-3 days per week
Intensity	**Moderate intensity:** you can talk comfortably but not sing<br>64-76% HRmax; 40-59% VO_2R or HRR; RPE 12-13<br>to **vigorous intensity:** you cannot say more than 5 words without grasping for breath<br>77-95% HRmax; ≥60% VO_2R or HRR; RPE 14-17	**Moderate intensity:** 50-69% 1-RM for 12-18 repetitions<br>to **vigorous intensity:** 70-85% 1-RM for 8-12 repetitions	Undetermined	Stretch to the point of tightness or slight discomfort.
Time	150 minutes per week of moderate and/or 75 minutes per week of vigorous intensity physical activity (or an equivalent combination)	1-3 sets of 8-12 repetitions for each of the major muscle groups, 6-10 exercises per session	20-30 min per day	3 sets of 10-30 sec, 5-6 stretches, 2-4 repetitions of each exercise
Type	Prolonged, rhythmic activities using large muscle groups (e.g. walking, cycling, swimming)	Resistance machines and/or free weights	Exercise involving motor skills and/or functional body weight and flexibility exercise such as yoga, pilates, and tai chi	Static, dynamic, and/or PNF stretching

FITT = Frequency, Intensity, Time and Type; 1-RM = 1 Repetition Maximum; RPE = Rating of Perceived Exertion (Borg 6-20 scale); HRmax = Heart Rate Maximum; HRR = Heart Rate Reserve; VO_2R = Oxygen Uptake Reserve; PNF = Proprioceptive Neuromuscular Facilitation.

*Neuromotor functional body weight exercise can be substituted for resistance exercise, and depending on the amount of flexibility exercise integrated into a session, neuromotor flexibility exercise can be substituted for flexibility exercise depending on patient/client preference.

References:

ACSM. (2025). Physical Activity Vital Sign. Exercise is Medicine. https://www.exerciseismedicine.org/wp-content/uploads/2021/04/EIM-Physical-Activity-Vital-Sign.pdf

American College of Sports Medicine, Ozemek C, Bonikowske AR, Christle JW, Gallo PM, eds. ACSM's Guidelines for Exercise Testing and Prescription. 12th Ed, p.152-187. Wolters Kluwer; 2026.

Exercise Type Definitions

Aerobic Exercise: Continuous exercise involving large muscle groups such as walking, running, riding a bicycle, cardio machines and rowing.

Steady State Aerobic Exercise: Maintaining exercise at light to moderate intensity.

High Intensity Interval Training: Performing bouts of higher intensity exercise separated by rest.

Resistance Exercise: Exercises that use opposing forces to strengthen or develop muscles such as weight training with free weights, resistance machines, or using resistance bands or body weight.

Neuromotor Exercise: Exercises that combine balance, coordination, and agility such as yoga, tai chi, and Pilates.

Flexibility: Movements that improve the range of motion of a joint.

Dynamic Flexibility: Slow moving stretches that increase reach and range of motion as the movement is repeated. Examples: Leg swings, arm swings, torso twists.

Static Flexibility: Slowly stretching a muscle/tendon group and holding the position for 10-30 sec. Examples: Pike stretch, glute stretch, quad stretch

Proprioceptive Neuromuscular Facilitation (PNF): Applying force with a muscle against an opposing force followed by performing a static stretch of the muscle.

Concurrent Exercise: Performing aerobic and resistance exercise in the same session or near one another.

Major Muscle Groups: Shoulders, chest, back, hamstrings, quadriceps, calves, biceps, triceps, core.

Exercise Type Examples

Aerobic Exercise

Light Intensity (RPE 8-11)	Moderate Intensity (RPE 12-13)	Vigorous Intensity (RPE 14-20)
Walking slowly, leisurely	Walking the dog or walking outside	Brisk walking or jogging
Cycling slowly with a pedal desk	Cycling at a self-selected comfortable pace	Cycling at a moderate pace
Group class - Vinyasa yoga, tai chi (qi gong), or stretching	Group class – Power yoga or tai chi (yang style)	Group class - Zumba or fast ballroom dancing
Cleaning, sweeping, or washing dishes slowly	Water aerobics	Swimming laps, freestyle
Pickleball and Tennis, serving practice	Pickleball and Tennis, doubles	Pickleball and Tennis, singles

Resistance Exercise

Light Intensity (RPE 8-11)	Moderate Intensity (RPE 12-13)	Vigorous Intensity (RPE 14-20)
Yard work at a slow pace	Gardening - watering, weeding, planting	Gardening - using heavy tools, digging or filling garden
Canoeing at a slow pace	Kayaking at a self-selected comfortable pace	Rowing on a stationary ergometer
Group class - Vinyasa yoga, tai chi (qi gong)	Group class – Power yoga or tai chi (yang style)	Group class - circuit training with minimal rest
Bodyweight exercises (curl-ups, planks)	Bodyweight exercises (push-ups, lunges)	Bodyweight exercises (jumping jacks, burpees)
Lifting weights <50% of one repetition maximum (being able to perform 15-20 reps at this weight)	Lifting weights 50-69% of one repetition maximum (being able to perform 12-18 reps at this weight)	Lifting weights 70 to 85% of one repetition maximum (being able to perform 8-12 reps at this weight)

Neuromotor Exercise

**Light to Moderate Intensity (RPE 8-13)**	**Moderate Intensity (RPE 12-13)**
Yoga (Hatha sitting/Vinyasa/ Nadisodhana)	Yoga (power/Surya Namaskar)
Tai chi (qi gong, sitting)	Tai chi (qi gong standing/yang style)
Pilates (traditional)	Pilates (POUND® with drumming)
Balance exercises (beginner)	Balance exercises (general)
Slow dancing	Functional bodyweight exercises

Exercise Intensity Definitions

Adapted Version of Borg 6-20 Rating of Perceived Exertion (RPE) Scale with Exercise Intensity Differentiation:

Borg 6-20 RPE	Intensity	Intensity Definition
6	No exertion	
7	Very Light	
8		
9	Light	A level of physical exertion that causes slight increases in heart rate and breathing (i.e., warm up with dynamic flexibility, cool down with slow aerobic movements and static stretching). ***Use the talk test:*** *Light intensity should make your breathing slightly increase, but you can still talk and sing easily.*<br>Aerobic exercise: <64% HRmax, <40% VO_2R or HRR
10		
11		
12	Moderate	A level of physical exertion that causes increases in heart rate and breathing (i.e., brisk walking, weight training at an intensity that is not hard, but takes effort). ***Use the talk test:*** *Moderate intensity should make your breathing rate increase noticeably. You can still talk but not sing.*<br>Aerobic exercise: 64-76% HRmax, 40-59% VO_2R or HRR.<br>Resistance exercise: 50-69% of 1-RM intensity of 12-18 reps.
13		
14	Vigorous	A level of physical exertion that causes substantial increases in heart rate and breathing (i.e., running, weight training until fatigue). ***Use the talk test:*** *Vigorous intensity should make you breathe hard enough that you can only say a few words before you have to take a breath and you can't sing.*<br>Aerobic exercise: 77-95% HRmax, ≥ 60% VO_2R or HRR<br>Resistance exercise: 70-85% of 1-RM intensity of 8-12 reps.
15		
16		
17		
18	Near Maximal to Maximal	
19		
20		

HRmax = Heart Rate Maximum. The HRmax is the maximum heart rate you can achieve during exercise. HRmax declines with age.

HRR = Heart Rate Reserve. The percentage of HRR is calculated as: (the heart rate during exercise – the heart rate at rest) ÷ (the maximum heart rate during exercise – the heart rate at rest) × 100%.

VO_2R = Oxygen Uptake Reserve. The percentage of VO_2R is calculated as: (the rate of oxygen consumption during exercise – the rate of oxygen consumption at rest) ÷ (the maximum rate of oxygen consumption during exercise – the rate of oxygen consumption at rest) × 100%.

1-RM = 1 Repetition Maximum. The 1-RM is the maximum weight lifted for a single repetition for a given exercise.

How to Take Your Pulse

1) You can use a technique called pulse palpitation, which involves "feeling" the pulse.
2) Place your index finger and middle fingers over the radial artery, located near the thump side of the wrist.
3) Count the pulse for 30-60 seconds. The 30-second count is multiplied by 2 to determine the 1-minute resting heart rate in beats per minute (bpm).

Self-Monitor Your Physical Activity

1) The *Timeline Followback for Exercise* is a self-report tool for exercise and will be completed weekly.

2) Please fill out each day of the week in terms of the exercise you perform to the best of your abilities as described below:

   - **Did you exercise?** At any point in the day, did you exercise? This is answered as "yes" or "no". This includes both planned exercise and any other physical activity that is completed that day.
   - **Type(s):** For each type of exercise bout you completed that day, record the type(s) of the exercise you performed (i.e., walking, swimming, weightlifting etc.)
   - **Time (minutes):** For each type of exercise bout you completed that day, record how long it took you to complete the bout in minutes. Time for each exercise should be listed in the same order that it was listed for type(s) of exercise.
   - **Borg Rating of Perceived Exertion (RPE) on a scale of 6 to 20:** Record the Borg RPE for each exercise bout you completed that day using the scale and instructions below. RPE for each exercise should be listed in the same order that it was listed for type(s) of exercise.

   Please refer to the *Timeline Followback for Exercise* for further instructions.

3) For each week of the exercise program, transfer your recordings to an electronic diary in REDCap by the first day of each week (Sundays by 11:59 PM).

Guidelines for Exercise Progression

Aerobic training:

"Start low and go slow"

1) Start at light-to-moderate intensity exercise
2) Increase exercise duration (time) per day by 5-10 min every 1-2 weeks.
3) Increase the number of days per week gradually over 12 weeks.
4) Increase exercise intensity when you perceive reductions in your exertion during exercise sessions and gradually transition to vigorous intensity exercise.

Resistance training:

1) When you can perform 2 more repetitions than what was prescribed during two consecutive sessions for a given exercise, increase the load by 2.5%-5%, all while maintaining proper form/technique.
2) Increase the number of days per week the muscle groups are trained over 12 weeks.
3) Increase the number of sets per muscle group per session gradually as tolerated.

Neuromotor training:

1) Increase exercise intensity by performing more challenging or advanced balances, postures, or movements over 12 weeks.

Flexibility training:

1) Increase the number of days per week of stretching over 12 weeks.

Contact Details for the UConn Graduate Research Assistant

Alexander Wright

Email: Alexander.Wright@hhchealth.org

Phone: (860) 486-6814

12 Week Exercise Program Progression Guidance

<table>
<tr><th></th><th>Aerobic</th><th>Resistance</th><th>Neuromotor</th><th>Flexibility</th></tr>
<tr><td>Week 1</td><td rowspan="2">3 days per week at light to moderate intensity for ~30 minutes</td><td rowspan="3">1 day per week at moderate intensity for ~30 minutes</td><td rowspan="4">1 day per week at light to moderate intensity for 20-30 minutes</td><td rowspan="6">2 days per week for 5 minutes</td></tr>
<tr><td>Week 2</td></tr>
<tr><td>Week 3</td><td rowspan="2">3 days per week at light to moderate intensity for 30-40 minutes</td></tr>
<tr><td>Week 4</td><td rowspan="3">2 days per week at moderate intensity for ~30 minutes</td></tr>
<tr><td>Week 5</td><td rowspan="2">3 days per week at moderate intensity for 30-50 minutes</td><td rowspan="8">2 days per week at moderate intensity for 20-30 minutes</td></tr>
<tr><td>Week 6</td></tr>
<tr><td>Week 7</td><td rowspan="3">3-4 days per week at moderate intensity for 30-50 minutes and/or vigorous intensity for 15-25 minutes</td><td rowspan="6">≥2 days per week at moderate to vigorous intensity for ~45 minutes</td><td rowspan="6">3 days per week for 5 minutes</td></tr>
<tr><td>Week 8</td></tr>
<tr><td>Week 9</td></tr>
<tr><td>Week 10</td><td rowspan="3">3-5 days per week at moderate intensity for 30-60 minutes and/or vigorous intensity for 15-30 minutes</td></tr>
<tr><td>Week 11</td></tr>
<tr><td>Week 12</td></tr>
</table>

Week 1

Aerobic exercise: 3 days per week for ~30 minutes* per day at light to moderate intensity, to total 90 minutes per week. **Can be accumulated in multiple bouts or sessions over the day.*

Resistance exercise: 1 day per week for ~30 minutes per session at moderate intensity.

Neuromotor exercise: 1 day per week for 20-30 minutes per session at light to moderate intensity.

Flexibility exercise: 2 days per week for ~5 minutes per session*, stretching to the point of tightness or slight discomfort. **Can be included in warm-ups or cool-downs.*

Aerobic Exercise Workout Examples at Light to Moderate Intensity Week 1.

Component	Time	Type	Examples
1) Warm-up	5 minutes	Aerobic activity or dynamic stretching	- Walking at a talking pace on a treadmill<br>- Walking to the gym<br>- Dynamic stretching (3-6 sets of 30-90 sec with 15 sec rest periods between sets):<br>• Marching in place<br>•<br>• Walking lunges<br>• Hip Circles or openers<br>• Arm swings/arm circles<br>• Torso twists
2) Aerobic Exercise	~30 minutes	Steady state light to moderate intensity aerobic activity	- Walking at a pace that increases your breathing rate noticeably (~3000 steps)<br>- Peddling slowly on a stationary bicycle or leisurely cycling<br>- Peddling slowly on an elliptical machine<br>- Rowing slowly on a machine<br>- Slow dancing<br>- Aqua-aerobics
3) Cool-down	5 minutes	Static flexibility OR slowly reduce the pace of aerobic exercise	- Static stretching (10-30 seconds per stretch, 2-4 repetitions of each exercise):<br>• Standing calf stretch<br>• Quadricep stretch<br>• Kneeling hip flexor stretch (iliopsoas)<br>• Seated hamstring stretch<br>• Child's pose stretch<br>• Chest stretch<br>• Cross-body shoulder stretch<br>• Neck lateral flexion stretch

Resistance Exercise Workout Examples at Moderate Intensity Week 1.

Component	Time	Type	Example exercises with machines, free weights, bodyweight, or resistance bands
Resistance Exercise (Day 1)	~30 minutes	Resistance training for all major muscle groups	1-2 sets of 8-12 repetitions (rest 1.5-2 minutes between sets)<br>RPE 12-13; 1-RM: 50-69%<br>• Chest press<br>• Seated row<br>• Leg press<br>• Hip bridges<br>• Leg curls<br>• Arm curls<br>• Abdominal flexions<br>• Planks
Resistance Exercise (Day 2)	~30 minutes	Resistance training for all major muscle groups	1-2 sets of 8-12 repetitions (rest 1.5-2 minutes between sets):<br>RPE 12-13; 1-RM: 50-69%<br>• Shoulder press<br>• Lat pull-downs<br>• Hip kickbacks<br>• Hip abductions<br>• Leg extensions<br>• Triceps extensions<br>• Abdominal twists<br>• Side planks

Neuromotor Exercise Workout Examples at Light to Moderate Intensity Week 1.

Component	Time	Type	Examples
Neuromotor Exercise	20-30 minutes	Training that involves motor skills such as balance, coordination, agility	- Yoga (Hatha sitting/Vinyasa/ Nadisodhana)<br>- Tai chi (qi gong, sitting)<br>- Pilates (traditional)<br>- Balance exercise (15-30 seconds per hold, 5-6 cycles):<br>• two-legged stance<br>• semi-tandem stance<br>• reaching from a narrow stance<br>• tandem walking<br>• standing with eyes closed

Week 2

Aerobic exercise: 3 days per week for ~30 minutes* per day at light to moderate intensity, to total 90 minutes per week. **Can be accumulated in multiple bouts or sessions over the day.*

Resistance exercise: 1 day per week for ~30 minutes per session at moderate intensity.

Neuromotor exercise: 1 day per week for 20-30 minutes per session at light to moderate intensity.

Flexibility exercise: 2 days per week for ~5 minutes per session*, stretching to the point of tightness or slight discomfort. **Can be implemented into warm-ups or cool-downs.*

Aerobic Exercise Workout Examples at Light to Moderate Intensity Week 2.

Component	Time	Type	Examples
1) Warm-up	5 minutes	Aerobic activity or dynamic stretching	- Walking at a talking pace on a treadmill<br>- Walking to the gym<br>- Dynamic stretching (3-6 sets of 30-90 sec with 15 sec rest periods between sets):<br>• Marching in place<br>• Walking lunges<br>• Hip Circles or openers<br>• Arm swings/arm circles<br>• Torso twists
2) Aerobic Exercise	~30 minutes	Steady state light to moderate intensity aerobic activity	- Walking at a pace that increases your breathing rate noticeably (~3000 steps)<br>- Peddling slowly on a stationary bicycle or leisurely cycling<br>- Peddling slowly on an elliptical machine<br>- Rowing slowly on a machine<br>- Slow dancing<br>- Aqua-aerobics
3) Cool-down	5 minutes	Static flexibility OR slowly reduce the pace of aerobic exercise	- Static stretching (10-30 seconds per stretch, 2-4 repetitions of each exercise):<br>• Standing calf stretch<br>• Quadricep stretch<br>• Kneeling hip flexor stretch (iliopsoas)<br>• Seated hamstring stretch<br>• Child's pose stretch<br>• Chest stretch<br>• Cross-body shoulder stretch<br>• Neck lateral flexion stretch

Resistance Exercise Workout Examples at Moderate Intensity Week 2.

Component	Time	Type	Example exercises with machines, free weights, bodyweight, or resistance bands
Resistance Exercise (Day 1)	~30 minutes	Resistance training for all major muscle groups	1-2 sets of 8-12 repetitions (rest 1.5-2 minutes between sets)<br>RPE 12-13; 1-RM: 50-69%<br>• Chest press<br>• Seated row<br>• Leg press<br>• Hip bridges<br>• Leg curls<br>• Arm curls<br>• Abdominal flexions<br>• Planks
Resistance Exercise (Day 2)	~30 minutes	Resistance training for all major muscle groups	1-2 sets of 8-12 repetitions (rest 1.5-2 minutes between sets)<br>RPE 12-13; 1-RM: 50-69%<br>• Shoulder press<br>• Lat pull-downs<br>• Hip kickbacks<br>• Hip abductions<br>• Leg extensions<br>• Triceps extensions<br>• Abdominal twists<br>• Side planks

Neuromotor Exercise Workout Examples at Light to Moderate Intensity Week 2.

Component	Time	Type	Examples
Neuromotor Exercise	20-30 minutes	Training that involves motor skills such as balance, coordination, agility	- Yoga (Hatha sitting/Vinyasa/ Nadisodhana)<br>- Tai chi (qi gong, sitting)<br>- Pilates (traditional)<br>- Balance exercise (15-30 seconds per hold, 5-6 cycles):<br>• two-legged stance<br>• semi-tandem stance<br>• reaching from a narrow stance<br>• tandem walking<br>• standing with eyes closed

Week 3

Aerobic exercise: 3 days per week for 30-40 minutes* per day at light to moderate intensity, to total 105 minutes per week. **Can be accumulated in multiple bouts or sessions over the day.*

Resistance exercise: 1 day per week for ~30 minutes per session at moderate intensity.

Neuromotor exercise: 1 day per week for 20-30 minutes per session at light to moderate intensity.

Flexibility exercise: 2 days per week for ~5 minutes per session*, stretching to the point of tightness or slight discomfort. **Can be implemented into warm-ups or cool-downs.*

Aerobic Exercise Workout Examples at Light to Moderate Intensity Week 3.

Component	Time	Type	Examples
1) Warm-up	5 minutes	Aerobic activity or dynamic stretching	- Walking at a talking pace on a treadmill<br>- Walking to the gym<br>- Dynamic stretching (3-6 sets of 30-90 sec with 15 sec rest periods between sets):<br>• Marching in place<br>• Walking lunges<br>• Hip Circles or openers<br>• Arm swings/arm circles<br>• Torso twists
2) Aerobic Exercise	30-40 minutes	Steady state light to moderate intensity aerobic activity	- Walking at a pace that increases your breathing rate noticeably (~3000 steps)<br>- Peddling slowly on a stationary bicycle or leisurely cycling<br>- Peddling slowly on an elliptical machine<br>- Rowing slowly on a machine<br>- Slow dancing<br>- Aqua-aerobics
3) Cool-down	5 minutes	Static flexibility OR slowly reduce the pace of aerobic exercise	- Static stretching (10-30 seconds per stretch, 2-4 repetitions of each exercise):<br>• Standing calf stretch<br>• Quadricep stretch<br>• Kneeling hip flexor stretch (iliopsoas)<br>• Seated hamstring stretch<br>• Child's pose stretch<br>• Chest stretch<br>• Cross-body shoulder stretch<br>• Neck lateral flexion stretch

Resistance Exercise Workout Examples at Moderate Intensity Week 3.

Component	Time	Type	Example exercises with machines, free weights, bodyweight, or resistance bands
Resistance Exercise (Day 1)	~30 minutes	Resistance training for all major muscle groups	1-2 sets of 8-12 repetitions (rest 1.5-2 minutes between sets)<br>RPE 12-13; 1-RM: 50-69%<br>• Chest press<br>• Seated row<br>• Leg press<br>• Hip bridges<br>• Leg curls<br>• Arm curls<br>• Abdominal flexions<br>• Planks
Resistance Exercise (Day 2)	~30 minutes	Resistance training for all major muscle groups	1-2 sets of 8-12 repetitions (rest 1.5-2 minutes between sets)<br>RPE 12-13; 1-RM: 50-69%<br>• Shoulder press<br>• Lat pull-downs<br>• Hip kickbacks<br>• Hip abductions<br>• Leg extensions<br>• Triceps extensions<br>• Abdominal twists<br>• Side planks

Neuromotor Exercise Workout Examples at Light to Moderate Intensity Week 3.

Component	Time	Type	Examples
Neuromotor Exercise	20-30 minutes	Training that involves motor skills such as balance, coordination, agility	- Yoga (Hatha sitting/Vinyasa/ Nadisodhana)<br>- Tai chi (qi gong, sitting)<br>- Pilates (traditional)<br>- Balance exercise (15-30 seconds per hold, 5-6 cycles):<br>• two-legged stance<br>• semi-tandem stance<br>• reaching from a narrow stance<br>• tandem walking<br>• standing with eyes closed

Week 4

Aerobic exercise: 3 days per week for 30-40 minutes* per day at moderate intensity, to total 105 minutes per week. **Can be accumulated in multiple bouts or sessions over the day.*

Resistance exercise: 2 days per week for ~30 minutes per session at moderate intensity.

Neuromotor exercise: 1 day per week for 20-30 minutes per session at light to moderate intensity.

Flexibility exercise: 2 days per week for ~5 minutes per session*, stretching to the point of tightness or slight discomfort. **Can be implemented into warm-ups or cool-downs.*

Aerobic Exercise Workout Examples at Moderate Intensity Week 4.

Component	Time	Type	Examples
1) Warm-up	5 minutes	Aerobic activity or dynamic stretching	- Walking at a talking pace on a treadmill<br>- Walking to the gym<br>- Dynamic stretching (3-6 sets of 30-90 sec with 15 sec rest periods between sets):<br>• Marching in place<br>• Walking lunges<br>• Hip Circles or openers<br>• Arm swings/arm circles<br>• Torso twists
2) Aerobic Exercise	30-40 minutes	Steady state moderate intensity aerobic activity	- Walking at a pace that increases your breathing rate noticeably (~3000 steps)<br>- Peddling slowly on a stationary bicycle or leisurely cycling<br>- Peddling slowly on an elliptical machine<br>- Rowing slowly on a machine<br>- Slow dancing<br>- Aqua-aerobics
3) Cool-down	5 minutes	Static flexibility OR slowly reduce the pace of aerobic exercise	- Static stretching (10-30 seconds per stretch, 2-4 repetitions of each exercise):<br>• Standing calf stretch<br>• Quadricep stretch<br>• Kneeling hip flexor stretch (iliopsoas)<br>• Seated hamstring stretch<br>• Child's pose stretch<br>• Chest stretch<br>• Cross-body shoulder stretch<br>• Neck lateral flexion stretch

Resistance Exercise Workout Examples at Moderate Intensity Week 4.

Component	Time	Type	Example exercises with machines, free weights, bodyweight, or resistance bands
Resistance Exercise (Day 1)	~30 minutes	Resistance training for all major muscle groups	1-2 sets of 8-12 repetitions (rest 1.5-2 minutes between sets)<br>RPE 12-13; 1-RM: 50-69%<br>• Chest press<br>• Seated row<br>• Leg press<br>• Hip bridges<br>• Leg curls<br>• Arm curls<br>• Abdominal flexions<br>• Planks
Resistance Exercise (Day 2)	~30 minutes	Resistance training for all major muscle groups	1-2 sets of 8-12 repetitions (rest 1.5-2 minutes between sets)<br>RPE 12-13; 1-RM: 50-69%<br>• Shoulder press<br>• Lat pull-downs<br>• Hip kickbacks<br>• Hip abductions<br>• Leg extensions<br>• Triceps extensions<br>• Abdominal twists<br>• Side planks

Neuromotor Exercise Workout Examples at Light to Moderate Intensity Week 4.

Component	Time	Type	Examples
Neuromotor Exercise	20-30 minutes	Training that involves motor skills such as balance, coordination, agility	- Yoga (Hatha sitting/Vinyasa/ Nadisodhana)<br>- Tai chi (qi gong, sitting)<br>- Pilates (traditional)<br>- Balance exercise (15-30 seconds per hold, 5-6 cycles):<br>• two-legged stance<br>• semi-tandem stance<br>• reaching from a narrow stance<br>• tandem walking<br>• standing with eyes closed

Week 5

Aerobic exercise: 3 days per week for 30-50 minutes* per day at moderate intensity, to total 120 minutes per week. **Can be accumulated in multiple bouts or sessions over the day.*

Resistance exercise: 2 days per week for ~30 minutes per session at moderate intensity.

Neuromotor exercise: 2 days per week for 20-30 minutes per session at moderate intensity.

Flexibility exercise: 2 days per week for ~5 minutes per session*, stretching to the point of tightness or slight discomfort. **Can be implemented into warm-ups or cool-downs.*

Aerobic Exercise Workout Examples at Moderate Intensity Week 5.

Component	Time	Type	Examples
1) Warm-up	5 minutes	Aerobic activity or dynamic stretching	- Walking at a talking pace on a treadmill<br>- Walking to the gym<br>- Dynamic stretching (3-6 sets of 30-90 sec with 15 sec rest periods between sets):<br>• Marching in place<br>• Walking lunges<br>• Hip Circles or openers<br>• Arm swings/arm circles<br>• Torso twists
2) Aerobic Exercise	30-50 minutes	Steady state moderate intensity aerobic activity	- Walking at a pace that increases your breathing rate noticeably (~3000 steps)<br>- Peddling slowly on a stationary bicycle or leisurely cycling<br>- Peddling slowly on an elliptical machine<br>- Rowing slowly on a machine<br>- Slow dancing<br>- Aqua-aerobics
3) Cool-down	5 minutes	Static flexibility OR slowly reduce the pace of aerobic exercise	- Static stretching (10-30 seconds per stretch, 2-4 repetitions of each exercise):<br>• Standing calf stretch<br>• Quadricep stretch<br>• Kneeling hip flexor stretch (iliopsoas)<br>• Seated hamstring stretch<br>• Child's pose stretch<br>• Chest stretch<br>• Cross-body shoulder stretch<br>• Neck lateral flexion stretch

Resistance Exercise Workout Examples at Moderate Intensity Week 5.

Component	Time	Type	Example exercises with machines, free weights, bodyweight, or resistance bands
Resistance Exercise (Day 1)	~30 minutes	Resistance training for all major muscle groups	1-2 sets of 8-12 repetitions (rest 1.5-2 minutes between sets)<br>RPE 12-13; 1-RM: 50-69%<br>• Chest press<br>• Seated row<br>• Leg press<br>• Hip bridges<br>• Leg curls<br>• Arm curls<br>• Abdominal flexions<br>• Planks
Resistance Exercise (Day 2)	~30 minutes	Resistance training for all major muscle groups	1-2 sets of 8-12 repetitions (rest 1.5-2 minutes between sets)<br>RPE 12-13; 1-RM: 50-69%<br>• Shoulder press<br>• Lat pull-downs<br>• Hip kickbacks<br>• Hip abductions<br>• Leg extensions<br>• Triceps extensions<br>• Abdominal twists<br>• Side planks

Neuromotor Exercise Workout Examples at Light to Moderate Intensity Week 5.

Component	Time	Type	Examples
Neuromotor Exercise	20-30 minutes	Training that involves motor skills such as balance, coordination, agility	- Yoga (Hatha sitting/Vinyasa/ Nadisodhana)<br>- Tai chi (qi gong, sitting)<br>- Pilates (traditional)<br>- Balance exercise (15-30 seconds per hold, 5-6 cycles):<br>• two-legged stance<br>• semi-tandem stance<br>• reaching from a narrow stance<br>• tandem walking<br>• standing with eyes closed

Week 6

Aerobic exercise: 3 days per week for 30-50 minutes* per day at moderate intensity, to total 120 minutes per week. **Can be accumulated in multiple bouts or sessions over the day.*

Resistance exercise: 2 days per week for ~30 minutes per session at moderate intensity.

Neuromotor exercise: 2 days per week for 20-30 minutes per session at moderate intensity.

Flexibility exercise: 2 days per week for ~5 minutes per session*, stretching to the point of tightness or slight discomfort. **Can be implemented into warm-ups or cool-downs.*

Aerobic Exercise Workout Examples at Moderate Intensity Week 6.

Component	Time	Type	Examples
1) Warm-up	5 minutes	Aerobic activity or dynamic stretching	- Walking at a talking pace on a treadmill<br>- Walking to the gym<br>- Dynamic stretching (3-6 sets of 30-90 sec with 15 sec rest periods between sets):<br>• Marching in place<br>• Walking lunges<br>• Hip Circles or openers<br>• Arm swings/arm circles<br>• Torso twists
2) Aerobic Exercise	30-50 minutes	Steady state moderate intensity aerobic activity	- Walking at a pace that increases your breathing rate noticeably (~3000 steps)<br>- Peddling slowly on a stationary bicycle or leisurely cycling<br>- Peddling slowly on an elliptical machine<br>- Rowing slowly on a machine<br>- Slow dancing<br>- Aqua-aerobics
3) Cool-down	5 minutes	Static flexibility OR slowly reduce the pace of aerobic exercise	- Static stretching (10-30 seconds per stretch, 2-4 repetitions of each exercise):<br>• Standing calf stretch<br>• Quadricep stretch<br>• Kneeling hip flexor stretch (iliopsoas)<br>• Seated hamstring stretch<br>• Child's pose stretch<br>• Chest stretch<br>• Cross-body shoulder stretch<br>• Neck lateral flexion stretch

Resistance Exercise Workout Examples at Moderate Intensity Week 6.

Component	Time	Type	Example exercises with machines, free weights, bodyweight, or resistance bands
Resistance Exercise (Day 1)	~30 minutes	Resistance training for all major muscle groups	1-2 sets of 8-12 repetitions (rest 1.5-2 minutes between sets)<br>RPE 12-13; 1-RM: 50-69%<br>• Chest press<br>• Seated row<br>• Leg press<br>• Hip bridges<br>• Leg curls<br>• Arm curls<br>• Abdominal flexions<br>• Planks
Resistance Exercise (Day 2)	~30 minutes	Resistance training for all major muscle groups	1-2 sets of 8-12 repetitions (rest 1.5-2 minutes between sets)<br>RPE 12-13; 1-RM: 50-69%<br>• Shoulder press<br>• Lat pull-downs<br>• Hip kickbacks<br>• Hip abductions<br>• Leg extensions<br>• Triceps extensions<br>• Abdominal twists<br>• Side planks

Neuromotor Exercise Workout Examples at Light to Moderate Intensity Week 6.

Component	Time	Type	Examples
Neuromotor Exercise	20-30 minutes	Training that involves motor skills such as balance, coordination, agility	- Yoga (Hatha sitting/Vinyasa/ Nadisodhana)<br>- Tai chi (qi gong, sitting)<br>- Pilates (traditional)<br>- Balance exercise (15-30 seconds per hold, 5-6 cycles):<br>• two-legged stance<br>• semi-tandem stance<br>• reaching from a narrow stance<br>• tandem walking<br>• standing with eyes closed

Week 7

Aerobic exercise: 3-4 days per week for 30-50 minutes* per day at moderate intensity or 15-25 minutes per day at vigorous intensity (or a combination), to total 150 minutes per week. **Can be accumulated in multiple bouts or sessions over the day.*

Resistance exercise: ≥2 days per week for ~45 minutes per session at moderate to vigorous intensity.

Neuromotor exercise: 2 days per week for 20-30 minutes per session at moderate intensity.

Flexibility exercise: 2-3 days per week for ~5 minutes per session*, stretching to the point of tightness or slight discomfort. **Can be implemented into warm-ups or cool-downs.*

Aerobic Exercise Workout Examples at Moderate Intensity Week 7.

Component	Time	Type	Examples
1) Warm-up	5 minutes	Aerobic activity or dynamic stretching	- Walking at a talking pace on a treadmill<br>- Walking to the gym<br>- Dynamic stretching (3-6 sets of 30-90 sec with 15 sec rest periods between sets):<br>• Marching in place<br>• Walking lunges<br>• Hip Circles or openers<br>• Arm swings/arm circles<br>• Torso twists
2) Aerobic Exercise	30-50 minutes<br><br>OR<br><br>15-25 minutes	Steady state moderate intensity aerobic activity<br><br>OR<br><br>Steady state vigorous intensity aerobic activity or high intensity interval training	- Walking at a pace that increases your breathing rate noticeably (~3000 steps)<br>- Peddling slowly on a stationary bicycle or leisurely cycling<br>- Peddling slowly on an elliptical machine<br>- Rowing slowly on a machine<br>- Slow dancing<br>- Aqua-aerobics<br><br>- Walking very briskly, jogging, or running<br>- Peddling fast on a stationary bicycle or cycling fast or uphill<br>- Peddling fast on an elliptical machine<br>- Rowing fast on a machine<br>- Stepping on the stair stepper machine<br>- Fast dancing<br>- Aerobics
3) Cool-down	5 minutes	Static flexibility OR slowly reduce the pace of aerobic exercise	- Static stretching (10-30 seconds per stretch, 2-4 repetitions of each exercise):<br>• Standing calf stretch<br>• Quadricep stretch<br>• Kneeling hip flexor stretch (iliopsoas)<br>• Seated hamstring stretch<br>• Child's pose stretch

			• Chest stretch<br>• Cross-body shoulder stretch<br>• Neck lateral flexion stretch

Resistance Exercise Workout Examples at Moderate to Vigorous Intensity Week 7.

Component	Time	Type	Example exercises with machines, free weights, bodyweight, or resistance bands
Resistance Exercise (Day 1)	~45 minutes	Resistance training for all major muscle groups	2-3 sets of 8-12 repetitions (rest 1.5-2.5 minutes between sets)<br>RPE 12-13 to 14-17; 1-RM: 60-70% or more<br>• Chest press<br>• Seated row<br>• Leg press<br>• Hip bridges<br>• Leg curls<br>• Arm curls<br>• Abdominal flexions<br>• Planks
Resistance Exercise (Day 2)	~45 minutes	Resistance training for all major muscle groups	2-3 sets of 8-12 repetitions (rest 1.5-2.5 minutes between sets)<br>RPE 12-13 to 14-17; 1-RM: 50-85%<br>• Shoulder press<br>• Lat pull-downs<br>• Hip kickbacks<br>• Hip abductions<br>• Leg extensions<br>• Triceps extensions<br>• Abdominal twists<br>• Side planks
Resistance Exercise (Day 3)	~45 minutes	Resistance training for all major muscle groups	2-3 sets of 8-12 repetitions (rest 1.5-2.5 minutes between sets)<br>RPE 12-13 to 14-17; 1-RM: 50-85%<br>• Pick 6-10 exercises from above to train major muscle groups

Neuromotor Exercise Workout Examples at Moderate Intensity Week 7.

Component	Time	Type	Examples
Neuromotor Exercise	20-30 minutes	Training that involves motor skills such as balance, coordination, agility	- Yoga (power/Surya Namaskar)<br>- Tai chi (qi gong standing/yang style)<br>- Pilates (POUND® with drumming)<br>- Single leg or Bosu ball exercises<br>- Balance exercises (15-30 seconds per hold, 5-6 cycles):<br>• tandem stance<br>• one-legged stance<br>• stepping over obstacles<br>• heel or toe walks<br>• walking while turning the head

Week 8

Aerobic exercise: 3-4 days per week for 30-50 minutes* per day at moderate intensity or 15-25 minutes per day at vigorous intensity (or a combination), to total 150 minutes per week. **Can be accumulated in multiple bouts or sessions over the day.*

Resistance exercise: 2 days per week for ~45 minutes per session at moderate to vigorous intensity.

Neuromotor exercise: 2 days per week for 20-30 minutes per session at moderate intensity.

Flexibility exercise: 3 days per week for ~5 minutes per session*, stretching to the point of tightness or slight discomfort. **Can be implemented into warm-ups or cool-downs.*

Aerobic Exercise Workout Examples at Moderate Intensity Week 8.

Component	Time	Type	Examples
1) Warm-up	5 minutes	Aerobic activity or dynamic stretching	- Walking at a talking pace on a treadmill<br>- Walking to the gym<br>- Dynamic stretching (3-6 sets of 30-90 sec with 15 sec rest periods between sets):<br>• Marching in place<br>• Walking lunges<br>• Hip Circles or openers<br>• Arm swings/arm circles<br>• Torso twists
2) Aerobic Exercise	30-50 minutes<br><br><br><br><br><br><br>OR<br><br>15-25 minutes	Steady state moderate intensity aerobic activity<br><br><br><br>OR<br><br>Steady state vigorous intensity aerobic activity or high intensity interval training	- Walking at a pace that increases your breathing rate noticeably (~3000 steps)<br>- Peddling slowly on a stationary bicycle or leisurely cycling<br>- Peddling slowly on an elliptical machine<br>- Rowing slowly on a machine<br>- Slow dancing<br>- Aqua-aerobics<br><br>- Walking very briskly, jogging, or running<br>- Peddling fast on a stationary bicycle or cycling fast or uphill<br>- Peddling fast on an elliptical machine<br>- Rowing fast on a machine<br>- Stepping on the stair stepper machine<br>- Fast dancing<br>- Aerobics
3) Cool-down	5 minutes	Static flexibility OR slowly reduce the pace of aerobic exercise	- Static stretching (10-30 seconds per stretch, 2-4 repetitions of each exercise):<br>• Standing calf stretch<br>• Quadricep stretch<br>• Kneeling hip flexor stretch (iliopsoas)<br>• Seated hamstring stretch<br>• Child's pose stretch

			• Chest stretch<br>• Cross-body shoulder stretch<br>• Neck lateral flexion stretch

Resistance Exercise Workout Examples at Moderate to Vigorous Intensity Week 8.

Component	Time	Type	Example exercises with machines, free weights, bodyweight, or resistance bands
Resistance Exercise (Day 1)	~45 minutes	Resistance training for all major muscle groups	2-3 sets of 8-12 repetitions (rest 1.5-2.5 minutes between sets)<br>RPE 12-13 to 14-17; 1-RM: 50-85%<br>• Chest press<br>• Seated row<br>• Leg press<br>• Hip bridges<br>• Leg curls<br>• Arm curls<br>• Abdominal flexions<br>• Planks
Resistance Exercise (Day 2)	~45 minutes	Resistance training for all major muscle groups	2-3 sets of 8-12 repetitions (rest 1.5-2.5 minutes between sets)<br>RPE 12-13 to 14-17; 1-RM: 50-85%<br>• Shoulder press<br>• Lat pull-downs<br>• Hip kickbacks<br>• Hip abductions<br>• Leg extensions<br>• Triceps extensions<br>• Abdominal twists<br>• Side planks
Resistance Exercise (Day 3)	~45 minutes	Resistance training for all major muscle groups	2-3 sets of 8-12 repetitions (rest 1.5-2.5 minutes between sets)<br>RPE 12-13 to 14-17; 1-RM: 50-85%<br>• Pick 6-10 exercises from above to train major muscle groups

Neuromotor Exercise Workout Examples at Moderate Intensity Week 8.

Component	Time	Type	Examples
Neuromotor Exercise	20-30 minutes	Training that involves motor skills such as balance, coordination, agility	- Yoga (power/Surya Namaskar)<br>- Tai chi (qi gong standing/yang style)<br>- Pilates (POUND® with drumming)<br>- Single leg or Bosu ball exercises<br>- Balance exercises (15-30 seconds per hold, 5-6 cycles):<br>• tandem stance<br>• one-legged stance<br>• stepping over obstacles<br>• heel or toe walks<br>• walking while turning the head

Week 9

Aerobic exercise: 3-4 days per week for 30-50 minutes* per day at moderate intensity or 15-25 minutes per day at vigorous intensity (or a combination), to total 150 minutes per week. **Can be accumulated in multiple bouts or sessions over the day.*

Resistance exercise: 2 days per week for ~45 minutes per session at moderate to vigorous intensity.

Neuromotor exercise: 2 days per week for 20-30 minutes per session at moderate intensity.

Flexibility exercise: 3 days per week for ~5 minutes per session*, stretching to the point of tightness or slight discomfort. **Can be implemented into warm-ups or cool-downs.*

Aerobic Exercise Workout Examples at Moderate Intensity Week 9.

Component	Time	Type	Examples
1) Warm-up	5 minutes	Aerobic activity or dynamic stretching	- Walking at a talking pace on a treadmill<br>- Walking to the gym<br>- Dynamic stretching (3-6 sets of 30-90 sec with 15 sec rest periods between sets):<br>• Marching in place<br>• Walking lunges<br>• Hip Circles or openers<br>• Arm swings/arm circles<br>• Torso twists
2) Aerobic Exercise	30-50 minutes<br><br>OR<br><br>15-25 minutes	Steady state moderate intensity aerobic activity<br><br>OR<br><br>Steady state vigorous intensity aerobic activity or high intensity interval training	- Walking at a pace that increases your breathing rate noticeably (~3000 steps)<br>- Peddling slowly on a stationary bicycle or leisurely cycling<br>- Peddling slowly on an elliptical machine<br>- Rowing slowly on a machine<br>- Slow dancing<br>- Aqua-aerobics<br><br>- Walking very briskly, jogging, or running<br>- Peddling fast on a stationary bicycle or cycling fast or uphill<br>- Peddling fast on an elliptical machine<br>- Rowing fast on a machine<br>- Stepping on the stair stepper machine<br>- Fast dancing<br>- Aerobics
3) Cool-down	5 minutes	Static flexibility OR slowly reduce the pace of aerobic exercise	- Static stretching (10-30 seconds per stretch, 2-4 repetitions of each exercise):<br>• Standing calf stretch<br>• Quadricep stretch<br>• Kneeling hip flexor stretch (iliopsoas)<br>• Seated hamstring stretch<br>• Child's pose stretch

			• Chest stretch<br>• Cross-body shoulder stretch<br>• Neck lateral flexion stretch

Resistance Exercise Workout Examples at Moderate to Vigorous Intensity Week 9.

Component	Time	Type	Example exercises with machines, free weights, bodyweight, or resistance bands
Resistance Exercise (Day 1)	~45 minutes	Resistance training for all major muscle groups	2-3 sets of 8-12 repetitions (rest 1.5-2.5 minutes between sets)<br>RPE 12-13 to 14-17; 1-RM: 50-85%<br>• Chest press<br>• Seated row<br>• Leg press<br>• Hip bridges<br>• Leg curls<br>• Arm curls<br>• Abdominal flexions<br>• Planks
Resistance Exercise (Day 2)	~45 minutes	Resistance training for all major muscle groups	2-3 sets of 8-12 repetitions (rest 1.5-2.5 minutes between sets)<br>RPE 12-13 to 14-17; 1-RM: 50-85%<br>• Shoulder press<br>• Lat pull-downs<br>• Hip kickbacks<br>• Hip abductions<br>• Leg extensions<br>• Triceps extensions<br>• Abdominal twists<br>• Side planks
Resistance Exercise (Day 3)	~45 minutes	Resistance training for all major muscle groups	2-3 sets of 8-12 repetitions (rest 1.5-2.5 minutes between sets)<br>RPE 12-13 to 14-17; 1-RM: 50-85%<br>• Pick 6-10 exercises from above to train major muscle groups

Neuromotor Exercise Workout Examples at Moderate Intensity Week 9.

Component	Time	Type	Examples
Neuromotor Exercise	20-30 minutes	Training that involves motor skills such as balance, coordination, agility	- Yoga (power/Surya Namaskar)<br>- Tai chi (qi gong standing/yang style)<br>- Pilates (POUND® with drumming)<br>- Single leg or Bosu ball exercises<br>- Balance exercises (15-30 seconds per hold, 5-6 cycles):<br>• tandem stance<br>• one-legged stance<br>• stepping over obstacles<br>• heel or toe walks<br>• walking while turning the head

Week 10

Aerobic exercise: 3-5 days per week for 30-60 minutes* per day at moderate intensity or 15-30 minutes per day at vigorous intensity (or a combination), to total 150 minutes per week. **Can be accumulated in multiple bouts or sessions over the day.*

Resistance exercise: 2 days per week for ~45 minutes per session at moderate to vigorous intensity.

Neuromotor exercise: 2 days per week for 20-30 minutes per session at moderate intensity.

Flexibility exercise: 3 days per week for ~5 minutes per session*, stretching to the point of tightness or slight discomfort. **Can be implemented into warm-ups or cool-downs.*

Aerobic Exercise Workout Examples at Moderate Intensity Week 10.

Component	Time	Type	Examples
1) Warm-up	5 minutes	Aerobic activity or dynamic stretching	- Walking at a talking pace on a treadmill<br>- Walking to the gym<br>- Dynamic stretching (3-6 sets of 30-90 sec with 15 sec rest periods between sets):<br>• Marching in place<br>• Walking lunges<br>• Hip Circles or openers<br>• Arm swings/arm circles<br>• Torso twists
2) Aerobic Exercise	30-60 minutes<br><br>OR<br><br>15-30 minutes	Steady state moderate intensity aerobic activity<br><br>OR<br><br>Steady state vigorous intensity aerobic activity or high intensity interval training	- Walking at a pace that increases your breathing rate noticeably (~3000 steps)<br>- Peddling slowly on a stationary bicycle or leisurely cycling<br>- Peddling slowly on an elliptical machine<br>- Rowing slowly on a machine<br>- Slow dancing<br>- Aqua-aerobics<br><br>- Walking very briskly, jogging, or running<br>- Peddling fast on a stationary bicycle or cycling fast or uphill<br>- Peddling fast on an elliptical machine<br>- Rowing fast on a machine<br>- Stepping on the stair stepper machine<br>- Fast dancing<br>- Aerobics
3) Cool-down	5 minutes	Static flexibility OR slowly reduce the pace of aerobic exercise	- Static stretching (10-30 seconds per stretch, 2-4 repetitions of each exercise):<br>• Standing calf stretch<br>• Quadricep stretch<br>• Kneeling hip flexor stretch (iliopsoas)<br>• Seated hamstring stretch<br>• Child's pose stretch

			• Chest stretch<br>• Cross-body shoulder stretch<br>• Neck lateral flexion stretch

Resistance Exercise Workout Examples at Moderate to Vigorous Intensity Week 10.

Component	Time	Type	Example exercises with machines, free weights, bodyweight, or resistance bands
Resistance Exercise (Day 1)	~45 minutes	Resistance training for all major muscle groups	2-3 sets of 8-12 repetitions (rest 1.5-2.5 minutes between sets)<br>RPE 12-13 to 14-17; 1-RM: 50-85%<br>• Chest press<br>• Seated row<br>• Leg press<br>• Hip bridges<br>• Leg curls<br>• Arm curls<br>• Abdominal flexions<br>• Planks
Resistance Exercise (Day 2)	~45 minutes	Resistance training for all major muscle groups	2-3 sets of 8-12 repetitions (rest 1.5-2.5 minutes between sets)<br>RPE 12-13 to 14-17; 1-RM: 50-85%<br>• Shoulder press<br>• Lat pull-downs<br>• Hip kickbacks<br>• Hip abductions<br>• Leg extensions<br>• Triceps extensions<br>• Abdominal twists<br>• Side planks
Resistance Exercise (Day 3)	~45 minutes	Resistance training for all major muscle groups	2-3 sets of 8-12 repetitions (rest 1.5-2.5 minutes between sets)<br>RPE 12-13 to 14-17; 1-RM: 50-85%<br>• Pick 6-10 exercises from above to train major muscle groups

Neuromotor Exercise Workout Examples at Moderate Intensity Week 10.

Component	Time	Type	Examples
Neuromotor Exercise	20-30 minutes	Training that involves motor skills such as balance, coordination, agility	- Yoga (power/Surya Namaskar)<br>- Tai chi (qi gong standing/yang style)<br>- Pilates (POUND® with drumming)<br>- Single leg or Bosu ball exercises<br>- Balance exercises (15-30 seconds per hold, 5-6 cycles):<br>• tandem stance<br>• one-legged stance<br>• stepping over obstacles<br>• heel or toe walks<br>• walking while turning the head

Week 11

Aerobic exercise: 3-5 days per week for 30-60 minutes* per day at moderate intensity or 15-30 minutes per day at vigorous intensity (or a combination), to total 150 minutes per week. **Can be accumulated in multiple bouts or sessions over the day.*

Resistance exercise: 2 days per week for ~45 minutes per session at moderate to vigorous intensity.

Neuromotor exercise: 2 days per week for 20-30 minutes per session at moderate intensity.

Flexibility exercise: 3 days per week for ~5 minutes per session*, stretching to the point of tightness or slight discomfort. **Can be implemented into warm-ups or cool-downs.*

Aerobic Exercise Workout Examples at Moderate Intensity Week 11.

Component	Time	Type	Examples
1) Warm-up	5 minutes	Aerobic activity or dynamic stretching	- Walking at a talking pace on a treadmill<br>- Walking to the gym<br>- Dynamic stretching (3-6 sets of 30-90 sec with 15 sec rest periods between sets):<br>• Marching in place<br>• Walking lunges<br>• Hip Circles or openers<br>• Arm swings/arm circles<br>• Torso twists
2) Aerobic Exercise	30-60 minutes<br><br>OR<br><br>15-30 minutes	Steady state moderate intensity aerobic activity<br><br>OR<br><br>Steady state vigorous intensity aerobic activity or high intensity interval training	- Walking at a pace that increases your breathing rate noticeably (~3000 steps)<br>- Peddling slowly on a stationary bicycle or leisurely cycling<br>- Peddling slowly on an elliptical machine<br>- Rowing slowly on a machine<br>- Slow dancing<br>- Aqua-aerobics<br><br>- Walking very briskly, jogging, or running<br>- Peddling fast on a stationary bicycle or cycling fast or uphill<br>- Peddling fast on an elliptical machine<br>- Rowing fast on a machine<br>- Stepping on the stair stepper machine<br>- Fast dancing<br>- Aerobics
3) Cool-down	5 minutes	Static flexibility OR slowly reduce the pace of aerobic exercise	- Static stretching (10-30 seconds per stretch, 2-4 repetitions of each exercise):<br>• Standing calf stretch<br>• Quadricep stretch<br>• Kneeling hip flexor stretch (iliopsoas)<br>• Seated hamstring stretch<br>• Child's pose stretch

			• Chest stretch<br>• Cross-body shoulder stretch<br>• Neck lateral flexion stretch

Resistance Exercise Workout Examples at Moderate to Vigorous Intensity Week 11.

Component	Time	Type	Example exercises with machines, free weights, bodyweight, or resistance bands
Resistance Exercise (Day 1)	~45 minutes	Resistance training for all major muscle groups	2-3 sets of 8-12 repetitions (rest 1.5-2.5 minutes between sets)<br>RPE 12-13 to 14-17; 1-RM: 50-85%<br>• Chest press<br>• Seated row<br>• Leg press<br>• Hip bridges<br>• Leg curls<br>• Arm curls<br>• Abdominal flexions<br>• Planks
Resistance Exercise (Day 2)	~45 minutes	Resistance training for all major muscle groups	2-3 sets of 8-12 repetitions (rest 1.5-2.5 minutes between sets)<br>RPE 12-13 to 14-17; 1-RM: 50-85%<br>• Shoulder press<br>• Lat pull-downs<br>• Hip kickbacks<br>• Hip abductions<br>• Leg extensions<br>• Triceps extensions<br>• Abdominal twists<br>• Side planks
Resistance Exercise (Day 3)	~45 minutes	Resistance training for all major muscle groups	2-3 sets of 8-12 repetitions (rest 1.5-2.5 minutes between sets)<br>RPE 12-13 to 14-17; 1-RM: 50-85%<br>• Pick 6-10 exercises from above to train major muscle groups

Neuromotor Exercise Workout Examples at Moderate Intensity Week 11.

Component	Time	Type	Examples
Neuromotor Exercise	20-30 minutes	Training that involves motor skills such as balance, coordination, agility	- Yoga (power/Surya Namaskar)<br>- Tai chi (qi gong standing/yang style)<br>- Pilates (POUND® with drumming)<br>- Single leg or Bosu ball exercises<br>- Balance exercises (15-30 seconds per hold, 5-6 cycles):<br>• tandem stance<br>• one-legged stance<br>• stepping over obstacles<br>• heel or toe walks<br>• walking while turning the head

Week 12

Aerobic exercise: 3-5 days per week for 30-60 minutes* per day at moderate intensity or 15-30 minutes per day at vigorous intensity (or a combination), to total 150 minutes per week. **Can be accumulated in multiple bouts or sessions over the day.*

Resistance exercise: 2 days per week for ~45 minutes per session at moderate to vigorous intensity.

Neuromotor exercise: 2 days per week for 20-30 minutes per session at moderate intensity.

Flexibility exercise: 3 days per week for ~5 minutes per session*, stretching to the point of tightness or slight discomfort. **Can be implemented into warm-ups or cool-downs.*

Aerobic Exercise Workout Examples at Moderate Intensity Week 12.

Component	Time	Type	Examples
1) Warm-up	5 minutes	Aerobic activity or dynamic stretching	- Walking at a talking pace on a treadmill<br>- Walking to the gym<br>- Dynamic stretching (3-6 sets of 30-90 sec with 15 sec rest periods between sets):<br>• Marching in place<br>• Walking lunges<br>• Hip Circles or openers<br>• Arm swings/arm circles<br>• Torso twists
2) Aerobic Exercise	30-60 minutes<br><br>OR<br><br>15-30 minutes	Steady state moderate intensity aerobic activity<br><br>OR<br><br>Steady state vigorous intensity aerobic activity or high intensity interval training	- Walking at a pace that increases your breathing rate noticeably (~3000 steps)<br>- Peddling slowly on a stationary bicycle or leisurely cycling<br>- Peddling slowly on an elliptical machine<br>- Rowing slowly on a machine<br>- Slow dancing<br>- Aqua-aerobics<br><br>- Walking very briskly, jogging, or running<br>- Peddling fast on a stationary bicycle or cycling fast or uphill<br>- Peddling fast on an elliptical machine<br>- Rowing fast on a machine<br>- Stepping on the stair stepper machine<br>- Fast dancing<br>- Aerobics
3) Cool-down	5 minutes	Static flexibility OR slowly reduce the pace of aerobic exercise	- Static stretching (10-30 seconds per stretch, 2-4 repetitions of each exercise):<br>• Standing calf stretch<br>• Quadricep stretch<br>• Kneeling hip flexor stretch (iliopsoas)<br>• Seated hamstring stretch<br>• Child's pose stretch

			• Chest stretch<br>• Cross-body shoulder stretch<br>• Neck lateral flexion stretch

Resistance Exercise Workout Examples at Moderate to Vigorous Intensity Week 12.

Component	Time	Type	Example exercises with machines, free weights, bodyweight, or resistance bands
Resistance Exercise (Day 1)	~45 minutes	Resistance training for all major muscle groups	2-3 sets of 8-12 repetitions (rest 1.5-2.5 minutes between sets)<br>RPE 12-13 to 14-17; 1-RM: 50-85%<br>• Chest press<br>• Seated row<br>• Leg press<br>• Hip bridges<br>• Leg curls<br>• Arm curls<br>• Abdominal flexions<br>• Planks
Resistance Exercise (Day 2)	~45 minutes	Resistance training for all major muscle groups	2-3 sets of 8-12 repetitions (rest 1.5-2.5 minutes between sets)<br>RPE 12-13 to 14-17; 1-RM: 50-85%<br>• Shoulder press<br>• Lat pull-downs<br>• Hip kickbacks<br>• Hip abductions<br>• Leg extensions<br>• Triceps extensions<br>• Abdominal twists<br>• Side planks
Resistance Exercise (Day 3)	~45 minutes	Resistance training for all major muscle groups	2-3 sets of 8-12 repetitions (rest 1.5-2.5 minutes between sets)<br>RPE 12-13 to 14-17; 1-RM: 50-85%<br>• Pick 6-10 exercises from above to train major muscle groups

Neuromotor Exercise Workout Examples at Moderate Intensity Week 12.

Component	Time	Type	Examples
Neuromotor Exercise	20-30 minutes	Training that involves motor skills such as balance, coordination, agility	- Yoga (power/Surya Namaskar)<br>- Tai chi (qi gong standing/yang style)<br>- Pilates (POUND® with drumming)<br>- Single leg or Bosu ball exercises<br>- Balance exercises (15-30 seconds per hold, 5-6 cycles):<br>• tandem stance<br>• one-legged stance<br>• stepping over obstacles<br>• heel or toe walks<br>• walking while turning the head

Participant 12-Week Exercise Program Information Packet

Participant ID:

Date Provided:

12-Week Start Date:

12-Week End Date:

Principle Investigators:

Dr. Antonio Fernandez, MD, FACC, FAHA
Medical Director of Preventive Cardiology, Hartford Hospital

Dr. Linda Pescatello, PhD, FACSM
Board of Trustees Distinguished Professor of Kinesiology, University of Connecticut

Dr. Peter Robinson, MD
Assistant Professor of Cardiology, UConn Health

UConn Graduate Research Assistant:

Alexander Wright, MS
Email: Alexander.Wright@hhchealth.org
Phone: (860) 486-6814

This research is approved by the Hartford HealthCare Institutional Review Board, with reliance agreements at UConn Storrs and UConn Health.

Contents Page

Exercise Program for Obesity 3
Special Considerations 4
Exercise Type Definitions 5
Exercise Type Examples 6
Exercise Intensity Definitions 8
How to Take Your Pulse 9
Self-Monitor Your Physical Activity 9
Guidelines for Exercise Progression 10
Contact Details for the UConn Graduate Research Assistant 10
12 Week Exercise Program Progression Guidance 11
Week 1 12
Week 2 14
Week 3 16
Week 4 18
Week 5 20
Week 6 22
Week 7 24
Week 8 26
Week 9 28
Week 10 30
Week 11 32
Week 12 34

Exercise Program for Obesity

FITT	Aerobic	Resistance	Neuromotor*	Flexibility
Frequency	≥5 days per week	2-3 days per week	≥2-3 days per week	≥2-3 days per week
Intensity	Initial intensity should be **moderate intensity:** you can talk comfortably but not sing<br><br>64-76% HRmax; 40-59% VO2R or HRR; RPE 12-13<br><br>Progress to **vigorous intensity** for greater health benefits: you cannot say more than 5 words without grasping for breath<br><br>77-95% HRmax; ≥ 60% VO2R or HRR; RPE 14-17	**Moderate intensity:** 60-70% of 1-RM for 12-18 repetitions<br><br>Gradually increase to **vigorous intensity:** 80% 1-RM for 8-12 repetitions, to enhance strength and muscle mass.	Undetermined	Stretch to the point of tightness or slight discomfort.
Time	30 minutes per day (150 minutes per week); increase to 60 minutes per day or more (250-300 minutes per week).	2-4 sets of 8-12 repetitions for each of the major muscle groups.	≥20-30 min per day	Hold static stretch for 10-30 seconds; 2-4 repetitions of each exercise.
Type	Prolonged, rhythmic activities using large muscle groups (e.g. walking, cycling, swimming)	Resistance machines and/or free weights	Exercise involving motor skills and/or functional body weight and flexibility exercise such as yoga, Pilates, and tai chi.	Static, dynamic, and/or PNF stretching

FITT = Frequency, Intensity, Time and Type; 1-RM = 1 Repetition Maximum; RPE = Rating of Perceived Exertion (Borg 6-20 scale); HRmax = Heart Rate Maximum; HRR = Heart Rate Reserve; VO_2R = Oxygen Uptake Reserve; PNF = Proprioceptive Neuromuscular Facilitation.

*Neuromotor functional body weight exercise can be substituted for resistance exercise, and depending on the amount of flexibility exercise integrated into a session, neuromotor flexibility exercise can be substituted for flexibility exercise depending on patient/client preference.

Reference: American College of Sports Medicine, Ozemek C, Bonikowske AR, Christle JW, Gallo PM, eds. ACSM's Guidelines for Exercise Testing and Prescription. 12th Ed, p.370. Wolters Kluwer; 2026.

Special Considerations

Medication Considerations: Yes/No

Bariatric Surgery Considerations: Yes/No

If YES, will list medication(s) taken and dose:

If YES, will list potential side effects of medication or bariatric surgery as related to exercise.

If YES, will list special considerations for the medication or bariatric surgery as related to exercise.

Exercise Type Definitions

Aerobic Exercise: Continuous exercise involving large muscle groups such as walking, running, riding a bicycle, cardio machines and rowing.

Steady State Aerobic Exercise: Maintaining exercise at light to moderate intensity.

High Intensity Interval Training: Performing bouts of higher intensity exercise separated by rest.

Resistance Exercise: Exercises that use opposing forces to strengthen or develop muscles such as weight training with free weights, resistance machines, or using resistance bands or body weight.

Neuromotor Exercise: Exercises that combine balance, coordination, and agility such as yoga, tai chi, and Pilates.

Flexibility: Movements that improve the range of motion of a joint.

Dynamic Flexibility: Slow moving stretches that increase reach and range of motion as the movement is repeated. Examples: Leg swings, arm swings, torso twists.

Static Flexibility: Slowly stretching a muscle/tendon group and holding the position for 10-30 sec. Examples: Pike stretch, glute stretch, quad stretch

Proprioceptive Neuromuscular Facilitation (PNF): Applying force with a muscle against an opposing force followed by performing a static stretch of the muscle.

Concurrent Exercise: Performing aerobic and resistance exercise in the same session or near one another.

Major Muscle Groups: Shoulders, chest, back, hamstrings, quadriceps, calves, biceps, triceps, core.

Exercise Type Examples

Aerobic Exercise

Light Intensity (RPE 8-11)	Moderate Intensity (RPE 12-13)	Vigorous Intensity (RPE 14-20)
Walking slowly, leisurely	Walking the dog or walking outside	Brisk walking or jogging
Cycling slowly with a pedal desk	Cycling at a self-selected comfortable pace	Cycling at a moderate pace
Group class - Vinyasa yoga, tai chi (qi gong), or stretching	Group class – Power yoga or tai chi (yang style)	Group class - Zumba or fast ballroom dancing
Cleaning, sweeping, or washing dishes slowly	Water aerobics	Swimming laps, freestyle
Pickleball and Tennis, serving practice	Pickleball and Tennis, doubles	Pickleball and Tennis, singles

Resistance Exercise

Light Intensity (RPE 8-11)	Moderate Intensity (RPE 12-13)	Vigorous Intensity (RPE 14-20)
Yard work at a slow pace	Gardening - watering, weeding, planting	Gardening - using heavy tools, digging or filling garden
Canoeing at a slow pace	Kayaking at a self-selected comfortable pace	Rowing on a stationary ergometer
Group class - Vinyasa yoga, tai chi (qi gong)	Group class – Power yoga or tai chi (yang style)	Group class - circuit training with minimal rest
Bodyweight exercises (curl-ups, planks)	Bodyweight exercises (push-ups, lunges)	Bodyweight exercises (jumping jacks, burpees)
Lifting weights <50% of one repetition maximum (being able to perform 15-20 reps at this weight)	Lifting weights 50-69% of one repetition maximum (being able to perform 12-18 reps at this weight)	Lifting weights 70 to 85% of one repetition maximum (being able to perform 8-12 reps at this weight)

Neuromotor Exercise

**Light to Moderate Intensity (RPE 8-13)**	**Moderate Intensity (RPE 12-13)**
Yoga (Hatha sitting/Vinyasa/ Nadisodhana)	Yoga (power/Surya Namaskar)
Tai chi (qi gong, sitting)	Tai chi (qi gong standing/yang style)
Pilates (traditional)	Pilates (POUND® with drumming)
Balance exercises (beginner)	Balance exercises (general)
Slow dancing	Functional bodyweight exercises

Exercise Intensity Definitions

Adapted Version of Borg 6-20 Rating of Perceived Exertion (RPE) Scale with Exercise Intensity Differentiation:

Borg 6-20 RPE	Intensity	Intensity Definition
6	No exertion	
7	Very Light	
8		
9	Light	A level of physical exertion that causes slight increases in heart rate and breathing (i.e., warm up with dynamic flexibility, cool down with slow aerobic movements and static stretching). ***Use the talk test:*** *Light intensity should make your breathing slightly increase, but you can still talk and sing easily.*<br>Aerobic exercise: <64% HRmax, <40% VO_2R or HRR
10		
11		
12	Moderate	A level of physical exertion that causes increases in heart rate and breathing (i.e., brisk walking, weight training at an intensity that is not hard, but takes effort). ***Use the talk test:*** *Moderate intensity should make your breathing rate increase noticeably. You can still talk but not sing.*<br>Aerobic exercise: 64-76% HRmax, 40-59% VO_2R or HRR.<br>Resistance exercise: 50-69% of 1-RM intensity of 12-18 reps.
13		
14	Vigorous	A level of physical exertion that causes substantial increases in heart rate and breathing (i.e., running, weight training until fatigue). ***Use the talk test:*** *Vigorous intensity should make you breathe hard enough that you can only say a few words before you have to take a breath and you can't sing.*<br>Aerobic exercise: 77-95% HRmax, ≥ 60% VO_2R or HRR<br>Resistance exercise: 70-85% of 1-RM intensity of 8-12 reps.
15		
16		
17		
18	Near Maximal to Maximal	
19		
20		

HRmax = Heart Rate Maximum. The HRmax is the maximum heart rate you can achieve during exercise. HRmax declines with age.

HRR = Heart Rate Reserve. The percentage of HRR is calculated as: (the heart rate during exercise – the heart rate at rest) ÷ (the maximum heart rate during exercise – the heart rate at rest) × 100%.

VO_2R = Oxygen Uptake Reserve. The percentage of VO_2R is calculated as: (the rate of oxygen consumption during exercise – the rate of oxygen consumption at rest) ÷ (the maximum rate of oxygen consumption during exercise – the rate of oxygen consumption at rest) × 100%.

1-RM = 1 Repetition Maximum. The 1-RM is the maximum weight lifted for a single repetition for a given exercise.

How to Take Your Pulse

1) You can use a technique called pulse palpitation, which involves “feeling” the pulse.
2) Place your index finger and middle fingers over the radial artery, located near the thump side of the wrist.
3) Count the pulse for 30-60 seconds. The 30-second count is multiplied by 2 to determine the 1-minute resting heart rate in beats per minute (bpm).

Self-Monitor Your Physical Activity

1) The *Timeline Followback for Exercise* is a self-report tool for exercise and will be completed weekly.
2) Please fill out each day of the week in terms of the exercise you perform to the best of your abilities as described below:
   - **Did you exercise?** At any point in the day, did you exercise? This is answered as “yes” or “no”. This includes both planned exercise and any other physical activity that is completed that day.
   - **Type(s):** For each type of exercise bout you completed that day, record the type(s) of the exercise you performed (i.e., walking, swimming, weightlifting etc.)
   - **Time (minutes):** For each type of exercise bout you completed that day, record how long it took you to complete the bout in minutes. Time for each exercise should be listed in the same order that it was listed for type(s) of exercise.
   - **Borg Rating of Perceived Exertion (RPE) on a scale of 6 to 20:** Record the Borg RPE for each exercise bout you completed that day using the scale and instructions below. RPE for each exercise should be listed in the same order that it was listed for type(s) of exercise.

   Please refer to the *Timeline Followback for Exercise* for further instructions.
3) For each week of the exercise program, transfer your recordings to an electronic diary in REDCap by the first day of each week (Sundays by 11:59 PM).

Guidelines for Exercise Progression

Aerobic training:

"Start low and go slow"

1) Start at light-to-moderate intensity exercise
2) Increase exercise duration (time) per day by 5-10 min every 1-2 weeks.
3) Increase the number of days per week gradually over 12 weeks.
4) Increase exercise intensity when you perceive reductions in your exertion during exercise sessions and gradually transition to vigorous intensity exercise.

Resistance training:

1) When you can perform 2 more repetitions than what was prescribed during two consecutive sessions for a given exercise, increase the load by 2.5%-5%, all while maintaining proper form/technique.
2) Increase the number of days per week the muscle groups are trained over 12 weeks.
3) Increase the number of sets per muscle group per session gradually as tolerated.

Neuromotor training:

1) Increase exercise intensity by performing more challenging or advanced balances, postures, or movements over 12 weeks.

Flexibility training:

1) Increase the number of days per week of stretching over 12 weeks.

Contact Details for the UConn Graduate Research Assistant

Alexander Wright

Email: Alexander.Wright@hhchealth.org

Phone: (860) 486-6814

12 Week Exercise Program Progression Guidance

<table>
<tr><th></th><th>Aerobic</th><th>Resistance</th><th>Neuromotor</th><th>Flexibility</th></tr>
<tr><td>Week 1</td><td rowspan="2">3-4 days per week at light to moderate intensity for ~30 minutes</td><td rowspan="3">1 day per week at moderate intensity for ~30 minutes</td><td rowspan="4">1 day per week at light to moderate intensity for 20-30 minutes</td><td rowspan="6">2 days per week for 5 minutes</td></tr>
<tr><td>Week 2</td></tr>
<tr><td>Week 3</td><td rowspan="2">3-4 days per week at light to moderate intensity for 30-40 minutes</td></tr>
<tr><td>Week 4</td><td rowspan="3">2 days per week at moderate intensity for ~30 minutes</td></tr>
<tr><td>Week 5</td><td rowspan="2">3-4 days per week at moderate intensity for 30-50 minutes</td><td rowspan="8">2 days per week at moderate intensity for 20-30 minutes</td></tr>
<tr><td>Week 6</td></tr>
<tr><td>Week 7</td><td rowspan="3">4-5 days per week at moderate intensity for 30-50 minutes and/or vigorous intensity for 15-25 minutes</td><td rowspan="6">2-3 days per week at moderate to vigorous intensity for ~45 minutes</td><td rowspan="6">≥2-3 days per week for 5 minutes</td></tr>
<tr><td>Week 8</td></tr>
<tr><td>Week 9</td></tr>
<tr><td>Week 10</td><td rowspan="3">5-6 days per week at moderate intensity for 30-60 minutes and/or vigorous intensity for 15-30 minutes</td></tr>
<tr><td>Week 11</td></tr>
<tr><td>Week 12</td></tr>
</table>

Week 1

Aerobic exercise: 3-4 days per week for ~30 minutes* per day at light to moderate intensity, to total 90 minutes per week. **Can be accumulated in multiple daily bouts of at least 10 minutes in duration.*

Resistance exercise: 1 day per week for ~30 minutes per session at moderate intensity.

Neuromotor exercise: 1 day per week for 20-30 minutes per session at light to moderate intensity.

Flexibility exercise: 2 days per week for ~5 minutes per session*, stretching to the point of tightness or slight discomfort. **Can be included in warm-ups or cool-downs.*

Aerobic Exercise Workout Examples at Light to Moderate Intensity Week 1.

Component	Time	Type	Examples
1) Warm-up	5 minutes	Aerobic activity or dynamic stretching	- Walking at a talking pace on a treadmill<br>- Walking to the gym<br>- Dynamic stretching (3-6 sets of 30-90 sec with 15 sec rest periods between sets):<br>• Marching in place<br>• Walking lunges<br>• Hip circles or openers<br>• Arm swings/arm circles<br>• Torso twists
2) Aerobic Exercise	~30 minutes	Steady state moderate intensity aerobic activity	- Walking at a pace that increases your breathing rate noticeably<br>- Peddling slowly on a stationary bicycle or leisurely cycling<br>- Peddling slowly on an elliptical machine<br>- Rowing slowly on a machine<br>- Slow dancing<br>- Aqua-aerobics
3) Cool-down	5 minutes	Static flexibility OR slowly reduce the pace of aerobic exercise	- Static stretching (10-30 seconds per stretch, 2-4 repetitions of each exercise):<br>• Standing calf stretch<br>• Quadricep stretch<br>• Kneeling hip flexor stretch (iliopsoas)<br>• Seated hamstring stretch<br>• Child's pose stretch<br>• Chest stretch<br>• Cross-body shoulder stretch<br>• Neck lateral flexion stretch

Resistance Exercise Workout Examples at Moderate Intensity Week 1.

Component	Time	Type	Example exercises with machines, free weights, bodyweight, or resistance bands
Resistance Exercise (Day 1)	~30 minutes	Resistance training for all major muscle groups	2-3 sets of 8-12 repetitions (rest 1.5-2 minutes between sets)<br>RPE 12-13; 1-RM: 60-70%<br>• Chest press<br>• Seated row<br>• Leg press<br>• Hip bridges<br>• Leg curls<br>• Arm curls<br>• Abdominal flexions<br>• Planks
Resistance Exercise (Day 2)	~30 minutes	Resistance training for all major muscle groups	2-3 sets of 8-12 repetitions (rest 1.5-2 minutes between sets):<br>RPE 12-13; 1-RM: 60-70%<br>• Shoulder press<br>• Lat pull-downs<br>• Hip kickbacks<br>• Hip abductions<br>• Leg extensions<br>• Triceps extensions<br>• Abdominal twists<br>• Side planks

Neuromotor Exercise Workout Examples at Light to Moderate Intensity Week 1.

Component	Time	Type	Examples
Neuromotor Exercise	20-30 minutes	Training that involves motor skills such as balance, coordination, agility	- Yoga (Hatha sitting/Vinyasa/ Nadisodhana)<br>- Tai chi (qi gong, sitting)<br>- Pilates (traditional)<br>- Balance exercise (15-30 seconds per hold, 5-6 cycles):<br>• two-legged stance<br>• semi-tandem stance<br>• reaching from a narrow stance<br>• tandem walking<br>• standing with eyes closed

Week 2

Aerobic exercise: 3-4 days per week for ~30 minutes* per day at light to moderate intensity, to total 90 minutes per week. **Can be accumulated in multiple daily bouts of at least 10 minutes in duration.*

Resistance exercise: 1 day per week for ~30 minutes per session at moderate intensity.

Neuromotor exercise: 1 day per week for 20-30 minutes per session at light to moderate intensity.

Flexibility exercise: 2 days per week for ~5 minutes per session*, stretching to the point of tightness or slight discomfort. **Can be implemented into warm-ups or cool-downs.*

Aerobic Exercise Workout Examples at Light to Moderate Intensity Week 2.

Component	Time	Type	Examples
1) Warm-up	5 minutes	Aerobic activity or dynamic stretching	- Walking at a talking pace on a treadmill<br>- Walking to the gym<br>- Dynamic stretching (3-6 sets of 30-90 sec with 15 sec rest periods between sets):<br>• Marching in place<br>• Walking lunges<br>• Hip circles or owners<br>• Arm swings/arm circles<br>• Torso twists
2) Aerobic Exercise	~30 minutes	Steady state moderate intensity aerobic activity	- Walking at a pace that increases your breathing rate noticeably<br>- Peddling slowly on a stationary bicycle or leisurely cycling<br>- Peddling slowly on an elliptical machine<br>- Rowing slowly on a machine<br>- Slow dancing<br>- Aqua-aerobics
3) Cool-down	5 minutes	Static flexibility OR slowly reduce the pace of aerobic exercise	- Static stretching (10-30 seconds per stretch, 2-4 repetitions of each exercise):<br>• Standing calf stretch<br>• Quadricep stretch<br>• Kneeling hip flexor stretch (iliopsoas)<br>• Seated hamstring stretch<br>• Child's pose stretch<br>• Chest stretch<br>• Cross-body shoulder stretch<br>• Neck lateral flexion stretch

Resistance Exercise Workout Examples at Moderate Intensity Week 2.

Component	Time	Type	Example exercises with machines, free weights, bodyweight, or resistance bands
Resistance Exercise (Day 1)	~30 minutes	Resistance training for all major muscle groups	2-3 sets of 8-12 repetitions (rest 1.5-2 minutes between sets)<br>RPE 12-13; 1-RM: 60-70%<br>• Chest press<br>• Seated row<br>• Leg press<br>• Hip bridges<br>• Leg curls<br>• Arm curls<br>• Abdominal flexions<br>• Planks
Resistance Exercise (Day 2)	~30 minutes	Resistance training for all major muscle groups	2-3 sets of 8-12 repetitions (rest 1.5-2 minutes between sets)<br>RPE 12-13; 1-RM: 60-70%<br>• Shoulder press<br>• Lat pull-downs<br>• Hip kickbacks<br>• Hip abductions<br>• Leg extensions<br>• Triceps extensions<br>• Abdominal twists<br>• Side planks

Neuromotor Exercise Workout Examples at Light to Moderate Intensity Week 2.

Component	Time	Type	Examples
Neuromotor Exercise	20-30 minutes	Training that involves motor skills such as balance, coordination, agility	- Yoga (Hatha sitting/Vinyasa/ Nadisodhana)<br>- Tai chi (qi gong, sitting)<br>- Pilates (traditional)<br>- Balance exercise (15-30 seconds per hold, 5-6 cycles):<br>• two-legged stance<br>• semi-tandem stance<br>• reaching from a narrow stance<br>• tandem walking<br>• standing with eyes closed

Week 3

Aerobic exercise: 3-4 days per week for 30-40 minutes* per day at light to moderate intensity, to total 125 minutes per week. **Can be accumulated in multiple daily bouts of at least 10 minutes in duration.*

Resistance exercise: 1 day per week for ~30 minutes per session at moderate intensity.

Neuromotor exercise: 1 day per week for 20-30 minutes per session at light to moderate intensity.

Flexibility exercise: 2 days per week for ~5 minutes per session*, stretching to the point of tightness or slight discomfort. **Can be implemented into warm-ups or cool-downs.*

Aerobic Exercise Workout Examples at Light to Moderate Intensity Week 3.

Component	Time	Type	Examples
1) Warm-up	5 minutes	Aerobic activity or dynamic stretching	- Walking at a talking pace on a treadmill<br>- Walking to the gym<br>- Dynamic stretching (3-6 sets of 30-90 sec with 15 sec rest periods between sets):<br>• Marching in place<br>• Walking lunges<br>• Hip circles or owners<br>• Arm swings/arm circles<br>• Torso twists
2) Aerobic Exercise	30-40 minutes	Steady state moderate intensity aerobic activity	- Walking at a pace that increases your breathing rate noticeably<br>- Peddling slowly on a stationary bicycle or leisurely cycling<br>- Peddling slowly on an elliptical machine<br>- Rowing slowly on a machine<br>- Slow dancing<br>- Aqua-aerobics
3) Cool-down	5 minutes	Static flexibility OR slowly reduce the pace of aerobic exercise	- Static stretching (10-30 seconds per stretch, 2-4 repetitions of each exercise):<br>• Standing calf stretch<br>• Quadricep stretch<br>• Kneeling hip flexor stretch (iliopsoas)<br>• Seated hamstring stretch<br>• Child's pose stretch<br>• Chest stretch<br>• Cross-body shoulder stretch<br>• Neck lateral flexion stretch

Resistance Exercise Workout Examples at Moderate Intensity Week 3.

Component	Time	Type	Example exercises with machines, free weights, bodyweight, or resistance bands
Resistance Exercise (Day 1)	~30 minutes	Resistance training for all major muscle groups	2-3 sets of 8-12 repetitions (rest 1.5-2 minutes between sets)<br>RPE 12-13; 1-RM: 60-70%<br>• Chest press<br>• Seated row<br>• Leg press<br>• Hip bridges<br>• Leg curls<br>• Arm curls<br>• Abdominal flexions<br>• Planks
Resistance Exercise (Day 2)	~30 minutes	Resistance training for all major muscle groups	2-3 sets of 8-12 repetitions (rest 1.5-2 minutes between sets)<br>RPE 12-13; 1-RM: 60-70%<br>• Shoulder press<br>• Lat pull-downs<br>• Hip kickbacks<br>• Hip abductions<br>• Leg extensions<br>• Triceps extensions<br>• Abdominal twists<br>• Side planks

Neuromotor Exercise Workout Examples at Light to Moderate Intensity Week 3.

Component	Time	Type	Examples
Neuromotor Exercise	20-30 minutes	Training that involves motor skills such as balance, coordination, agility	- Yoga (Hatha sitting/Vinyasa/ Nadisodhana)<br>- Tai chi (qi gong, sitting)<br>- Pilates (traditional)<br>- Balance exercise (15-30 seconds per hold, 5-6 cycles):<br>• two-legged stance<br>• semi-tandem stance<br>• reaching from a narrow stance<br>• tandem walking<br>• standing with eyes closed

Week 4

Aerobic exercise: 3-4 days per week for 30-40 minutes* per day at moderate intensity, to total 125 minutes per week. **Can be accumulated in multiple daily bouts of at least 10 minutes in duration.*

Resistance exercise: 2 days per week for ~30 minutes per session at light to moderate intensity.

Neuromotor exercise: 1 day per week for 20-30 minutes per session at light to moderate intensity.

Flexibility exercise: 2 days per week for ~5 minutes per session*, stretching to the point of tightness or slight discomfort. **Can be implemented into warm-ups or cool-downs.*

Aerobic Exercise Workout Examples at Moderate Intensity Week 4.

Component	Time	Type	Examples
1) Warm-up	5 minutes	Aerobic activity or dynamic stretching	- Walking at a talking pace on a treadmill<br>- Walking to the gym<br>- Dynamic stretching (3-6 sets of 30-90 sec with 15 sec rest periods between sets):<br>• Marching in place<br>• Walking lunges<br>• Hip circles or owners<br>• Arm swings/arm circles<br>• Torso twists
2) Aerobic Exercise	30-40 minutes	Steady state moderate intensity aerobic activity	- Walking at a pace that increases your breathing rate noticeably<br>- Peddling slowly on a stationary bicycle or leisurely cycling<br>- Peddling slowly on an elliptical machine<br>- Rowing slowly on a machine<br>- Slow dancing<br>- Aqua-aerobics
3) Cool-down	5 minutes	Static flexibility OR slowly reduce the pace of aerobic exercise	- Static stretching (10-30 seconds per stretch, 2-4 repetitions of each exercise):<br>• Standing calf stretch<br>• Quadricep stretch<br>• Kneeling hip flexor stretch (iliopsoas)<br>• Seated hamstring stretch<br>• Child's pose stretch<br>• Chest stretch<br>• Cross-body shoulder stretch<br>• Neck lateral flexion stretch

Resistance Exercise Workout Examples at Moderate Intensity Week 4.

Component	Time	Type	Example exercises with machines, free weights, bodyweight, or resistance bands
Resistance Exercise (Day 1)	~30 minutes	Resistance training for all major muscle groups	2-3 sets of 8-12 repetitions (rest 1.5-2 minutes between sets)<br>RPE 12-13; 1-RM: 60-70%<br>• Chest press<br>• Seated row<br>• Leg press<br>• Hip bridges<br>• Leg curls<br>• Arm curls<br>• Abdominal flexions<br>• Planks
Resistance Exercise (Day 2)	~30 minutes	Resistance training for all major muscle groups	2-3 sets of 8-12 repetitions (rest 1.5-2 minutes between sets)<br>RPE 12-13; 1-RM: 60-70%<br>• Shoulder press<br>• Lat pull-downs<br>• Hip kickbacks<br>• Hip abductions<br>• Leg extensions<br>• Triceps extensions<br>• Abdominal twists<br>• Side planks

Neuromotor Exercise Workout Examples at Light to Moderate Intensity Week 4.

Component	Time	Type	Examples
Neuromotor Exercise	20-30 minutes	Training that involves motor skills such as balance, coordination, agility	- Yoga (Hatha sitting/Vinyasa/ Nadisodhana)<br>- Tai chi (qi gong, sitting)<br>- Pilates (traditional)<br>- Balance exercise (15-30 seconds per hold, 5-6 cycles):<br>• two-legged stance<br>• semi-tandem stance<br>• reaching from a narrow stance<br>• tandem walking<br>• standing with eyes closed

Week 5

Aerobic exercise: 3-4 days per week for 30-50 minutes* per day at moderate intensity, to total 150 minutes per week. **Can be accumulated in multiple daily bouts of at least 10 minutes in duration.*

Resistance exercise: 2 days per week for ~30 minutes per session at moderate intensity.

Neuromotor exercise: 2 days per week for 20-30 minutes per session at light to moderate intensity.

Flexibility exercise: 2 days per week for ~5 minutes per session*, stretching to the point of tightness or slight discomfort. **Can be implemented into warm-ups or cool-downs.*

Aerobic Exercise Workout Examples at Moderate Intensity Week 5.

Component	Time	Type	Examples
1) Warm-up	5 minutes	Aerobic activity or dynamic stretching	- Walking at a talking pace on a treadmill<br>- Walking to the gym<br>- Dynamic stretching (3-6 sets of 30-90 sec with 15 sec rest periods between sets):<br>• Marching in place<br>• Walking lunges<br>• Hip circles or owners<br>• Arm swings/arm circles<br>• Torso twists
2) Aerobic Exercise	30-50 minutes	Steady state moderate intensity aerobic activity	- Walking at a pace that increases your breathing rate noticeably<br>- Peddling slowly on a stationary bicycle or leisurely cycling<br>- Peddling slowly on an elliptical machine<br>- Rowing slowly on a machine<br>- Slow dancing<br>- Aqua-aerobics
3) Cool-down	5 minutes	Static flexibility OR slowly reduce the pace of aerobic exercise	- Static stretching (10-30 seconds per stretch, 2-4 repetitions of each exercise):<br>• Standing calf stretch<br>• Quadricep stretch<br>• Kneeling hip flexor stretch (iliopsoas)<br>• Seated hamstring stretch<br>• Child's pose stretch<br>• Chest stretch<br>• Cross-body shoulder stretch<br>• Neck lateral flexion stretch

Resistance Exercise Workout Examples at Moderate Intensity Week 5.

Component	Time	Type	Example exercises with machines, free weights, bodyweight, or resistance bands
Resistance Exercise (Day 1)	~30 minutes	Resistance training for all major muscle groups	2-3 sets of 8-12 repetitions (rest 1.5-2 minutes between sets)<br>RPE 12-13; 1-RM: 60-70%<br>• Chest press<br>• Seated row<br>• Leg press<br>• Hip bridges<br>• Leg curls<br>• Arm curls<br>• Abdominal flexions<br>• Planks
Resistance Exercise (Day 2)	~30 minutes	Resistance training for all major muscle groups	2-3 sets of 8-12 repetitions (rest 1.5-2 minutes between sets)<br>RPE 12-13; 1-RM: 60-70%<br>• Shoulder press<br>• Lat pull-downs<br>• Hip kickbacks<br>• Hip abductions<br>• Leg extensions<br>• Triceps extensions<br>• Abdominal twists<br>• Side planks

Neuromotor Exercise Workout Examples at Light to Moderate Intensity Week 5.

Component	Time	Type	Examples
Neuromotor Exercise	20-30 minutes	Training that involves motor skills such as balance, coordination, agility	- Yoga (Hatha sitting/Vinyasa/ Nadisodhana)<br>- Tai chi (qi gong, sitting)<br>- Pilates (traditional)<br>- Balance exercise (15-30 seconds per hold, 5-6 cycles):<br>• two-legged stance<br>• semi-tandem stance<br>• reaching from a narrow stance<br>• tandem walking<br>• standing with eyes closed

Week 6

Aerobic exercise: 3-4 days per week for 30-50 minutes* per day at moderate intensity, to total 150 minutes per week. **Can be accumulated in multiple daily bouts of at least 10 minutes in duration.*

Resistance exercise: 2 days per week for ~30 minutes per session at light to moderate intensity.

Neuromotor exercise: 2 days per week for 20-30 minutes per session at moderate intensity.

Flexibility exercise: 2 days per week for ~5 minutes per session*, stretching to the point of tightness or slight discomfort. **Can be implemented into warm-ups or cool-downs.*

Aerobic Exercise Workout Examples at Moderate Intensity Week 6.

Component	Time	Type	Examples
1) Warm-up	5 minutes	Aerobic activity or dynamic stretching	- Walking at a talking pace on a treadmill<br>- Walking to the gym<br>- Dynamic stretching (3-6 sets of 30-90 sec with 15 sec rest periods between sets):<br>• Marching in place<br>• Walking lunges<br>• Hip circles or owners<br>• Arm swings/arm circles<br>• Torso twists
2) Aerobic Exercise	30-50 minutes	Steady state moderate intensity aerobic activity	- Walking at a pace that increases your breathing rate noticeably<br>- Peddling slowly on a stationary bicycle or leisurely cycling<br>- Peddling slowly on an elliptical machine<br>- Rowing slowly on a machine<br>- Slow dancing<br>- Aqua-aerobics
3) Cool-down	5 minutes	Static flexibility OR slowly reduce the pace of aerobic exercise	- Static stretching (10-30 seconds per stretch, 2-4 repetitions of each exercise):<br>• Standing calf stretch<br>• Quadricep stretch<br>• Kneeling hip flexor stretch (iliopsoas)<br>• Seated hamstring stretch<br>• Child's pose stretch<br>• Chest stretch<br>• Cross-body shoulder stretch<br>• Neck lateral flexion stretch

Resistance Exercise Workout Examples at Moderate Intensity Week 6.

Component	Time	Type	Example exercises with machines, free weights, bodyweight, or resistance bands
Resistance Exercise (Day 1)	~30 minutes	Resistance training for all major muscle groups	2-3 sets of 8-12 repetitions (rest 1.5-2 minutes between sets)<br>RPE 12-13; 1-RM: 60-70%<br>• Chest press<br>• Seated row<br>• Leg press<br>• Hip bridges<br>• Leg curls<br>• Arm curls<br>• Abdominal flexions<br>• Planks
Resistance Exercise (Day 2)	~30 minutes	Resistance training for all major muscle groups	2-3 sets of 8-12 repetitions (rest 1.5-2 minutes between sets)<br>RPE 12-13; 1-RM: 60-70%<br>• Shoulder press<br>• Lat pull-downs<br>• Hip kickbacks<br>• Hip abductions<br>• Leg extensions<br>• Triceps extensions<br>• Abdominal twists<br>• Side planks

Neuromotor Exercise Workout Examples at Light to Moderate Intensity Week 6.

Component	Time	Type	Examples
Neuromotor Exercise	20-30 minutes	Training that involves motor skills such as balance, coordination, agility	- Yoga (Hatha sitting/Vinyasa/ Nadisodhana)<br>- Tai chi (qi gong, sitting)<br>- Pilates (traditional)<br>- Balance exercise (15-30 seconds per hold, 5-6 cycles):<br>• two-legged stance<br>• semi-tandem stance<br>• reaching from a narrow stance<br>• tandem walking<br>• standing with eyes closed

Week 7

Aerobic exercise: 4-5 days per week for 30-50 minutes* per day at moderate intensity or 15-25 minutes* per day at vigorous intensity (or a combination), to total 200 minutes per week. **Can be accumulated in multiple daily bouts of at least 10 minutes in duration.*

Resistance exercise: 2-3 days per week for ~45 minutes per session at moderate to vigorous intensity.

Neuromotor exercise: 2 days per week for 20-30 minutes per session at moderate intensity.

Flexibility exercise: ≥2-3 days per week for ~5 minutes per session*, stretching to the point of tightness or slight discomfort. **Can be implemented into warm-ups or cool-downs.*

Aerobic Exercise Workout Examples at Moderate Intensity Week 7.

Component	Time	Type	Examples
1) Warm-up	5 minutes	Aerobic activity or dynamic stretching	- Walking at a talking pace on a treadmill<br>- Walking to the gym<br>- Dynamic stretching (3-6 sets of 30-90 sec with 15 sec rest periods between sets):<br>• Marching in place<br>• Walking lunges<br>• Hip circles or owners<br>• Arm swings/arm circles<br>• Torso twists
2) Aerobic Exercise	30-50 minutes<br><br>OR<br><br>15-25 minutes	Steady state moderate intensity aerobic activity<br><br>OR<br><br>Steady state vigorous intensity aerobic activity or high intensity interval training	- Walking at a pace that increases your breathing rate noticeably<br>- Peddling slowly on a stationary bicycle or leisurely cycling<br>- Peddling slowly on an elliptical machine<br>- Rowing slowly on a machine<br>- Slow dancing<br>- Aqua-aerobics<br><br>- Walking very briskly, jogging, or running<br>- Peddling fast on a stationary bicycle or cycling fast or uphill<br>- Peddling fast on an elliptical machine<br>- Rowing fast on a machine<br>- Stepping on the stair stepper machine<br>- Fast dancing<br>- Aerobics
3) Cool-down	5 minutes	Static flexibility OR slowly reduce the pace of aerobic exercise	- Static stretching (10-30 seconds per stretch, 2-4 repetitions of each exercise):<br>• Standing calf stretch<br>• Quadricep stretch<br>• Kneeling hip flexor stretch (iliopsoas)<br>• Seated hamstring stretch

			• Child's pose stretch<br>• Chest stretch<br>• Cross-body shoulder stretch<br>• Neck lateral flexion stretch

Resistance Exercise Workout Examples at Moderate to Vigorous Intensity Week 7.

Component	Time	Type	Example exercises with machines, free weights, bodyweight, or resistance bands
Resistance Exercise (Day 1)	~45 minutes	Resistance training for all major muscle groups	3-4 sets of 8-12 repetitions (rest 1.5-2.5 minutes between sets)<br>RPE 12-13 to 14-17; 1-RM: 60-80%<br>• Chest press<br>• Seated row<br>• Leg press<br>• Hip bridges<br>• Leg curls<br>• Arm curls<br>• Abdominal flexions<br>• Planks
Resistance Exercise (Day 2)	~45 minutes	Resistance training for all major muscle groups	3-4 sets of 8-12 repetitions (rest 1.5-2.5 minutes between sets)<br>RPE 12-13 to 14-17; 1-RM: 60-80%<br>• Shoulder press<br>• Lat pull-downs<br>• Hip kickbacks<br>• Hip abductions<br>• Leg extensions<br>• Triceps extensions<br>• Abdominal twists<br>• Side planks
Resistance Exercise (Day 3)	~45 minutes	Resistance training for all major muscle groups	3-4 sets of 8-12 repetitions (rest 1.5-2.5 minutes between sets)<br>RPE 12-13 to 14-17; 1-RM: 60-80%<br>• Pick 6-10 exercises from above to train major muscle groups

Neuromotor Exercise Workout Examples at Moderate Intensity Week 7.

Component	Time	Type	Examples
Neuromotor Exercise	20-30 minutes	Training that involves motor skills such as balance, coordination, agility	- Yoga (power/Surya Namaskar)<br>- Tai chi (qi gong standing/yang style)<br>- Pilates (POUND® with drumming)<br>- Single leg or Bosu ball exercises<br>- Balance exercises (15-30 seconds per hold, 5-6 cycles):<br>• tandem stance<br>• one-legged stance<br>• stepping over obstacles<br>• heel or toe walks<br>• walking while turning the head

Week 8

Aerobic exercise: 4-5 days per week for 30-50 minutes* per day at moderate intensity or 15-25 minutes* per day at vigorous intensity (or a combination), to total 200 minutes per week. **Can be accumulated in multiple daily bouts of at least 10 minutes in duration.*

Resistance exercise: 2-3 days per week for ~45 minutes per session at moderate to vigorous intensity.

Neuromotor exercise: 2 days per week for 20-30 minutes per session at moderate intensity.

Flexibility exercise: ≥2-3 days per week for ~5 minutes per session*, stretching to the point of tightness or slight discomfort. **Can be implemented into warm-ups or cool-downs.*

Aerobic Exercise Workout Examples at Moderate Intensity Week 8.

Component	Time	Type	Examples
1) Warm-up	5 minutes	Aerobic activity or dynamic stretching	- Walking at a talking pace on a treadmill<br>- Walking to the gym<br>- Dynamic stretching (3-6 sets of 30-90 sec with 15 sec rest periods between sets):<br>• Marching in place<br>• Walking lunges<br>• Hip circles or owners<br>• Arm swings/arm circles<br>• Torso twists
2) Aerobic Exercise	30-50 minutes<br><br>OR<br><br>15-25 minutes	Steady state moderate intensity aerobic activity<br><br>OR<br><br>Steady state vigorous intensity aerobic activity or high intensity interval training	- Walking at a pace that increases your breathing rate noticeably<br>- Peddling slowly on a stationary bicycle or leisurely cycling<br>- Peddling slowly on an elliptical machine<br>- Rowing slowly on a machine<br>- Slow dancing<br>- Aqua-aerobics<br><br>- Walking very briskly, jogging, or running<br>- Peddling fast on a stationary bicycle or cycling fast or uphill<br>- Peddling fast on an elliptical machine<br>- Rowing fast on a machine<br>- Stepping on the stair stepper machine<br>- Fast dancing<br>- Aerobics
3) Cool-down	5 minutes	Static flexibility OR slowly reduce the pace of aerobic exercise	- Static stretching (10-30 seconds per stretch, 2-4 repetitions of each exercise):<br>• Standing calf stretch<br>• Quadricep stretch<br>• Kneeling hip flexor stretch (iliopsoas)<br>• Seated hamstring stretch

			• Child's pose stretch<br>• Chest stretch<br>• Cross-body shoulder stretch<br>• Neck lateral flexion stretch

Resistance Exercise Workout Examples at Moderate to Vigorous Intensity Week 8.

Component	Time	Type	Example exercises with machines, free weights, bodyweight, or resistance bands
Resistance Exercise (Day 1)	~45 minutes	Resistance training for all major muscle groups	3-4 sets of 8-12 repetitions (rest 1.5-2.5 minutes between sets)<br>RPE 12-13 to 14-17; 1-RM: 60-80%<br>• Chest press<br>• Seated row<br>• Leg press<br>• Hip bridges<br>• Leg curls<br>• Arm curls<br>• Abdominal flexions<br>• Planks
Resistance Exercise (Day 2)	~45 minutes	Resistance training for all major muscle groups	3-4 sets of 8-12 repetitions (rest 1.5-2.5 minutes between sets)<br>RPE 12-13 to 14-17; 1-RM: 60-80%<br>• Shoulder press<br>• Lat pull-downs<br>• Hip kickbacks<br>• Hip abductions<br>• Leg extensions<br>• Triceps extensions<br>• Abdominal twists<br>• Side planks
Resistance Exercise (Day 3)	~45 minutes	Resistance training for all major muscle groups	3-4 sets of 8-12 repetitions (rest 1.5-2.5 minutes between sets)<br>RPE 12-13 to 14-17; 1-RM: 60-80%<br>• Pick 6-10 exercises from above to train major muscle groups

Neuromotor Exercise Workout Examples at Moderate Intensity Week 8.

Component	Time	Type	Examples
Neuromotor Exercise	20-30 minutes	Training that involves motor skills such as balance, coordination, agility	- Yoga (power/Surya Namaskar)<br>- Tai chi (qi gong standing/yang style)<br>- Pilates (POUND® with drumming)<br>- Single leg or Bosu ball exercises<br>- Balance exercises (15-30 seconds per hold, 5-6 cycles):<br>• tandem stance<br>• one-legged stance<br>• stepping over obstacles<br>• heel or toe walks<br>• walking while turning the head

Week 9

Aerobic exercise: 4-5 days per week for 30-50 minutes* per day at moderate intensity or 15-25 minutes* per day at vigorous intensity (or a combination), to total 200 minutes per week. **Can be accumulated in multiple daily bouts of at least 10 minutes in duration.*

Resistance exercise: 2-3 days per week for ~45 minutes per session at moderate to vigorous intensity.

Neuromotor exercise: 2 days per week for 20-30 minutes per session at moderate intensity.

Flexibility exercise: ≥2-3 days per week for ~5 minutes per session*, stretching to the point of tightness or slight discomfort. **Can be implemented into warm-ups or cool-downs.*

Aerobic Exercise Workout Examples at Moderate Intensity Week 9.

Component	Time	Type	Examples
1) Warm-up	5 minutes	Aerobic activity or dynamic stretching	- Walking at a talking pace on a treadmill<br>- Walking to the gym<br>- Dynamic stretching (3-6 sets of 30-90 sec with 15 sec rest periods between sets):<br>• Marching in place<br>• Walking lunges<br>• Hip circles or owners<br>• Arm swings/arm circles<br>• Torso twists
2) Aerobic Exercise	30-50 minutes<br><br>OR<br><br>15-25 minutes	Steady state moderate intensity aerobic activity<br><br>OR<br><br>Steady state vigorous intensity aerobic activity or high intensity interval training	- Walking at a pace that increases your breathing rate noticeably<br>- Peddling slowly on a stationary bicycle or leisurely cycling<br>- Peddling slowly on an elliptical machine<br>- Rowing slowly on a machine<br>- Slow dancing<br>- Aqua-aerobics<br><br>- Walking very briskly, jogging, or running<br>- Peddling fast on a stationary bicycle or cycling fast or uphill<br>- Peddling fast on an elliptical machine<br>- Rowing fast on a machine<br>- Stepping on the stair stepper machine<br>- Fast dancing<br>- Aerobics
3) Cool-down	5 minutes	Static flexibility OR slowly reduce the pace of aerobic exercise	- Static stretching (10-30 seconds per stretch, 2-4 repetitions of each exercise):<br>• Standing calf stretch<br>• Quadricep stretch<br>• Kneeling hip flexor stretch (iliopsoas)<br>• Seated hamstring stretch

			• Child's pose stretch<br>• Chest stretch<br>• Cross-body shoulder stretch<br>• Neck lateral flexion stretch

Resistance Exercise Workout Examples at Moderate to Vigorous Intensity Week 9.

Component	Time	Type	Example exercises with machines, free weights, bodyweight, or resistance bands
Resistance Exercise (Day 1)	~45 minutes	Resistance training for all major muscle groups	3-4 sets of 8-12 repetitions (rest 1.5-2.5 minutes between sets)<br>RPE 12-13 to 14-17; 1-RM: 60-80%<br>• Chest press<br>• Seated row<br>• Leg press<br>• Hip bridges<br>• Leg curls<br>• Arm curls<br>• Abdominal flexions<br>• Planks
Resistance Exercise (Day 2)	~45 minutes	Resistance training for all major muscle groups	3-4 sets of 8-12 repetitions (rest 1.5-2.5 minutes between sets)<br>RPE 12-13 to 14-17; 1-RM: 60-80%<br>• Shoulder press<br>• Lat pull-downs<br>• Hip kickbacks<br>• Hip abductions<br>• Leg extensions<br>• Triceps extensions<br>• Abdominal twists<br>• Side planks
Resistance Exercise (Day 3)	~45 minutes	Resistance training for all major muscle groups	3-4 sets of 8-12 repetitions (rest 1.5-2.5 minutes between sets)<br>RPE 12-13 to 14-17; 1-RM: 60-80%<br>• Pick 6-10 exercises from above to train major muscle groups

Neuromotor Exercise Workout Examples at Moderate Intensity Week 9.

Component	Time	Type	Examples
Neuromotor Exercise	20-30 minutes	Training that involves motor skills such as balance, coordination, agility	- Yoga (power/Surya Namaskar)<br>- Tai chi (qi gong standing/yang style)<br>- Pilates (POUND® with drumming)<br>- Single leg or Bosu ball exercises<br>- Balance exercises (15-30 seconds per hold, 5-6 cycles):<br>• tandem stance<br>• one-legged stance<br>• stepping over obstacles<br>• heel or toe walks<br>• walking while turning the head

Week 10

Aerobic exercise: 5-6 days per week for 30-60 minutes* per day at moderate intensity or 15-30 minutes* per day at vigorous intensity (or a combination), to total 250-300 minutes per week. **Can be accumulated in multiple daily bouts of at least 10 minutes in duration.*

Resistance exercise: 2-3 days per week for ~45 minutes per session at moderate to vigorous intensity.

Neuromotor exercise: 2 days per week for 20-30 minutes per session at moderate intensity.

Flexibility exercise: ≥2-3 days per week for ~5 minutes per session*, stretching to the point of tightness or slight discomfort. **Can be implemented into warm-ups or cool-downs.*

Aerobic Exercise Workout Examples at Moderate Intensity Week 10.

Component	Time	Type	Examples
1) Warm-up	5 minutes	Aerobic activity or dynamic stretching	- Walking at a talking pace on a treadmill<br>- Walking to the gym<br>- Dynamic stretching (3-6 sets of 30-90 sec with 15 sec rest periods between sets):<br>• Marching in place<br>• Walking lunges<br>• Hip circles or owners<br>• Arm swings/arm circles<br>• Torso twists
2) Aerobic Exercise	30-60 minutes<br><br>OR<br><br>15-30 minutes	Steady state moderate intensity aerobic activity<br><br>OR<br><br>Steady state vigorous intensity aerobic activity or high intensity interval training	- Walking at a pace that increases your breathing rate noticeably<br>- Peddling slowly on a stationary bicycle or leisurely cycling<br>- Peddling slowly on an elliptical machine<br>- Rowing slowly on a machine<br>- Slow dancing<br>- Aqua-aerobics<br><br>- Walking very briskly, jogging, or running<br>- Peddling fast on a stationary bicycle or cycling fast or uphill<br>- Peddling fast on an elliptical machine<br>- Rowing fast on a machine<br>- Stepping on the stair stepper machine<br>- Fast dancing<br>- Aerobics
3) Cool-down	5 minutes	Static flexibility OR slowly reduce the pace of aerobic exercise	- Static stretching (10-30 seconds per stretch, 2-4 repetitions of each exercise):<br>• Standing calf stretch<br>• Quadricep stretch<br>• Kneeling hip flexor stretch (iliopsoas)<br>• Seated hamstring stretch

			• Child's pose stretch<br>• Chest stretch<br>• Cross-body shoulder stretch<br>• Neck lateral flexion stretch

Resistance Exercise Workout Examples at Moderate to Vigorous Intensity Week 10.

Component	Time	Type	Example exercises with machines, free weights, bodyweight, or resistance bands
Resistance Exercise (Day 1)	~45 minutes	Resistance training for all major muscle groups	3-4 sets of 8-12 repetitions (rest 1.5-2.5 minutes between sets)<br>RPE 12-13 to 14-17; 1-RM: 60-80%<br>• Chest press<br>• Seated row<br>• Leg press<br>• Hip bridges<br>• Leg curls<br>• Arm curls<br>• Abdominal flexions<br>• Planks
Resistance Exercise (Day 2)	~45 minutes	Resistance training for all major muscle groups	3-4 sets of 8-12 repetitions (rest 1.5-2.5 minutes between sets)<br>RPE 12-13 to 14-17; 1-RM: 60-80%<br>• Shoulder press<br>• Lat pull-downs<br>• Hip kickbacks<br>• Hip abductions<br>• Leg extensions<br>• Triceps extensions<br>• Abdominal twists<br>• Side planks
Resistance Exercise (Day 3)	~45 minutes	Resistance training for all major muscle groups	3-4 sets of 8-12 repetitions (rest 1.5-2.5 minutes between sets)<br>RPE 12-13 to 14-17; 1-RM: 60-80%<br>• Pick 6-10 exercises from above to train major muscle groups

Neuromotor Exercise Workout Examples at Moderate Intensity Week 10.

Component	Time	Type	Examples
Neuromotor Exercise	20-30 minutes	Training that involves motor skills such as balance, coordination, agility	- Yoga (power/Surya Namaskar)<br>- Tai chi (qi gong standing/yang style)<br>- Pilates (POUND® with drumming)<br>- Single leg or Bosu ball exercises<br>- Balance exercises (15-30 seconds per hold, 5-6 cycles):<br>• tandem stance<br>• one-legged stance<br>• stepping over obstacles<br>• heel or toe walks<br>• walking while turning the head

Week 11

Aerobic exercise: 5-6 days per week for 30-60 minutes* per day at moderate intensity or 15-30 minutes* per day at vigorous intensity (or a combination), to total 250-300 minutes per week. **Can be accumulated in multiple daily bouts of at least 10 minutes in duration.*

Resistance exercise: 2-3 days per week for ~45 minutes per session at moderate to vigorous intensity.

Neuromotor exercise: 2 days per week for 20-30 minutes per session at moderate intensity.

Flexibility exercise: ≥2-3 days per week for ~5 minutes per session*, stretching to the point of tightness or slight discomfort. **Can be implemented into warm-ups or cool-downs.*

Aerobic Exercise Workout Examples at Moderate Intensity Week 11.

Component	Time	Type	Examples
1) Warm-up	5 minutes	Aerobic activity or dynamic stretching	- Walking at a talking pace on a treadmill<br>- Walking to the gym<br>- Dynamic stretching (3-6 sets of 30-90 sec with 15 sec rest periods between sets):<br>• Marching in place<br>• Walking lunges<br>• Hip circles or owners<br>• Arm swings/arm circles<br>• Torso twists
2) Aerobic Exercise	30-60 minutes<br><br>OR<br><br>15-30 minutes	Steady state moderate intensity aerobic activity<br><br>OR<br><br>Steady state vigorous intensity aerobic activity or high intensity interval training	- Walking at a pace that increases your breathing rate noticeably<br>- Peddling slowly on a stationary bicycle or leisurely cycling<br>- Peddling slowly on an elliptical machine<br>- Rowing slowly on a machine<br>- Slow dancing<br>- Aqua-aerobics<br><br>- Walking very briskly, jogging, or running<br>- Peddling fast on a stationary bicycle or cycling fast or uphill<br>- Peddling fast on an elliptical machine<br>- Rowing fast on a machine<br>- Stepping on the stair stepper machine<br>- Fast dancing<br>- Aerobics
3) Cool-down	5 minutes	Static flexibility OR slowly reduce the pace of aerobic exercise	- Static stretching (10-30 seconds per stretch, 2-4 repetitions of each exercise):<br>• Standing calf stretch<br>• Quadricep stretch<br>• Kneeling hip flexor stretch (iliopsoas)<br>• Seated hamstring stretch

			• Child's pose stretch<br>• Chest stretch<br>• Cross-body shoulder stretch<br>• Neck lateral flexion stretch

Resistance Exercise Workout Examples at Moderate to Vigorous Intensity Week 11.

Component	Time	Type	Example exercises with machines, free weights, bodyweight, or resistance bands
Resistance Exercise (Day 1)	~45 minutes	Resistance training for all major muscle groups	3-4 sets of 8-12 repetitions (rest 1.5-2.5 minutes between sets)<br>RPE 12-13 to 14-17; 1-RM: 60-80%<br>• Chest press<br>• Seated row<br>• Leg press<br>• Hip bridges<br>• Leg curls<br>• Arm curls<br>• Abdominal flexions<br>• Planks
Resistance Exercise (Day 2)	~45 minutes	Resistance training for all major muscle groups	3-4 sets of 8-12 repetitions (rest 1.5-2.5 minutes between sets)<br>RPE 12-13 to 14-17; 1-RM: 60-80%<br>• Shoulder press<br>• Lat pull-downs<br>• Hip kickbacks<br>• Hip abductions<br>• Leg extensions<br>• Triceps extensions<br>• Abdominal twists<br>• Side planks
Resistance Exercise (Day 3)	~45 minutes	Resistance training for all major muscle groups	3-4 sets of 8-12 repetitions (rest 1.5-2.5 minutes between sets)<br>RPE 12-13 to 14-17; 1-RM: 60-80%<br>• Pick 6-10 exercises from above to train major muscle groups

Neuromotor Exercise Workout Examples at Moderate Intensity Week 11.

Component	Time	Type	Examples
Neuromotor Exercise	20-30 minutes	Training that involves motor skills such as balance, coordination, agility	- Yoga (power/Surya Namaskar)<br>- Tai chi (qi gong standing/yang style)<br>- Pilates (POUND® with drumming)<br>- Single leg or Bosu ball exercises<br>- Balance exercises (15-30 seconds per hold, 5-6 cycles):<br>• tandem stance<br>• one-legged stance<br>• stepping over obstacles<br>• heel or toe walks<br>• walking while turning the head

Week 12

Aerobic exercise: 5-6 days per week for 30-60 minutes* per day at moderate intensity or 15-30 minutes* per day at vigorous intensity (or a combination), to total 250-300 minutes per week. **Can be accumulated in multiple daily bouts of at least 10 minutes in duration.*

Resistance exercise: 2-3 days per week for ~45 minutes per session at moderate to vigorous intensity.

Neuromotor exercise: 2 days per week for 20-30 minutes per session at moderate intensity.

Flexibility exercise: ≥2-3 days per week for ~5 minutes per session*, stretching to the point of tightness or slight discomfort. **Can be implemented into warm-ups or cool-downs.*

Aerobic Exercise Workout Examples at Moderate Intensity Week 12.

Component	Time	Type	Examples
1) Warm-up	5 minutes	Aerobic activity or dynamic stretching	- Walking at a talking pace on a treadmill<br>- Walking to the gym<br>- Dynamic stretching (3-6 sets of 30-90 sec with 15 sec rest periods between sets):<br>• Marching in place<br>• Walking lunges<br>• Hip circles or owners<br>• Arm swings/arm circles<br>• Torso twists
2) Aerobic Exercise	30-60 minutes<br><br>OR<br><br>15-30 minutes	Steady state moderate intensity aerobic activity<br><br>OR<br><br>Steady state vigorous intensity aerobic activity or high intensity interval training	- Walking at a pace that increases your breathing rate noticeably<br>- Peddling slowly on a stationary bicycle or leisurely cycling<br>- Peddling slowly on an elliptical machine<br>- Rowing slowly on a machine<br>- Slow dancing<br>- Aqua-aerobics<br><br>- Walking very briskly, jogging, or running<br>- Peddling fast on a stationary bicycle or cycling fast or uphill<br>- Peddling fast on an elliptical machine<br>- Rowing fast on a machine<br>- Stepping on the stair stepper machine<br>- Fast dancing<br>- Aerobics
3) Cool-down	5 minutes	Static flexibility OR slowly reduce the pace of aerobic exercise	- Static stretching (10-30 seconds per stretch, 2-4 repetitions of each exercise):<br>• Standing calf stretch<br>• Quadricep stretch<br>• Kneeling hip flexor stretch (iliopsoas)<br>• Seated hamstring stretch

			• Child's pose stretch<br>• Chest stretch<br>• Cross-body shoulder stretch<br>• Neck lateral flexion stretch

Resistance Exercise Workout Examples at Moderate to Vigorous Intensity Week 12.

Component	Time	Type	Example exercises with machines, free weights, bodyweight, or resistance bands
Resistance Exercise (Day 1)	~45 minutes	Resistance training for all major muscle groups	3-4 sets of 8-12 repetitions (rest 1.5-2.5 minutes between sets)<br>RPE 12-13 to 14-17; 1-RM: 60-80%<br>• Chest press<br>• Seated row<br>• Leg press<br>• Hip bridges<br>• Leg curls<br>• Arm curls<br>• Abdominal flexions<br>• Planks
Resistance Exercise (Day 2)	~45 minutes	Resistance training for all major muscle groups	3-4 sets of 8-12 repetitions (rest 1.5-2.5 minutes between sets)<br>RPE 12-13 to 14-17; 1-RM: 60-80%<br>• Shoulder press<br>• Lat pull-downs<br>• Hip kickbacks<br>• Hip abductions<br>• Leg extensions<br>• Triceps extensions<br>• Abdominal twists<br>• Side planks
Resistance Exercise (Day 3)	~45 minutes	Resistance training for all major muscle groups	3-4 sets of 8-12 repetitions (rest 1.5-2.5 minutes between sets)<br>RPE 12-13 to 14-17; 1-RM: 60-80%<br>• Pick 6-10 exercises from above to train major muscle groups

Neuromotor Exercise Workout Examples at Moderate Intensity Week 12.

Component	Time	Type	Examples
Neuromotor Exercise	20-30 minutes	Training that involves motor skills such as balance, coordination, agility	- Yoga (power/Surya Namaskar)<br>- Tai chi (qi gong standing/yang style)<br>- Pilates (POUND® with drumming)<br>- Single leg or Bosu ball exercises<br>- Balance exercises (15-30 seconds per hold, 5-6 cycles):<br>• tandem stance<br>• one-legged stance<br>• stepping over obstacles<br>• heel or toe walks<br>• walking while turning the head

Participant 12-Week Exercise Program Information Packet

Participant ID:

Date Provided:

12-Week Start Date:

12-Week End Date:

Principle Investigators:

Dr. Antonio Fernandez, MD, FACC, FAHA
Medical Director of Preventive Cardiology, Hartford Hospital

Dr. Linda Pescatello, PhD, FACSM
Board of Trustees Distinguished Professor of Kinesiology, University of Connecticut

Dr. Peter Robinson, MD
Assistant Professor of Cardiology, UConn Health

UConn Graduate Research Assistant:

Alexander Wright, MS
Email: Alexander.Wright@hhchealth.org
Phone: (860) 486-6814

This research is approved by the Hartford HealthCare Institutional Review Board, with reliance agreements at UConn Storrs and UConn Health.

Contents Page

Exercise Program for Hypertension 3
Special Considerations 4
Exercise Type Definitions 5
Exercise Type Examples 6
Exercise Intensity Definitions 8
How to Take Your Pulse 9
Self-Monitor Your Physical Activity 9
Guidelines for Exercise Progression 10
Contact Details for the UConn Graduate Research Assistant 10
12 Week Exercise Program Progression Guidance 11
Week 1 12
Week 2 14
Week 3 16
Week 4 18
Week 5 20
Week 6 22
Week 7 24
Week 8 27
Week 9 30
Week 10 33
Week 11 36
Week 12 39

Exercise Program for Hypertension

FITT	Aerobic* and/or Resistance*		Neuromotor**	Flexibility
Frequency	≥2-3 days per week	≥2-3 days per week	≥2-3 days per week	≥2-3 days per week with daily being most effective
Intensity	**Moderate intensity:** you can talk comfortably but not sing<br>64-76% HRmax; 40% - 59% VO_2R or HRR; RPE 12-13<br>to **Vigorous intensity***:** you cannot say more than 5 words without grasping for breath<br>77-95% HRmax; 60% - 80% VO_2R or HRR; RPE 14-17	**Moderate intensity:** i.e., 60% - 70% 1-RM for 12-18 repetitions<br>May progress to **vigorous intensity:** 80% 1-RM for 8-12 repetitions<br>For novice exercisers begin with 40-50% 1-RM	Light to moderate intensity	Stretch to the point of feeling tightness or slight discomfort
Time	≥20-30 min per day to total ≥90-150 min per week of continuous or accumulated exercise of any duration	2-4 sets of 8-12 repetitions of 8-10 resistance exercises of each of the major muscle groups per session to total ≥20 min per session with rest days interspersed depending on the muscle groups being exercised	≥20-30 min per session	Hold static stretch for 10-30 sec with 2-4 repetitions of each exercise targeting the major muscle tendon units to total 60 sec of total stretching time for each exercise; ≥10 min per session
Type	Prolonged, rhythmic activities using large muscle groups (e.g., walking, cycling, swimming)	Resistance machines, free weights, resistance bands, and/or functional body weight exercise	Mind-Body exercise involving motor skills and/or functional body weight and flexibility exercise such as yoga, pilates, and tai chi	Static, dynamic, and/or proprioceptive neuromuscular facilitation
FITT = Frequency, Intensity, Time and Type; 1-RM = 1 Repetition Maximum; RPE = Rating of Perceived Exertion (Borg 6-20 scale); HRmax = Heart Rate Maximum; HRR = Heart Rate Reserve; VO_2R = Oxygen Uptake Reserve; PNF = Proprioceptive Neuromuscular Facilitation.				

*Engage in aerobic and/or resistance exercise, alone or combined, on most, preferably all days of the week. The frequency recommendation is made due to the immediate blood pressure lowering effects of exercise, termed *postexercise hypotension.*

**Neuromotor functional body weight exercise can be substituted for resistance exercise, and depending on the amount of flexibility exercise integrated into a session, neuromotor flexibility exercise can be substituted for flexibility exercise depending on patient/client preference.

***The magnitude of the blood pressure reductions resulting from aerobic exercise are directly proportional to intensity such that the greatest BP reductions occur after vigorous intensity if the patient/client is willing and able to perform vigorous intensity exercise.

Reference: American College of Sports Medicine, Ozemek C, Bonikowske AR, Christle JW, Gallo PM, eds. ACSM's Guidelines for Exercise Testing and Prescription. 12th Ed, p.365. Wolters Kluwer; 2026.

Special Considerations

Medication Considerations: Yes/No

If YES, list medication(s) taken and dose:

If YES, list potential side effects of medication as related to exercise.

If YES, list special considerations for the medication as related to exercise.

When you perform resistance exercise, do not take a deep breath when you lift a weight. Holding your breath can make your blood pressure rise very high and cause dizziness or fainting.

Exercise Type Definitions

Aerobic Exercise: Continuous exercise involving large muscle groups such as walking, running, riding a bicycle, cardio machines and rowing.

Steady State Aerobic Exercise: Maintaining exercise at light to moderate intensity.

High Intensity Interval Training: Performing bouts of higher intensity exercise separated by rest.

Resistance Exercise: Exercises that use opposing forces to strengthen or develop muscles such as weight training with free weights, resistance machines, or using resistance bands or body weight.

Neuromotor Exercise: Exercises that combine balance, coordination, and agility such as yoga, tai chi, and Pilates.

Flexibility: Movements that improve the range of motion of a joint.

Dynamic Flexibility: Slow moving stretches that increase reach and range of motion as the movement is repeated. Examples: Leg swings, arm swings, torso twists.

Static Flexibility: Slowly stretching a muscle/tendon group and holding the position for 10-30 sec. Examples: Pike stretch, glute stretch, quad stretch

Proprioceptive Neuromuscular Facilitation (PNF): Applying force with a muscle against an opposing force followed by performing a static stretch of the muscle.

Concurrent Exercise: Performing aerobic and resistance exercise in the same session or near one another.

Major Muscle Groups: Shoulders, chest, back, hamstrings, quadriceps, calves, biceps, triceps, core.

Exercise Type Examples

Aerobic Exercise

Light Intensity (RPE 8-11)	Moderate Intensity (RPE 12-13)	Vigorous Intensity (RPE 14-20)
Walking slowly, leisurely	Walking the dog or walking outside	Brisk walking or jogging
Cycling slowly with a pedal desk	Cycling at a self-selected comfortable pace	Cycling at a moderate pace
Group class - Vinyasa yoga, tai chi (qi gong), or stretching	Group class – Power yoga or tai chi (yang style)	Group class - Zumba or fast ballroom dancing
Cleaning, sweeping, or washing dishes slowly	Water aerobics	Swimming laps, freestyle
Pickleball and Tennis, serving practice	Pickleball and Tennis, doubles	Pickleball and Tennis, singles

Resistance Exercise

Light Intensity (RPE 8-11)	Moderate Intensity (RPE 12-13)	Vigorous Intensity (RPE 14-20)
Yard work at a slow pace	Gardening - watering, weeding, planting	Gardening - using heavy tools, digging or filling garden
Canoeing at a slow pace	Kayaking at a self-selected comfortable pace	Rowing on a stationary ergometer
Group class - Vinyasa yoga, tai chi (qi gong)	Group class – Power yoga or tai chi (yang style)	Group class - circuit training with minimal rest
Bodyweight exercises (curl-ups, planks)	Bodyweight exercises (push-ups, lunges)	Bodyweight exercises (jumping jacks, burpees)
Lifting weights <50% of one repetition maximum (being able to perform 15-20 reps at this weight)	Lifting weights 50-69% of one repetition maximum (being able to perform 12-18 reps at this weight)	Lifting weights 70 to 85% of one repetition maximum (being able to perform 8-12 reps at this weight)

Neuromotor Exercise

**Light to Moderate Intensity (RPE 8-13)**	**Moderate Intensity (RPE 12-13)**
Yoga (Hatha sitting/Vinyasa/ Nadisodhana)	Yoga (power/Surya Namaskar)
Tai chi (qi gong, sitting)	Tai chi (qi gong standing/yang style)
Pilates (traditional)	Pilates (POUND® with drumming)
Balance exercises (beginner)	Balance exercises (general)
Slow dancing	Functional bodyweight exercises

Exercise Intensity Definitions

Adapted Version of Borg 6-20 Rating of Perceived Exertion (RPE) Scale with Exercise Intensity Differentiation:

Borg 6-20 RPE	Intensity	Intensity Definition
6	No exertion	
7	Very Light	
8		
9	Light	A level of physical exertion that causes slight increases in heart rate and breathing (i.e., warm up with dynamic flexibility, cool down with slow aerobic movements and static stretching). ***Use the talk test:*** *Light intensity should make your breathing slightly increase, but you can still talk and sing easily.*<br>Aerobic exercise: <64% HRmax, <40% VO_2R or HRR
10		
11		
12	Moderate	A level of physical exertion that causes increases in heart rate and breathing (i.e., brisk walking, weight training at an intensity that is not hard, but takes effort). ***Use the talk test:*** *Moderate intensity should make your breathing rate increase noticeably. You can still talk but not sing.*<br>Aerobic exercise: 64-76% HRmax, 40-59% VO_2R or HRR.<br>Resistance exercise: 50-69% of 1-RM intensity of 12-18 reps.
13		
14	Vigorous	A level of physical exertion that causes substantial increases in heart rate and breathing (i.e., running, weight training until fatigue). ***Use the talk test:*** *Vigorous intensity should make you breathe hard enough that you can only say a few words before you have to take a breath and you can't sing.*<br>Aerobic exercise: 77-95% HRmax, ≥ 60% VO_2R or HRR<br>Resistance exercise: 70-85% of 1-RM intensity of 8-12 reps.
15		
16		
17		
18	Near Maximal to Maximal	
19		
20		

HRmax = Heart Rate Maximum. The HRmax is the maximum heart rate you can achieve during exercise. HRmax declines with age.

HRR = Heart Rate Reserve. The percentage of HRR is calculated as: (the heart rate during exercise – the heart rate at rest) ÷ (the maximum heart rate during exercise – the heart rate at rest) × 100%.

VO_2R = Oxygen Uptake Reserve. The percentage of VO_2R is calculated as: (the rate of oxygen consumption during exercise – the rate of oxygen consumption at rest) ÷ (the maximum rate of oxygen consumption during exercise – the rate of oxygen consumption at rest) × 100%.

1-RM = 1 Repetition Maximum. The 1-RM is the maximum weight lifted for a single repetition for a given exercise.

How to Take Your Pulse

1) You can use a technique called pulse palpitation, which involves "feeling" the pulse.
2) Place your index finger and middle fingers over the radial artery, located near the thump side of the wrist.
3) Count the pulse for 30-60 seconds. The 30-second count is multiplied by 2 to determine the 1-minute resting heart rate in beats per minute (bpm).

Self-Monitor Your Physical Activity

1) The *Timeline Followback for Exercise* is a self-report tool for exercise and will be completed weekly.
2) Please fill out each day of the week in terms of the exercise you perform to the best of your abilities as described below:
   - **Did you exercise?** At any point in the day, did you exercise? This is answered as "yes" or "no". This includes both planned exercise and any other physical activity that is completed that day.
   - **Type(s):** For each type of exercise bout you completed that day, record the type(s) of the exercise you performed (i.e., walking, swimming, weightlifting etc.)
   - **Time (minutes):** For each type of exercise bout you completed that day, record how long it took you to complete the bout in minutes. Time for each exercise should be listed in the same order that it was listed for type(s) of exercise.
   - **Borg Rating of Perceived Exertion (RPE) on a scale of 6 to 20:** Record the Borg RPE for each exercise bout you completed that day using the scale and instructions below. RPE for each exercise should be listed in the same order that it was listed for type(s) of exercise.

   Please refer to the *Timeline Followback for Exercise* for further instructions.
3) For each week of the exercise program, transfer your recordings to an electronic diary in REDCap by the first day of each week (Sundays by 11:59 PM).

Guidelines for Exercise Progression

Aerobic training:

"Start low and go slow"

1) Start at light-to-moderate intensity exercise
2) Increase exercise duration (time) per day by 5-10 min every 1-2 weeks.
3) Increase the number of days per week gradually over 12 weeks.
4) Increase exercise intensity when you perceive reductions in your exertion during exercise sessions and gradually transition to vigorous intensity exercise.

Resistance training:

1) When you can perform 2 more repetitions than what was prescribed during two consecutive sessions for a given exercise, increase the load by 2.5%-5%, all while maintaining proper form/technique.
2) Increase the number of days per week the muscle groups are trained over 12 weeks.
3) Increase the number of sets per muscle group per session gradually as tolerated.

Neuromotor training:

1) Increase exercise intensity by performing more challenging or advanced balances, postures, or movements over 12 weeks.

Flexibility training:

1) Increase the number of days per week of stretching over 12 weeks.

Contact Details for the UConn Graduate Research Assistant

Alexander Wright

Email: Alexander.Wright@hhchealth.org

Phone: (860) 486-6814

12 Week Exercise Program Progression Guidance

<table>
<tr><th></th><th>Aerobic</th><th>Resistance</th><th>Neuromotor</th><th>Flexibility</th></tr>
<tr><td>Week 1</td><td rowspan="3">2 days per week at light to moderate intensity for ~20-30 minutes</td><td rowspan="3">1 day per week at moderate intensity for ~30 minutes</td><td rowspan="3">1 day per week at light to moderate intensity for 20-30 minutes</td><td rowspan="6">2 days per week for ≥10 minutes</td></tr>
<tr><td>Week 2</td></tr>
<tr><td>Week 3</td></tr>
<tr><td>Week 4</td><td rowspan="3">2-3 days per week at moderate intensity for 20-30 minutes</td><td rowspan="3">2 days per week at moderate intensity for ~30 minutes</td><td rowspan="3">2 days per week at moderate intensity for 20-30 minutes</td></tr>
<tr><td>Week 5</td></tr>
<tr><td>Week 6</td></tr>
<tr><td>Week 7</td><td rowspan="3">≥2-3 days per week at moderate intensity for 20-30 minutes and/or vigorous intensity for 10-15 minutes</td><td rowspan="6">≥2-3 days per week at moderate to vigorous intensity for ~45 minutes</td><td rowspan="6">≥2-3 days per week at moderate intensity for ≥20-30 minutes</td><td rowspan="6">≥2-3 days per week for ≥10 minutes</td></tr>
<tr><td>Week 8</td></tr>
<tr><td>Week 9</td></tr>
<tr><td>Week 10</td><td rowspan="3">≥2-3 days per week at moderate intensity for ≥20-30 minutes and/or vigorous intensity for ≥10-15 minutes</td></tr>
<tr><td>Week 11</td></tr>
<tr><td>Week 12</td></tr>
</table>

Week 1

Aerobic exercise: 2 days per week for ~20-30 minutes per day at light to moderate intensity, to total 60 minutes per week.

Resistance exercise: 1 day per week for ~30 minutes per session at moderate intensity.

Neuromotor* exercise: 1 day per week for 20-30 minutes per session at light to moderate intensity. **Neuromotor functional body weight exercise can be substituted for resistance or flexibility exercise depending on personal preference.*

Flexibility exercise: 2 days per week for ≥10 minutes per session*, stretching to the point of tightness or slight discomfort. **Can be included in warm-ups or cool-downs.*

Aerobic Exercise Workout Examples at Light to Moderate Intensity Week 1.

Component	Time	Type	Examples
1) Warm-up	≥10 minutes	Aerobic activity or dynamic stretching	- Walking at a talking pace on a treadmill<br>- Walking to the gym<br>- Dynamic stretching (3-6 sets of 30-90 sec with 15 sec rest periods between sets):<br>• Marching in place<br>• Walking lunges<br>• Hip circles or openers<br>• Arm swings/arm circles<br>• Torso twists
2) Aerobic Exercise	~20-30 minutes	Steady state light to moderate intensity aerobic activity	- Walking at a pace that increases your breathing rate noticeably<br>- Peddling slowly on a stationary bicycle or leisurely cycling<br>- Peddling slowly on an elliptical machine<br>- Rowing slowly on a machine<br>- Slow dancing<br>- Aqua-aerobics
3) Cool-down	≥10 minutes	Static flexibility OR slowly reduce the pace of aerobic exercise	- Static stretching (10-30 seconds per stretch, 2-4 repetitions of each exercise):<br>• Standing calf stretch<br>• Quadricep stretch<br>• Kneeling hip flexor stretch (iliopsoas)<br>• Seated hamstring stretch<br>• Child’s pose stretch<br>• Chest stretch<br>• Cross-body shoulder stretch<br>• Neck lateral flexion stretch

Resistance Exercise Workout Examples at Moderate Intensity Week 1.

Component	Time	Type	Example exercises with machines, free weights, bodyweight, or resistance bands
Resistance Exercise (Day 1)	~30 minutes	Resistance training for all major muscle groups	2-3 sets of 8-12 repetitions (rest 1.5-2 minutes between sets)<br>RPE 12-13; 1-RM: 50-70%<br>• Chest press<br>• Seated row<br>• Leg Press<br>• Hip bridges<br>• Leg curls<br>• Arm curls<br>• Abdominal flexions<br>• Planks
Resistance Exercise (Day 2)	~30 minutes	Resistance training for all major muscle groups	2-3 sets of 8-12 repetitions (rest 1.5-2 minutes between sets):<br>RPE 12-13; 1-RM: 50-70%<br>• Shoulder press<br>• Lat pull-downs<br>• Hip kickbacks<br>• Hip abductions<br>• Leg extensions<br>• Triceps extensions<br>• Abdominal twists<br>• Side planks

Neuromotor Exercise Workout Examples at Light to Moderate Intensity Week 1.

Component	Time	Type	Examples
Neuromotor Exercise	20-30 minutes	Training that involves motor skills such as balance, coordination, agility	- Yoga (Hatha sitting/Vinyasa/ Nadisodhana)<br>- Tai chi (qi gong, sitting)<br>- Pilates (traditional)<br>- Balance exercise (15-30 seconds per hold, 5-6 cycles):<br>• two-legged stance<br>• semi-tandem stance<br>• reaching from a narrow stance<br>• tandem walking<br>• standing with eyes closed

Week 2

Aerobic exercise: 2 days per week for ~20-30 minutes per day at light to moderate intensity, to total 60 minutes per week.

Resistance exercise: 1 day per week for ~30 minutes per session at moderate intensity.

Neuromotor* exercise: 1 day per week for 20-30 minutes per session at light to moderate intensity. **Neuromotor functional body weight exercise can be substituted for resistance or flexibility exercise depending on personal preference.*

Flexibility exercise: 2 days per week for ≥10 minutes per session*, stretching to the point of tightness or slight discomfort. **Can be implemented into warm-ups or cool-downs.*

Aerobic Exercise Workout Examples at Light to Moderate Intensity Week 2.

Component	Time	Type	Examples
1) Warm-up	≥10 minutes	Aerobic activity or dynamic stretching	- Walking at a talking pace on a treadmill<br>- Walking to the gym<br>- Dynamic stretching (3-6 sets of 30-90 sec with 15 sec rest periods between sets):<br>• Marching in place<br>• Walking lunges<br>• Hip circles or openers<br>• Arm swings/arm circles<br>• Torso twists
2) Aerobic Exercise	~20-30 minutes	Steady state light to moderate intensity aerobic activity	- Walking at a pace that increases your breathing rate noticeably<br>- Peddling slowly on a stationary bicycle or leisurely cycling<br>- Peddling slowly on an elliptical machine<br>- Rowing slowly on a machine<br>- Slow dancing<br>- Aqua-aerobics
3) Cool-down	≥10 minutes	Static flexibility OR slowly reduce the pace of aerobic exercise	- Static stretching (10-30 seconds per stretch, 2-4 repetitions of each exercise):<br>• Standing calf stretch<br>• Quadricep stretch<br>• Kneeling hip flexor stretch (iliopsoas)<br>• Seated hamstring stretch<br>• Child's pose stretch<br>• Chest stretch<br>• Cross-body shoulder stretch<br>• Neck lateral flexion stretch

Resistance Exercise Workout Examples at Moderate Intensity Week 2.

Component	Time	Type	Example exercises with machines, free weights, bodyweight, or resistance bands
Resistance Exercise (Day 1)	~30 minutes	Resistance training for all major muscle groups	2-3 sets of 8-12 repetitions (rest 1.5-2 minutes between sets)<br>RPE 12-13; 1-RM: 50-70%<br>• Chest press<br>• Seated row<br>• Leg press<br>• Hip bridges<br>• Leg curls<br>• Arm curls<br>• Abdominal flexions<br>• Planks
Resistance Exercise (Day 2)	~30 minutes	Resistance training for all major muscle groups	2-3 sets of 8-12 repetitions (rest 1.5-2 minutes between sets)<br>RPE 12-13; 1-RM: 50-70%<br>• Shoulder press<br>• Lat pull-downs<br>• Hip kickbacks<br>• Hip abductions<br>• Leg extensions<br>• Triceps extensions<br>• Abdominal twists<br>• Side planks

Neuromotor Exercise Workout Examples at Light to Moderate Intensity Week 2.

Component	Time	Type	Examples
Neuromotor Exercise	20-30 minutes	Training that involves motor skills such as balance, coordination, agility	- Yoga (Hatha sitting/Vinyasa/ Nadisodhana)<br>- Tai chi (qi gong, sitting)<br>- Pilates (traditional)<br>- Balance exercise (15-30 seconds per hold, 5-6 cycles):<br>• two-legged stance<br>• semi-tandem stance<br>• reaching from a narrow stance<br>• tandem walking<br>• standing with eyes closed

Week 3

Aerobic exercise: 2 days per week for ~20-30 minutes per day at light to moderate intensity, to total 60 minutes per week.

Resistance exercise: 1 day per week for ~30 minutes per session at moderate intensity.

Neuromotor* exercise: 1 day per week for 20-30 minutes per session at light to moderate intensity. **Neuromotor functional body weight exercise can be substituted for resistance or flexibility exercise depending on personal preference.*

Flexibility exercise: 2 days per week for ≥10 minutes per session*, stretching to the point of tightness or slight discomfort. **Can be implemented into warm-ups or cool-downs.*

Aerobic Exercise Workout Examples at Light to Moderate Intensity Week 3.

Component	Time	Type	Examples
1) Warm-up	≥10 minutes	Aerobic activity or dynamic stretching	- Walking at a talking pace on a treadmill<br>- Walking to the gym<br>- Dynamic stretching (3-6 sets of 30-90 sec with 15 sec rest periods between sets):<br>• Marching in place<br>• Walking Lunges<br>• Hip circles or openers<br>• Arm swings/arm circles<br>• Torso twists
2) Aerobic Exercise	~20-30 minutes	Steady state light to moderate intensity aerobic activity	- Walking at a pace that increases your breathing rate noticeably<br>- Peddling slowly on a stationary bicycle or leisurely cycling<br>- Peddling slowly on an elliptical machine<br>- Rowing slowly on a machine<br>- Slow dancing<br>- Aqua-aerobics
3) Cool-down	≥10 minutes	Static flexibility OR slowly reduce the pace of aerobic exercise	- Static stretching (10-30 seconds per stretch, 2-4 repetitions of each exercise):<br>• Standing calf stretch<br>• Quadricep stretch<br>• Kneeling hip flexor stretch (iliopsoas)<br>• Seated hamstring stretch<br>• Child's pose stretch<br>• Chest stretch<br>• Cross-body shoulder stretch<br>• Neck lateral flexion stretch

Resistance Exercise Workout Examples at Moderate Intensity Week 3.

Component	Time	Type	Example exercises with machines, free weights, bodyweight, or resistance bands
Resistance Exercise (Day 1)	~30 minutes	Resistance training for all major muscle groups	2-3 sets of 8-12 repetitions (rest 1.5-2 minutes between sets)<br>RPE 12-13; 1-RM: 50-70%<br>• Chest press<br>• Seated row<br>• Leg press<br>• Hip bridges<br>• Leg curls<br>• Arm curls<br>• Abdominal flexions<br>• Planks
Resistance Exercise (Day 2)	~30 minutes	Resistance training for all major muscle groups	2-3 sets of 8-12 repetitions (rest 1.5-2 minutes between sets)<br>RPE 12-13; 1-RM: 50-70%<br>• Shoulder press<br>• Lat pull-downs<br>• Hip kickbacks<br>• Hip abductions<br>• Leg extensions<br>• Triceps extensions<br>• Abdominal twists<br>• Side planks

Neuromotor Exercise Workout Examples at Light to Moderate Intensity Week 3.

Component	Time	Type	Examples
Neuromotor Exercise	20-30 minutes	Training that involves motor skills such as balance, coordination, agility	- Yoga (Hatha sitting/Vinyasa/ Nadisodhana)<br>- Tai chi (qi gong, sitting)<br>- Pilates (traditional)<br>- Balance exercise (15-30 seconds per hold, 5-6 cycles):<br>• two-legged stance<br>• semi-tandem stance<br>• reaching from a narrow stance<br>• tandem walking<br>• standing with eyes closed

Week 4

Aerobic exercise: 2-3 days per week for 20-30 minutes per day at moderate intensity, to total 90 minutes per week.

Resistance exercise: 2 days per week for ~30 minutes per session at moderate intensity.

Neuromotor* exercise: 1 day per week for 20-30 minutes per session at light to moderate intensity. **Neuromotor functional body weight exercise can be substituted for resistance or flexibility exercise depending on personal preference.*

Flexibility exercise: 2 days per week for ≥10 minutes per session*, stretching to the point of tightness or slight discomfort. **Can be implemented into warm-ups or cool-downs.*

Aerobic Exercise Workout Examples at Moderate Intensity Week 4.

Component	Time	Type	Examples
1) Warm-up	≥10 minutes	Aerobic activity or dynamic stretching	- Walking at a talking pace on a treadmill<br>- Walking to the gym<br>- Dynamic stretching (3-6 sets of 30-90 sec with 15 sec rest periods between sets):<br>• Marching in place<br>• Walking lunges<br>• Hip circles or openers<br>• Arm swings/arm circles<br>• Torso twists
2) Aerobic Exercise	20-30 minutes	Steady state moderate intensity aerobic activity	- Walking at a pace that increases your breathing rate noticeably<br>- Peddling slowly on a stationary bicycle or leisurely cycling<br>- Peddling slowly on an elliptical machine<br>- Rowing slowly on a machine<br>- Slow dancing<br>- Aqua-aerobics
3) Cool-down	≥10 minutes	Static flexibility OR slowly reduce the pace of aerobic exercise	- Static stretching (10-30 seconds per stretch, 2-4 repetitions of each exercise):<br>• Standing calf stretch<br>• Quadricep stretch<br>• Kneeling hip flexor stretch (iliopsoas)<br>• Seated hamstring stretch<br>• Child's pose stretch<br>• Chest stretch<br>• Cross-body shoulder stretch<br>• Neck lateral flexion stretch

Resistance Exercise Workout Examples at Moderate Intensity Week 4.

Component	Time	Type	Example exercises with machines, free weights, bodyweight, or resistance bands
Resistance Exercise (Day 1)	~30 minutes	Resistance training for all major muscle groups	2-3 sets of 8-12 repetitions (rest 1.5-2 minutes between sets)<br>RPE 12-13; 1-RM: 50-70%<br>• Chest press<br>• Seated row<br>• Leg press<br>• Hip bridges<br>• Leg curls<br>• Arm curls<br>• Abdominal flexions<br>• Planks
Resistance Exercise (Day 2)	~30 minutes	Resistance training for all major muscle groups	2-3 sets of 8-12 repetitions (rest 1.5-2 minutes between sets)<br>RPE 12-13; 1-RM: 50-70%<br>• Shoulder press<br>• Lat pull-downs<br>• Hip kickbacks<br>• Hip abductions<br>• Leg extensions<br>• Triceps extensions<br>• Abdominal twists<br>• Side planks

Neuromotor Exercise Workout Examples at Light to Moderate Intensity Week 4.

Component	Time	Type	Examples
Neuromotor Exercise	20-30 minutes	Training that involves motor skills such as balance, coordination, agility	- Yoga (Hatha sitting/Vinyasa/ Nadisodhana)<br>- Tai chi (qi gong, sitting)<br>- Pilates (traditional)<br>- Balance exercise (15-30 seconds per hold, 5-6 cycles):<br>• two-legged stance<br>• semi-tandem stance<br>• reaching from a narrow stance<br>• tandem walking<br>• standing with eyes closed

Week 5

Aerobic exercise: 2-3 days per week for 20-30 minutes per day at moderate intensity, to total 90 minutes per week.

Resistance exercise: 2 days per week for ~30 minutes per session at moderate intensity.

Neuromotor* exercise: 2-3 days per week for 20-30 minutes per session at moderate intensity. **Neuromotor functional body weight exercise can be substituted for resistance or flexibility exercise depending on personal preference.*

Flexibility exercise: 2 days per week for ≥10 minutes per session*, stretching to the point of tightness or slight discomfort. **Can be implemented into warm-ups or cool-downs.*

Aerobic Exercise Workout Examples at Moderate Intensity Week 5.

Component	Time	Type	Examples
1) Warm-up	≥10 minutes	Aerobic activity or dynamic stretching	- Walking at a talking pace on a treadmill<br>- Walking to the gym<br>- Dynamic stretching (3-6 sets of 30-90 sec with 15 sec rest periods between sets):<br>• Marching in place<br>• Walking lunges<br>• Hip circles or openers<br>• Arm swings/arm circles<br>• Torso twists
2) Aerobic Exercise	20-30 minutes	Steady state moderate intensity aerobic activity	- Walking at a pace that increases your breathing rate noticeably<br>- Peddling slowly on a stationary bicycle or leisurely cycling<br>- Peddling slowly on an elliptical machine<br>- Rowing slowly on a machine<br>- Slow dancing<br>- Aqua-aerobics
3) Cool-down	≥10 minutes	Static flexibility OR slowly reduce the pace of aerobic exercise	- Static stretching (10-30 seconds per stretch, 2-4 repetitions of each exercise):<br>• Standing calf stretch<br>• Quadricep stretch<br>• Kneeling hip flexor stretch (iliopsoas)<br>• Seated hamstring stretch<br>• Child's pose stretch<br>• Chest stretch<br>• Cross-body shoulder stretch<br>• Neck lateral flexion stretch

Resistance Exercise Workout Examples at Moderate Intensity Week 5.

Component	Time	Type	Example exercises with machines, free weights, bodyweight, or resistance bands
Resistance Exercise (Day 1)	~30 minutes	Resistance training for all major muscle groups	2-3 sets of 8-12 repetitions (rest 1.5-2 minutes between sets)<br>RPE 12-13; 1-RM: 50-70%<br>• Chest press<br>• Seated row<br>• Leg press<br>• Hip bridges<br>• Leg curls<br>• Arm curls<br>• Abdominal flexions<br>• Planks
Resistance Exercise (Day 2)	~30 minutes	Resistance training for all major muscle groups	2-3 sets of 8-12 repetitions (rest 1.5-2 minutes between sets)<br>RPE 12-13; 1-RM: 50-70%<br>• Shoulder press<br>• Lat pull-downs<br>• Hip kickbacks<br>• Hip abductions<br>• Leg extensions<br>• Triceps extensions<br>• Abdominal twists<br>• Side planks

Neuromotor Exercise Workout Examples at Light to Moderate Intensity Week 5.

Component	Time	Type	Examples
Neuromotor Exercise	20-30 minutes	Training that involves motor skills such as balance, coordination, agility	- Yoga (Hatha sitting/Vinyasa/ Nadisodhana)<br>- Tai chi (qi gong, sitting)<br>- Pilates (traditional)<br>- Balance exercise (15-30 seconds per hold, 5-6 cycles):<br>• two-legged stance<br>• semi-tandem stance<br>• reaching from a narrow stance<br>• tandem walking<br>• standing with eyes closed

Week 6

Aerobic exercise: 2-3 days per week for 20-30 minutes per day at moderate intensity, to total 90 minutes per week.

Resistance exercise: 2 days per week for ~30 minutes per session at moderate intensity.

Neuromotor* exercise: 2-3 days per week for 20-30 minutes per session at moderate intensity. **Neuromotor functional body weight exercise can be substituted for resistance or flexibility exercise depending on personal preference.*

Flexibility exercise: 2 days per week for ≥10 minutes per session*, stretching to the point of tightness or slight discomfort. **Can be implemented into warm-ups or cool-downs.*

Aerobic Exercise Workout Examples at Moderate Intensity Week 6.

Component	Time	Type	Examples
1) Warm-up	≥10 minutes	Aerobic activity or dynamic stretching	- Walking at a talking pace on a treadmill<br>- Walking to the gym<br>- Dynamic stretching (3-6 sets of 30-90 sec with 15 sec rest periods between sets):<br>• Marching in place<br>• Walking lunges<br>• Hip circles or openers<br>• Arm swings/arm circles<br>• Torso twists
2) Aerobic Exercise	20-30 minutes	Steady state moderate intensity aerobic activity	- Walking at a pace that increases your breathing rate noticeably<br>- Peddling slowly on a stationary bicycle or leisurely cycling<br>- Peddling slowly on an elliptical machine<br>- Rowing slowly on a machine<br>- Slow dancing<br>- Aqua-aerobics
3) Cool-down	≥10 minutes	Static flexibility OR slowly reduce the pace of aerobic exercise	- Static stretching (10-30 seconds per stretch, 2-4 repetitions of each exercise):<br>• Standing calf stretch<br>• Quadricep stretch<br>• Kneeling hip flexor stretch (iliopsoas)<br>• Seated hamstring stretch<br>• Child’s pose stretch<br>• Chest stretch<br>• Cross-body shoulder stretch<br>• Neck lateral flexion stretch

Resistance Exercise Workout Examples at Moderate Intensity Week 6.

Component	Time	Type	Example exercises with machines, free weights, bodyweight, or resistance bands
Resistance Exercise (Day 1)	~30 minutes	Resistance training for all major muscle groups	2-3 sets of 8-12 repetitions (rest 1.5-2 minutes between sets)<br>RPE 12-13; 1-RM: 50-70%<br>• Chest press<br>• Seated row<br>• Leg press<br>• Hip bridges<br>• Leg curls<br>• Arm curls<br>• Abdominal flexions<br>• Planks
Resistance Exercise (Day 2)	~30 minutes	Resistance training for all major muscle groups	2-3 sets of 8-12 repetitions (rest 1.5-2 minutes between sets)<br>RPE 12-13; 1-RM: 50-70%<br>• Shoulder press<br>• Lat pull-downs<br>• Hip kickbacks<br>• Hip abductions<br>• Leg extensions<br>• Triceps extensions<br>• Abdominal twists<br>• Side planks

Neuromotor Exercise Workout Examples at Light to Moderate Intensity Week 6.

Component	Time	Type	Examples
Neuromotor Exercise	20-30 minutes	Training that involves motor skills such as balance, coordination, agility	- Yoga (Hatha sitting/Vinyasa/ Nadisodhana)<br>- Tai chi (qi gong, sitting)<br>- Pilates (traditional)<br>- Balance exercise (15-30 seconds per hold, 5-6 cycles):<br>• two-legged stance<br>• semi-tandem stance<br>• reaching from a narrow stance<br>• tandem walking<br>• standing with eyes closed

Week 7

Aerobic* exercise: ≥2-3 days per week for 20-30 minutes per day at moderate intensity or 10-15 minutes per day at vigorous** intensity (or a combination), to total 90 to 120 minutes per week. ***the greatest blood pressure reductions occur after vigorous intensity aerobic exercise.*

Resistance* exercise: 2-3 days per week for ~45 minutes per session at moderate to vigorous intensity.

**Engage in aerobic and/or resistance exercise, alone or combined, on most, preferably all days of the week.*

Neuromotor* exercise: 2-3 days per week for 20-30 minutes per session at moderate intensity. **Neuromotor functional body weight exercise can be substituted for resistance or flexibility exercise depending on personal preference.*

Flexibility exercise: ≥2-3 days per week for ≥10 minutes per session*, stretching to the point of tightness or slight discomfort. **Can be implemented into warm-ups or cool-downs.*

Aerobic Exercise Workout Examples at Moderate Intensity Week 7.

Component	Time	Type	Examples
1) Warm-up	≥10 minutes	Aerobic activity or dynamic stretching	- Walking at a talking pace on a treadmill<br>- Walking to the gym<br>- Dynamic stretching (3-6 sets of 30-90 sec with 15 sec rest periods between sets):<br>• Marching in place<br>• Walking lunges<br>• Hip circles or openers<br>• Arm swings/arm circles<br>• Torso twists
2) Aerobic Exercise	20-30 minutes<br><br>OR<br><br>10-15 minutes	Steady state moderate intensity aerobic activity<br><br>OR<br><br>Steady state vigorous intensity aerobic activity or high intensity interval training	- Walking at a pace that increases your breathing rate noticeably<br>- Peddling slowly on a stationary bicycle or leisurely cycling<br>- Peddling slowly on an elliptical machine<br>- Rowing slowly on a machine<br>- Slow dancing<br>- Aqua-aerobics<br><br>- Walking very briskly, jogging, or running<br>- Peddling fast on a stationary bicycle or cycling fast or uphill<br>- Peddling fast on an elliptical machine<br>- Rowing fast on a machine<br>- Stepping on the stair stepper machine<br>- Fast dancing<br>- Aerobics

3) Cool-down	≥10 minutes	Static flexibility OR slowly reduce the pace of aerobic exercise	- Static stretching (10-30 seconds per stretch, 2-4 repetitions of each exercise):<br>• Standing calf stretch<br>• Quadricep stretch<br>• Kneeling hip flexor stretch (iliopsoas)<br>• Seated hamstring stretch<br>• Child's pose stretch<br>• Chest stretch<br>• Cross-body shoulder stretch<br>• Neck lateral flexion stretch

Resistance Exercise Workout Examples at Moderate to Vigorous Intensity Week 7.

Component	Time	Type	Example exercises with machines, free weights, bodyweight, or resistance bands
Resistance Exercise (Day 1)	~45 minutes	Resistance training for all major muscle groups	3-4 sets of 8-12 repetitions (rest 1.5-2.5 minutes between sets)<br>RPE 12-13 to 14-17; 1-RM: 60-80%<br>• Chest press<br>• Seated row<br>• Leg press<br>• Hip bridges<br>• Leg curls<br>• Arm curls<br>• Abdominal flexions<br>• Planks
Resistance Exercise (Day 2)	~45 minutes	Resistance training for all major muscle groups	3-4 sets of 8-12 repetitions (rest 1.5-2.5 minutes between sets)<br>RPE 12-13 to 14-17; 1-RM: 60-80%<br>• Shoulder press<br>• Lat pull-downs<br>• Hip kickbacks<br>• Hip abductions<br>• Leg extensions<br>• Triceps extensions<br>• Abdominal twists<br>• Side planks
Resistance Exercise (Day 3)	~45 minutes	Resistance training for all major muscle groups	3-4 sets of 8-12 repetitions (rest 1.5-2.5 minutes between sets)<br>RPE 12-13 to 14-17; 1-RM: 60-80%)<br>• Pick 8-10 exercises from above to train major muscle groups

Neuromotor Exercise Workout Examples at Moderate Intensity Week 7.

Component	Time	Type	Examples
Neuromotor Exercise	20-30 minutes	Training that involves motor skills such as balance, coordination, agility	- Yoga (power/Surya Namaskar)<br>- Tai chi (qi gong standing/yang style)<br>- Pilates (POUND® with drumming)<br>- Single leg or Bosu ball exercises<br>- Balance exercises (15-30 seconds per hold, 5-6 cycles):<br>• tandem stance<br>• one-legged stance<br>• stepping over obstacles<br>• heel or toe walks<br>• walking while turning the head

Week 8

Aerobic* exercise: ≥2-3 days per week for 20-30 minutes per day at moderate intensity or 10-15 minutes per day at vigorous** intensity (or a combination), to total 90 to 120 minutes per week. ***the greatest blood pressure reductions occur after vigorous intensity aerobic exercise.*

Resistance* exercise: 2-3 days per week for ~45 minutes per session at moderate to vigorous intensity.

**Engage in aerobic and/or resistance exercise, alone or combined, on most, preferably all days of the week.*

Neuromotor* exercise: 2-3 days per week for 20-30 minutes per session at moderate intensity. **Neuromotor functional body weight exercise can be substituted for resistance or flexibility exercise depending on personal preference.*

Flexibility exercise: ≥2-3 days per week for ≥10 minutes per session*, stretching to the point of tightness or slight discomfort. **Can be implemented into warm-ups or cool-downs.*

Aerobic Exercise Workout Examples at Moderate Intensity Week 8.

Component	Time	Type	Examples
1) Warm-up	≥10 minutes	Aerobic activity or dynamic stretching	- Walking at a talking pace on a treadmill<br>- Walking to the gym<br>- Dynamic stretching (3-6 sets of 30-90 sec with 15 sec rest periods between sets):<br>• Marching in place<br>• Walking lunges<br>• Hip circles or openers<br>• Arm swings/arm circles<br>• Torso twists
2) Aerobic Exercise	20-30 minutes	Steady state moderate intensity aerobic activity	- Walking at a pace that increases your breathing rate noticeably<br>- Peddling slowly on a stationary bicycle or leisurely cycling<br>- Peddling slowly on an elliptical machine<br>- Rowing slowly on a machine<br>- Slow dancing
	OR	OR	- Aqua-aerobics
	10-15 minutes	Steady state vigorous intensity aerobic activity or high intensity interval training	- Walking very briskly, jogging, or running<br>- Peddling fast on a stationary bicycle or cycling fast or uphill<br>- Peddling fast on an elliptical machine<br>- Rowing fast on a machine<br>- Stepping on the stair stepper machine<br>- Fast dancing<br>- Aerobics

3) Cool-down	≥10 minutes	Static flexibility OR slowly reduce the pace of aerobic exercise	- Static stretching (10-30 seconds per stretch, 2-4 repetitions of each exercise):<br>• Standing calf stretch<br>• Quadricep stretch<br>• Kneeling hip flexor stretch (iliopsoas)<br>• Seated hamstring stretch<br>• Child's pose stretch<br>• Chest stretch<br>• Cross-body shoulder stretch<br>• Neck lateral flexion stretch

Resistance Exercise Workout Examples at Moderate to Vigorous Intensity Week 8.

Component	Time	Type	Example exercises with machines, free weights, bodyweight, or resistance bands
Resistance Exercise (Day 1)	~45 minutes	Resistance training for all major muscle groups	3-4 sets of 8-12 repetitions (rest 1.5-2.5 minutes between sets)<br>RPE 12-13 to 14-17; 1-RM: 60-80%<br>• Chest press<br>• Seated row<br>• Leg press<br>• Hip bridges<br>• Leg curls<br>• Arm curls<br>• Abdominal flexions<br>• Planks
Resistance Exercise (Day 2)	~45 minutes	Resistance training for all major muscle groups	3-4 sets of 8-12 repetitions (rest 1.5-2.5 minutes between sets)<br>RPE 12-13 to 14-17; 1-RM: 60-80%<br>• Shoulder press<br>• Lat pull-downs<br>• Hip kickbacks<br>• Hip abductions<br>• Leg extensions<br>• Triceps extensions<br>• Abdominal twists<br>• Side planks
Resistance Exercise (Day 3)	~45 minutes	Resistance training for all major muscle groups	3-4 sets of 8-12 repetitions (rest 1.5-2.5 minutes between sets)<br>RPE 12-13 to 14-17; 1-RM: 60-80%)<br>• Pick 8-10 exercises from above to train major muscle groups

Neuromotor Exercise Workout Examples at Moderate Intensity Week 8.

Component	Time	Type	Examples
Neuromotor Exercise	20-30 minutes	Training that involves motor skills such as balance, coordination, agility	- Yoga (power/Surya Namaskar)<br>- Tai chi (qi gong standing/yang style)<br>- Pilates (POUND® with drumming)<br>- Single leg or Bosu ball exercises<br>- Balance exercises (15-30 seconds per hold, 5-6 cycles):<br>• tandem stance<br>• one-legged stance<br>• stepping over obstacles<br>• heel or toe walks<br>• walking while turning the head

Week 9

Aerobic* exercise: ≥2-3 days per week for 20-30 minutes per day at moderate intensity or 10-15 minutes per day at vigorous** intensity (or a combination), to total 90 to 120 minutes per week. ***the greatest blood pressure reductions occur after vigorous intensity aerobic exercise.*

Resistance* exercise: ≥2-3 days per week for ~45 minutes per session at moderate to vigorous intensity.

**Engage in aerobic and/or resistance exercise, alone or combined, on most, preferably all days of the week.*

Neuromotor* exercise: ≥2-3 days per week for ≥20-30 minutes per session at moderate intensity. **Neuromotor functional body weight exercise can be substituted for resistance or flexibility exercise depending on personal preference.*

Flexibility exercise: ≥2-3 days per week for ≥10 minutes per session*, stretching to the point of tightness or slight discomfort. **Can be implemented into warm-ups or cool-downs.*

Aerobic Exercise Workout Examples at Moderate Intensity Week 9.

Component	Time	Type	Examples
1) Warm-up	≥10 minutes	Aerobic activity or dynamic stretching	- Walking at a talking pace on a treadmill<br>- Walking to the gym<br>- Dynamic stretching (3-6 sets of 30-90 sec with 15 sec rest periods between sets):<br>• Marching in place<br>• Walking lunges<br>• Hip circles or openers<br>• Arm swings/arm circles<br>• Torso twists
2) Aerobic Exercise	20-30 minutes<br><br>OR<br><br>10-15 minutes	Steady state moderate intensity aerobic activity<br><br>OR<br><br>Steady state vigorous intensity aerobic activity or high intensity interval training	- Walking at a pace that increases your breathing rate noticeably<br>- Peddling slowly on a stationary bicycle or leisurely cycling<br>- Peddling slowly on an elliptical machine<br>- Rowing slowly on a machine<br>- Slow dancing<br>- Aqua-aerobics<br><br>- Walking very briskly, jogging, or running<br>- Peddling fast on a stationary bicycle or cycling fast or uphill<br>- Peddling fast on an elliptical machine<br>- Rowing fast on a machine<br>- Stepping on the stair stepper machine<br>- Fast dancing<br>- Aerobics

3) Cool-down	≥10 minutes	Static flexibility OR slowly reduce the pace of aerobic exercise	- Static stretching (10-30 seconds per stretch, 2-4 repetitions of each exercise):<br>• Standing calf stretch<br>• Quadricep stretch<br>• Kneeling hip flexor stretch (iliopsoas)<br>• Seated hamstring stretch<br>• Child's pose stretch<br>• Chest stretch<br>• Cross-body shoulder stretch<br>• Neck lateral flexion stretch

Resistance Exercise Workout Examples at Moderate to Vigorous Intensity Week 9.

Component	Time	Type	Example exercises with machines, free weights, bodyweight, or resistance bands
Resistance Exercise (Day 1)	~45 minutes	Resistance training for all major muscle groups	3-4 sets of 8-12 repetitions (rest 1.5-2.5 minutes between sets)<br>RPE 12-13 to 14-17; 1-RM: 60-80%<br>• Chest press<br>• Seated row<br>• Leg press<br>• Hip bridges<br>• Leg curls<br>• Arm curls<br>• Abdominal flexions<br>• Planks
Resistance Exercise (Day 2)	~45 minutes	Resistance training for all major muscle groups	3-4 sets of 8-12 repetitions (rest 1.5-2.5 minutes between sets)<br>RPE 12-13 to 14-17; 1-RM: 60-80%<br>• Shoulder press<br>• Lat pull-downs<br>• Hip kickbacks<br>• Hip abductions<br>• Leg extensions<br>• Triceps extensions<br>• Abdominal twists<br>• Side planks
Resistance Exercise (Day 3)	~45 minutes	Resistance training for all major muscle groups	3-4 sets of 8-12 repetitions (rest 1.5-2.5 minutes between sets)<br>RPE 12-13 to 14-17; 1-RM: 60-80%)<br>• Pick 8-10 exercises from above to train major muscle groups

Neuromotor Exercise Workout Examples at Moderate Intensity Week 9.

Component	Time	Type	Examples
Neuromotor Exercise	≥20-30 minutes	Training that involves motor skills such as balance, coordination, agility	- Yoga (power/Surya Namaskar)<br>- Tai chi (qi gong standing/yang style)<br>- Pilates (POUND® with drumming)<br>- Single leg or Bosu ball exercises<br>- Balance exercises (15-30 seconds per hold, 5-6 cycles):<br>• tandem stance<br>• one-legged stance<br>• stepping over obstacles<br>• heel or toe walks<br>• walking while turning the head

Week 10

Aerobic* exercise: ≥2-3 days per week for ≥20-30 minutes per day at moderate intensity or ≥10-15 minutes per day at vigorous** intensity (or a combination), to total 90 to 150 minutes per week. ***the greatest blood pressure reductions occur after vigorous intensity aerobic exercise.*

Resistance* exercise: ≥2-3 days per week for ~45 minutes per session at moderate to vigorous intensity.

**Engage in aerobic and/or resistance exercise, alone or combined, on most, preferably all days of the week.*

Neuromotor* exercise: ≥2-3 days per week for ≥20-30 minutes per session at moderate intensity. **Neuromotor functional body weight exercise can be substituted for resistance or flexibility exercise depending on personal preference.*

Flexibility exercise: ≥2-3 days per week for ≥10 minutes per session*, stretching to the point of tightness or slight discomfort. **Can be implemented into warm-ups or cool-downs.*

Aerobic Exercise Workout Examples at Moderate Intensity Week 10.

Component	Time	Type	Examples
1) Warm-up	≥10 minutes	Aerobic activity or dynamic stretching	- Walking at a talking pace on a treadmill<br>- Walking to the gym<br>- Dynamic stretching (3-6 sets of 30-90 sec with 15 sec rest periods between sets):<br>• Marching in place<br>• Walking lunges<br>• Hip circles or openers<br>• Arm swings/arm circles<br>• Torso twists
2) Aerobic Exercise	≥20-30 minutes<br><br>OR<br><br>≥10-15 minutes	Steady state moderate intensity aerobic activity<br><br>OR<br><br>Steady state vigorous intensity aerobic activity or high intensity interval training	- Walking at a pace that increases your breathing rate noticeably<br>- Peddling slowly on a stationary bicycle or leisurely cycling<br>- Peddling slowly on an elliptical machine<br>- Rowing slowly on a machine<br>- Slow dancing<br>- Aqua-aerobics<br><br>- Walking very briskly, jogging, or running<br>- Peddling fast on a stationary bicycle or cycling fast or uphill<br>- Peddling fast on an elliptical machine<br>- Rowing fast on a machine<br>- Stepping on the stair stepper machine<br>- Fast dancing<br>- Aerobics

3) Cool-down	≥10 minutes	Static flexibility OR slowly reduce the pace of aerobic exercise	- Static stretching (10-30 seconds per stretch, 2-4 repetitions of each exercise):<br>• Standing calf stretch<br>• Quadricep stretch<br>• Kneeling hip flexor stretch (iliopsoas)<br>• Seated hamstring stretch<br>• Child's pose stretch<br>• Chest stretch<br>• Cross-body shoulder stretch<br>• Neck lateral flexion stretch

Resistance Exercise Workout Examples at Moderate to Vigorous Intensity Week 10.

Component	Time	Type	Example exercises with machines, free weights, bodyweight, or resistance bands
Resistance Exercise (Day 1)	~45 minutes	Resistance training for all major muscle groups	3-4 sets of 8-12 repetitions (rest 1.5-2.5 minutes between sets)<br>RPE 12-13 to 14-17; 1-RM: 60-80%<br>• Chest press<br>• Seated row<br>• Leg press<br>• Hip bridges<br>• Leg curls<br>• Arm curls<br>• Abdominal flexions<br>• Planks
Resistance Exercise (Day 2)	~45 minutes	Resistance training for all major muscle groups	3-4 sets of 8-12 repetitions (rest 1.5-2.5 minutes between sets)<br>RPE 12-13 to 14-17; 1-RM: 60-80%<br>• Shoulder press<br>• Lat pull-downs<br>• Hip kickbacks<br>• Hip abductions<br>• Leg extensions<br>• Triceps extensions<br>• Abdominal twists<br>• Side planks
Resistance Exercise (Day 3)	~45 minutes	Resistance training for all major muscle groups	3-4 sets of 8-12 repetitions (rest 1.5-2.5 minutes between sets)<br>RPE 12-13 to 14-17; 1-RM: 60-80%)<br>• Pick 8-10 exercises from above to train major muscle groups

Neuromotor Exercise Workout Examples at Moderate Intensity Week 10.

Component	Time	Type	Examples
Neuromotor Exercise	≥20-30 minutes	Training that involves motor skills such as balance, coordination, agility	- Yoga (power/Surya Namaskar)<br>- Tai chi (qi gong standing/yang style)<br>- Pilates (POUND® with drumming)<br>- Single leg or Bosu ball exercises<br>- Balance exercises (15-30 seconds per hold, 5-6 cycles):<br>• tandem stance<br>• one-legged stance<br>• stepping over obstacles<br>• heel or toe walks<br>• walking while turning the head

Week 11

Aerobic* exercise: ≥2-3 days per week for ≥20-30 minutes per day at moderate intensity or ≥10-15 minutes per day at vigorous** intensity (or a combination), to total 90 to 150 minutes per week. ***the greatest blood pressure reductions occur after vigorous intensity aerobic exercise.*

Resistance* exercise: ≥2-3 days per week for ~45 minutes per session at moderate to vigorous intensity.

**Engage in aerobic and/or resistance exercise, alone or combined, on most, preferably all days of the week.*

Neuromotor* exercise: ≥2-3 days per week for ≥20-30 minutes per session at moderate intensity. **Neuromotor functional body weight exercise can be substituted for resistance or flexibility exercise depending on personal preference.*

Flexibility exercise: ≥2-3 days per week for ≥10 minutes per session*, stretching to the point of tightness or slight discomfort. **Can be implemented into warm-ups or cool-downs.*

Aerobic Exercise Workout Examples at Moderate Intensity Week 11.

Component	Time	Type	Examples
1) Warm-up	≥10 minutes	Aerobic activity or dynamic stretching	- Walking at a talking pace on a treadmill<br>- Walking to the gym<br>- Dynamic stretching (3-6 sets of 30-90 sec with 15 sec rest periods between sets):<br>• Marching in place<br>• Walking lunges<br>• Hip circles or openers<br>• Arm swings/arm circles<br>• Torso twists
2) Aerobic Exercise	≥20-30 minutes<br><br>OR<br><br>≥10-15 minutes	Steady state moderate intensity aerobic activity<br><br>OR<br><br>Steady state vigorous intensity aerobic activity or high intensity interval training	- Walking at a pace that increases your breathing rate noticeably<br>- Peddling slowly on a stationary bicycle or leisurely cycling<br>- Peddling slowly on an elliptical machine<br>- Rowing slowly on a machine<br>- Slow dancing<br>- Aqua-aerobics<br><br>- Walking very briskly, jogging, or running<br>- Peddling fast on a stationary bicycle or cycling fast or uphill<br>- Peddling fast on an elliptical machine<br>- Rowing fast on a machine<br>- Stepping on the stair stepper machine<br>- Fast dancing<br>- Aerobics

3) Cool-down	≥10 minutes	Static flexibility OR slowly reduce the pace of aerobic exercise	- Static stretching (10-30 seconds per stretch, 2-4 repetitions of each exercise):<br>• Standing calf stretch<br>• Quadricep stretch<br>• Kneeling hip flexor stretch (iliopsoas)<br>• Seated hamstring stretch<br>• Child's pose stretch<br>• Chest stretch<br>• Cross-body shoulder stretch<br>• Neck lateral flexion stretch

Resistance Exercise Workout Examples at Moderate to Vigorous Intensity Week 11.

Component	Time	Type	Example exercises with machines, free weights, bodyweight, or resistance bands
Resistance Exercise (Day 1)	~45 minutes	Resistance training for all major muscle groups	3-4 sets of 8-12 repetitions (rest 1.5-2.5 minutes between sets)<br>RPE 12-13 to 14-17; 1-RM: 60-80%<br>• Chest press<br>• Seated row<br>• Leg press<br>• Hip bridges<br>• Leg curls<br>• Arm curls<br>• Abdominal flexions<br>• Planks
Resistance Exercise (Day 2)	~45 minutes	Resistance training for all major muscle groups	3-4 sets of 8-12 repetitions (rest 1.5-2.5 minutes between sets)<br>RPE 12-13 to 14-17; 1-RM: 60-80%<br>• Shoulder press<br>• Lat pull-downs<br>• Hip kickbacks<br>• Hip abductions<br>• Leg extensions<br>• Triceps extensions<br>• Abdominal twists<br>• Side planks
Resistance Exercise (Day 3)	~45 minutes	Resistance training for all major muscle groups	3-4 sets of 8-12 repetitions (rest 1.5-2.5 minutes between sets)<br>RPE 12-13 to 14-17; 1-RM: 60-80%)<br>• Pick 8-10 exercises from above to train major muscle groups

Neuromotor Exercise Workout Examples at Moderate Intensity Week 11.

Component	Time	Type	Examples
Neuromotor Exercise	≥20-30 minutes	Training that involves motor skills such as balance, coordination, agility	- Yoga (power/Surya Namaskar)<br>- Tai chi (qi gong standing/yang style)<br>- Pilates (POUND® with drumming)<br>- Single leg or Bosu ball exercises<br>- Balance exercises (15-30 seconds per hold, 5-6 cycles):<br>• tandem stance<br>• one-legged stance<br>• stepping over obstacles<br>• heel or toe walks<br>• walking while turning the head

Week 12

Aerobic* exercise: ≥2-3 days per week for ≥20-30 minutes per day at moderate intensity or ≥10-15 minutes per day at vigorous** intensity (or a combination), to total 90 to 150 minutes per week. ***the greatest blood pressure reductions occur after vigorous intensity aerobic exercise.*

Resistance* exercise: ≥2-3 days per week for ~45 minutes per session at moderate to vigorous intensity.

**Engage in aerobic and/or resistance exercise, alone or combined, on most, preferably all days of the week.*

Neuromotor* exercise: ≥2-3 days per week for ≥20-30 minutes per session at moderate intensity. **Neuromotor functional body weight exercise can be substituted for resistance or flexibility exercise depending on personal preference.*

Flexibility exercise: ≥2-3 days per week for ≥10 minutes per session*, stretching to the point of tightness or slight discomfort. **Can be implemented into warm-ups or cool-downs.*

Aerobic Exercise Workout Examples at Moderate Intensity Week 12.

Component	Time	Type	Examples
1) Warm-up	≥10 minutes	Aerobic activity or dynamic stretching	- Walking at a talking pace on a treadmill<br>- Walking to the gym<br>- Dynamic stretching (3-6 sets of 30-90 sec with 15 sec rest periods between sets):<br>• Marching in place<br>• Walking lunges<br>• Hip circles or openers<br>• Arm swings/arm circles<br>• Torso twists
2) Aerobic Exercise	≥20-30 minutes<br><br>OR<br><br>≥10-15 minutes	Steady state moderate intensity aerobic activity<br><br>OR<br><br>Steady state vigorous intensity aerobic activity or high intensity interval training	- Walking at a pace that increases your breathing rate noticeably<br>- Peddling slowly on a stationary bicycle or leisurely cycling<br>- Peddling slowly on an elliptical machine<br>- Rowing slowly on a machine<br>- Slow dancing<br>- Aqua-aerobics<br><br>- Walking very briskly, jogging, or running<br>- Peddling fast on a stationary bicycle or cycling fast or uphill<br>- Peddling fast on an elliptical machine<br>- Rowing fast on a machine<br>- Stepping on the stair stepper machine<br>- Fast dancing<br>- Aerobics

3) Cool-down	≥10 minutes	Static flexibility OR slowly reduce the pace of aerobic exercise	- Static stretching (10-30 seconds per stretch, 2-4 repetitions of each exercise):<br>• Standing calf stretch<br>• Quadricep stretch<br>• Kneeling hip flexor stretch (iliopsoas)<br>• Seated hamstring stretch<br>• Child's pose stretch<br>• Chest stretch<br>• Cross-body shoulder stretch<br>• Neck lateral flexion stretch

Resistance Exercise Workout Examples at Moderate to Vigorous Intensity Week 12.

Component	Time	Type	Example exercises with machines, free weights, bodyweight, or resistance bands
Resistance Exercise (Day 1)	~45 minutes	Resistance training for all major muscle groups	3-4 sets of 8-12 repetitions (rest 1.5-2.5 minutes between sets)<br>RPE 12-13 to 14-17; 1-RM: 60-80%<br>• Chest press<br>• Seated row<br>• Leg press<br>• Hip bridges<br>• Leg curls<br>• Arm curls<br>• Abdominal flexions<br>• Planks
Resistance Exercise (Day 2)	~45 minutes	Resistance training for all major muscle groups	3-4 sets of 8-12 repetitions (rest 1.5-2.5 minutes between sets)<br>RPE 12-13 to 14-17; 1-RM: 60-80%<br>• Shoulder press<br>• Lat pull-downs<br>• Hip kickbacks<br>• Hip abductions<br>• Leg extensions<br>• Triceps extensions<br>• Abdominal twists<br>• Side planks
Resistance Exercise (Day 3)	~45 minutes	Resistance training for all major muscle groups	3-4 sets of 8-12 repetitions (rest 1.5-2.5 minutes between sets)<br>RPE 12-13 to 14-17; 1-RM: 60-80%)<br>• Pick 8-10 exercises from above to train major muscle groups

Neuromotor Exercise Workout Examples at Moderate Intensity Week 12.

Component	Time	Type	Examples
Neuromotor Exercise	≥20-30 minutes	Training that involves motor skills such as balance, coordination, agility	- Yoga (power/Surya Namaskar)<br>- Tai chi (qi gong standing/yang style)<br>- Pilates (POUND® with drumming)<br>- Single leg or Bosu ball exercises<br>- Balance exercises (15-30 seconds per hold, 5-6 cycles):<br>• tandem stance<br>• one-legged stance<br>• stepping over obstacles<br>• heel or toe walks<br>• walking while turning the head

Participant 12-Week Exercise Program Information Packet

Participant ID:

Date Provided:

12-Week Start Date:

12-Week End Date:

Principle Investigators:

Dr. Antonio Fernandez, MD, FACC, FAHA
Medical Director of Preventive Cardiology, Hartford Hospital

Dr. Linda Pescatello, PhD, FACSM
Board of Trustees Distinguished Professor of Kinesiology, University of Connecticut

Dr. Peter Robinson, MD
Assistant Professor of Cardiology, UConn Health

UConn Graduate Research Assistant:

Alexander Wright, MS
Email: Alexander.Wright@hhchealth.org
Phone: (860) 486-6814

This research is approved by the Hartford HealthCare Institutional Review Board, with reliance agreements at UConn Storrs and UConn Health.

Contents Page

Exercise Program for Dyslipidemia 3
Special Considerations 4
Exercise Type Definitions 5
Exercise Type Examples 6
Exercise Intensity Definitions 8
How to Take Your Pulse 9
Self-Monitor Your Physical Activity 9
Guidelines for Exercise Progression 10
Contact Details for the UConn Graduate Research Assistant 10
12 Week Exercise Program Progression Guidance 11
Week 1 12
Week 2 14
Week 3 16
Week 4 18
Week 5 20
Week 6 22
Week 7 24
Week 8 26
Week 9 28
Week 10 30
Week 11 32
Week 12 34

Exercise Program for Dyslipidemia

FITT	Aerobic	Resistance	Neuromotor*	Flexibility
Frequency	≥5 days per week to maximize caloric expenditure	2-3 days per week	≥2-3 days per week	≥2-3 days per week
Intensity	40%-75% VO_2R or HRR<br>**Moderate intensity:** you can talk comfortably but not sing<br>64-76% HRmax; 40-59% VO_2R or HRR; RPE 12-13<br>to **vigorous intensity:** you cannot say more than 5 words without grasping for breath<br>77-95% HRmax; ≥60% VO_2R or HRR; RPE 14-17	**Moderate intensity:** 50-69% of 1-RM for 12-18 reps<br>to **vigorous intensity:** 70-85% of 1-RM for 8-12 repetitions to improve strength<br><50% 1-RM to improve muscle endurance.	Not determined.	Stretch to the point of tightness or slight discomfort.
Time	30-60 minutes per day to promote or maintain weight loss, 50-60 minutes per day or more of daily exercise is recommended.	2-4 sets, 8-12 repetitions for strength; ≤2 sets, 12-20 repetitions for muscular endurance.	≥20-30 minutes per day	Hold static stretch for 10-30 seconds; 2-4 repetitions of each exercise.
Type	Prolonged, rhythmic activities using large muscle groups (e.g. walking, cycling, swimming)	Resistance machines, free weights, and/or body weight	Exercise involving motor skills and/or functional body weight and flexibility exercise such as yoga, pilates, and tai chi.	Static, dynamic, and/or PNF stretching

FITT = Frequency, Intensity, Time and Type; 1-RM = 1 Repetition Maximum; RPE = Rating of Perceived Exertion (Borg 6-20 scale); HRmax = Heart Rate Maximum; HRR = Heart Rate Reserve; VO_2R = Oxygen Uptake Reserve; PNF = Proprioceptive Neuromuscular Facilitation.

*Neuromotor functional body weight exercise can be substituted for resistance exercise, and depending on the amount of flexibility exercise integrated into a session, neuromotor flexibility exercise can be substituted for flexibility exercise depending on patient/client preference.

Reference: American College of Sports Medicine, Ozemek C, Bonikowske AR, Christle JW, Gallo PM, eds. ACSM's Guidelines for Exercise Testing and Prescription. 12th Ed, p.360. Wolters Kluwer; 2026.

Special Considerations

Medication Considerations: Yes/No

If YES, list medication(s) taken and dose:

If YES, list potential side effects of medication as related to exercise.

If YES, list special considerations for the medication as related to exercise.

Exercise Type Definitions

Aerobic Exercise: Continuous exercise involving large muscle groups such as walking, running, riding a bicycle, cardio machines and rowing.

> **Steady State Aerobic Exercise:** Maintaining exercise at light to moderate intensity.
>
> **High Intensity Interval Training:** Performing bouts of higher intensity exercise separated by rest.

Resistance Exercise: Exercises that use opposing forces to strengthen or develop muscles such as weight training with free weights, resistance machines, or using resistance bands or body weight.

Neuromotor Exercise: Exercises that combine balance, coordination, and agility such as yoga, tai chi, and Pilates.

Flexibility: Movements that improve the range of motion of a joint.

> **Dynamic Flexibility:** Slow moving stretches that increase reach and range of motion as the movement is repeated. Examples: Leg swings, arm swings, torso twists.
>
> **Static Flexibility:** Slowly stretching a muscle/tendon group and holding the position for 10-30 sec. Examples: Pike stretch, glute stretch, quad stretch
>
> **Proprioceptive Neuromuscular Facilitation (PNF):** Applying force with a muscle against an opposing force followed by performing a static stretch of the muscle.

Concurrent Exercise: Performing aerobic and resistance exercise in the same session or near one another.

Major Muscle Groups: Shoulders, chest, back, hamstrings, quadriceps, calves, biceps, triceps, core.

Exercise Type Examples

Aerobic Exercise

Light Intensity (RPE 8-11)	Moderate Intensity (RPE 12-13)	Vigorous Intensity (RPE 14-20)
Walking slowly, leisurely	Walking the dog or walking outside	Brisk walking or jogging
Cycling slowly with a pedal desk	Cycling at a self-selected comfortable pace	Cycling at a moderate pace
Group class - Vinyasa yoga, tai chi (qi gong), or stretching	Group class – Power yoga or tai chi (yang style)	Group class - Zumba or fast ballroom dancing
Cleaning, sweeping, or washing dishes slowly	Water aerobics	Swimming laps, freestyle
Pickleball and Tennis, serving practice	Pickleball and Tennis, doubles	Pickleball and Tennis, singles

Resistance Exercise

Light Intensity (RPE 8-11)	Moderate Intensity (RPE 12-13)	Vigorous Intensity (RPE 14-20)
Yard work at a slow pace	Gardening - watering, weeding, planting	Gardening - using heavy tools, digging or filling garden
Canoeing at a slow pace	Kayaking at a self-selected comfortable pace	Rowing on a stationary ergometer
Group class - Vinyasa yoga, tai chi (qi gong)	Group class – Power yoga or tai chi (yang style)	Group class - circuit training with minimal rest
Bodyweight exercises (curl-ups, planks)	Bodyweight exercises (push-ups, lunges)	Bodyweight exercises (jumping jacks, burpees)
Lifting weights <50% of one repetition maximum (being able to perform 15-20 reps at this weight)	Lifting weights 50-69% of one repetition maximum (being able to perform 12-18 reps at this weight)	Lifting weights 70 to 85% of one repetition maximum (being able to perform 8-12 reps at this weight)

Neuromotor Exercise

Light to Moderate Intensity (RPE 8-13)	Moderate Intensity (RPE 12-13)
Yoga (Hatha sitting/Vinyasa/ Nadisodhana)	Yoga (power/Surya Namaskar)
Tai chi (qi gong, sitting)	Tai chi (qi gong standing/yang style)
Pilates (traditional)	Pilates (POUND® with drumming)
Balance exercises (beginner)	Balance exercises (general)
Slow dancing	Functional bodyweight exercises

Exercise Intensity Definitions

Adapted Version of Borg 6-20 Rating of Perceived Exertion (RPE) Scale with Exercise Intensity Differentiation:

Borg 6-20 RPE	Intensity	Intensity Definition
6	No exertion	
7	Very Light	
8		
9	Light	A level of physical exertion that causes slight increases in heart rate and breathing (i.e., warm up with dynamic flexibility, cool down with slow aerobic movements and static stretching). ***Use the talk test:*** *Light intensity should make your breathing slightly increase, but you can still talk and sing easily.*<br>Aerobic exercise: <64% HRmax, <40% VO_2R or HRR
10		
11		
12	Moderate	A level of physical exertion that causes increases in heart rate and breathing (i.e., brisk walking, weight training at an intensity that is not hard, but takes effort). ***Use the talk test:*** *Moderate intensity should make your breathing rate increase noticeably. You can still talk but not sing.*<br>Aerobic exercise: 64-76% HRmax, 40-59% VO_2R or HRR.<br>Resistance exercise: 50-69% of 1-RM intensity of 12-18 reps.
13		
14	Vigorous	A level of physical exertion that causes substantial increases in heart rate and breathing (i.e., running, weight training until fatigue). ***Use the talk test:*** *Vigorous intensity should make you breathe hard enough that you can only say a few words before you have to take a breath and you can't sing.*<br>Aerobic exercise: 77-95% HRmax, ≥ 60% VO_2R or HRR<br>Resistance exercise: 70-85% of 1-RM intensity of 8-12 reps.
15		
16		
17		
18	Near Maximal to Maximal	
19		
20		

HRmax = Heart Rate Maximum. The HRmax is the maximum heart rate you can achieve during exercise. HRmax declines with age.

HRR = Heart Rate Reserve. The percentage of HRR is calculated as: (the heart rate during exercise – the heart rate at rest) ÷ (the maximum heart rate during exercise – the heart rate at rest) × 100%.

VO_2R = Oxygen Uptake Reserve. The percentage of VO_2R is calculated as: (the rate of oxygen consumption during exercise – the rate of oxygen consumption at rest) ÷ (the maximum rate of oxygen consumption during exercise – the rate of oxygen consumption at rest) × 100%.

1-RM = 1 Repetition Maximum. The 1-RM is the maximum weight lifted for a single repetition for a given exercise.

How to Take Your Pulse

1) You can use a technique called pulse palpitation, which involves “feeling” the pulse.
2) Place your index finger and middle fingers over the radial artery, located near the thump side of the wrist.
3) Count the pulse for 30-60 seconds. The 30-second count is multiplied by 2 to determine the 1-minute resting heart rate in beats per minute (bpm).

Self-Monitor Your Physical Activity

1) The *Timeline Followback for Exercise* is a self-report tool for exercise and will be completed daily.

2) Please fill out each day of the week in terms of the exercise you perform to the best of your abilities as described below:

   - **Did you exercise?** At any point in the day, did you exercise? This is answered as “yes” or “no”. This includes both planned exercise and any other physical activity that is completed that day.
   - **Type(s):** For each type of exercise bout you completed that day, record the type(s) of the exercise you performed (i.e., walking, swimming, weightlifting etc.)
   - **Time (minutes):** For each type of exercise bout you completed that day, record how long it took you to complete the bout in minutes. Time for each exercise should be listed in the same order that it was listed for type(s) of exercise.
   - **Borg Rating of Perceived Exertion (RPE) on a scale of 6 to 20:** Record the Borg RPE for each exercise bout you completed that day using the scale and instructions below. RPE for each exercise should be listed in the same order that it was listed for type(s) of exercise.

   Please refer to the *Timeline Followback for Exercise* for further instructions.

3) For each week of the exercise program, transfer your recordings to an electronic diary in REDCap by the first day of each week (Sundays by 11:59 PM).

Guidelines for Exercise Progression

Aerobic training:

"Start low and go slow"

1) Start at light-to-moderate intensity exercise
2) Increase exercise duration (time) per day by 5-10 min every 1-2 weeks.
3) Increase the number of days per week gradually over 12 weeks.
4) Increase exercise intensity when you perceive reductions in your exertion during exercise sessions and gradually transition to vigorous intensity exercise.

Resistance training:

1) When you can perform 2 more repetitions than what was prescribed during two consecutive sessions for a given exercise, increase the load by 2.5%-5%, all while maintaining proper form/technique.
2) Increase the number of days per week the muscle groups are trained over 12 weeks.
3) Increase the number of sets per muscle group per session gradually as tolerated.

Neuromotor training:

1) Increase exercise intensity by performing more challenging or advanced balances, postures, or movements over 12 weeks.

Flexibility training:

1) Increase the number of days per week of stretching over 12 weeks.

Contact Details for the UConn Graduate Research Assistant

Alexander Wright

Email: Alexander.Wright@hhchealth.org

Phone: (860) 486-6814

12 Week Exercise Program Progression Guidance

<table>
<tr><th></th><th>Aerobic</th><th>Resistance</th><th>Neuromotor</th><th>Flexibility</th></tr>
<tr><td>Week 1</td><td rowspan="2">3-4 days per week at light to moderate intensity for ~30 minutes</td><td rowspan="3">1 day per week at moderate intensity for ~30 minutes</td><td rowspan="4">1 day per week at light to moderate intensity for 20-30 minutes</td><td rowspan="6">2 days per week for 5 minutes</td></tr>
<tr><td>Week 2</td></tr>
<tr><td>Week 3</td><td rowspan="2">3-4 days per week at light to moderate intensity for 30-40 minutes</td></tr>
<tr><td>Week 4</td><td rowspan="3">2 days per week at moderate intensity for ~30 minutes</td></tr>
<tr><td>Week 5</td><td rowspan="2">3-4 days per week at moderate intensity for 30-50 minutes</td><td rowspan="8">2 days per week at moderate intensity for 20-30 minutes</td></tr>
<tr><td>Week 6</td></tr>
<tr><td>Week 7</td><td rowspan="3">4-5 days per week at moderate intensity for 30-50 minutes and/or vigorous intensity for 15-25 minutes</td><td rowspan="6">2-3 days per week at moderate to vigorous intensity for ~45 minutes</td><td rowspan="6">≥2-3 days per week for 5 minutes</td></tr>
<tr><td>Week 8</td></tr>
<tr><td>Week 9</td></tr>
<tr><td>Week 10</td><td rowspan="3">≥5 days per week at moderate intensity for 30-60 minutes and/or vigorous intensity for 15-30 minutes</td></tr>
<tr><td>Week 11</td></tr>
<tr><td>Week 12</td></tr>
</table>

Week 1

Aerobic exercise: 3-4 days per week for ~30 minutes* per day at light to moderate intensity, to total 90 minutes per week. **Can be accumulated in multiple 10+ minute bouts over the day.*

Resistance exercise: 1 day per week for ~30 minutes per session at moderate intensity.

Neuromotor exercise: 1 day per week for 20-30 minutes per session at light to moderate intensity.

Flexibility exercise: 2 days per week for ~5 minutes per session*, stretching to the point of tightness or slight discomfort. **Can be included in warm-ups or cool-downs.*

Aerobic Exercise Workout Examples at Light to Moderate Intensity Week 1.

Component	Time	Type	Examples
1) Warm-up	5 minutes	Aerobic activity or dynamic stretching	- Walking at a talking pace on a treadmill<br>- Walking to the gym<br>- Dynamic stretching (3-6 sets of 30-90 sec with 15 sec rest periods between sets):<br>• Marching in place<br>• Walking lunges<br>• Hip circles or openers<br>• Arm swings/arm circles<br>• Torso twists
2) Aerobic Exercise	~30 minutes	Steady state moderate intensity aerobic activity	- Walking at a pace that increases your breathing rate noticeably<br>- Peddling slowly on a stationary bicycle or leisurely cycling<br>- Peddling slowly on an elliptical machine<br>- Rowing slowly on a machine<br>- Slow dancing<br>- Aqua-aerobics
3) Cool-down	5 minutes	Static flexibility OR slowly reduce the pace of aerobic exercise	- Static stretching (10-30 seconds per stretch, 2-4 repetitions of each exercise):<br>• Standing calf stretch<br>• Quadricep stretch<br>• Kneeling hip flexor stretch (iliopsoas)<br>• Seated hamstring stretch<br>• Child's pose stretch<br>• Chest stretch<br>• Cross-body shoulder stretch<br>• Neck lateral flexion stretch

Resistance Exercise Workout Examples at Moderate Intensity Week 1.

Component	Time	Type	Example exercises with machines, free weights, bodyweight, or resistance bands
Resistance Exercise (Day 1)	~30 minutes	Resistance training for all major muscle groups	2-3 sets of 8-12 repetitions (rest 1.5-2 minutes between sets)<br>RPE 12-13; 1-RM: 50-69%<br>• Chest press<br>• Seated row<br>• Leg press<br>• Hip bridges<br>• Leg curls<br>• Arm curls<br>• Abdominal flexions<br>• Planks
Resistance Exercise (Day 2)	~30 minutes	Resistance training for all major muscle groups	2-3 sets of 8-12 repetitions (rest 1.5-2 minutes between sets):<br>RPE 12-13; 1-RM: 50-69%<br>• Shoulder press<br>• Lat pull-downs<br>• Hip kickbacks<br>• Hip abductions<br>• Leg extensions<br>• Triceps extensions<br>• Abdominal twists<br>• Side planks

Neuromotor Exercise Workout Examples at Light to Moderate Intensity Week 1.

Component	Time	Type	Examples
Neuromotor Exercise	20-30 minutes	Training that involves motor skills such as balance, coordination, agility	- Yoga (Hatha sitting/Vinyasa/ Nadisodhana)<br>- Tai chi (qi gong, sitting)<br>- Pilates (traditional)<br>- Balance exercise (15-30 seconds per hold, 5-6 cycles):<br>• two-legged stance<br>• semi-tandem stance<br>• reaching from a narrow stance<br>• tandem walking<br>• standing with eyes closed

Week 2

Aerobic exercise: 3-4 days per week for ~30 minutes* per day at light to moderate intensity, to total 90 minutes per week. **Can be accumulated in 10+ minute bouts or sessions over the day.*

Resistance exercise: 1 day per week for ~30 minutes per session at moderate intensity.

Neuromotor exercise: 1 day per week for 20-30 minutes per session at light to moderate intensity.

Flexibility exercise: 2 days per week for ~5 minutes per session*, stretching to the point of tightness or slight discomfort. **Can be implemented into warm-ups or cool-downs.*

Aerobic Exercise Workout Examples at Light to Moderate Intensity Week 2.

Component	Time	Type	Examples
1) Warm-up	5 minutes	Aerobic activity or dynamic stretching	- Walking at a talking pace on a treadmill<br>- Walking to the gym<br>- Dynamic stretching (3-6 sets of 30-90 sec with 15 sec rest periods between sets):<br>• Marching in place<br>• Walking lunges<br>• Hip circles or openers<br>• Arm swings/arm circles<br>• Torso twists
2) Aerobic Exercise	~30 minutes	Steady state moderate intensity aerobic activity	- Walking at a pace that increases your breathing rate noticeably<br>- Peddling slowly on a stationary bicycle or leisurely cycling<br>- Peddling slowly on an elliptical machine<br>- Rowing slowly on a machine<br>- Slow dancing<br>- Aqua-aerobics
3) Cool-down	5 minutes	Static flexibility OR slowly reduce the pace of aerobic exercise	- Static stretching (10-30 seconds per stretch, 2-4 repetitions of each exercise):<br>• Standing calf stretch<br>• Quadricep stretch<br>• Kneeling hip flexor stretch (iliopsoas)<br>• Seated hamstring stretch<br>• Child's pose stretch<br>• Chest stretch<br>• Cross-body shoulder stretch<br>• Neck lateral flexion stretch

Resistance Exercise Workout Examples at Moderate Intensity Week 2.

Component	Time	Type	Example exercises with machines, free weights, bodyweight, or resistance bands
Resistance Exercise (Day 1)	~30 minutes	Resistance training for all major muscle groups	2-3 sets of 8-12 repetitions (rest 1.5-2 minutes between sets)<br>RPE 12-13; 1-RM: 50-69%<br>• Chest press<br>• Seated row<br>• Leg press<br>• Hip bridges<br>• Leg curls<br>• Arm curls<br>• Abdominal flexions<br>• Planks
Resistance Exercise (Day 2)	~30 minutes	Resistance training for all major muscle groups	2-3 sets of 8-12 repetitions (rest 1.5-2 minutes between sets)<br>RPE 12-13; 1-RM: 50-69%<br>• Shoulder press<br>• Lat pull-downs<br>• Hip kickbacks<br>• Hip abductions<br>• Leg extensions<br>• Triceps extensions<br>• Abdominal twists<br>• Side planks

Neuromotor Exercise Workout Examples at Light to Moderate Intensity Week 2.

Component	Time	Type	Examples
Neuromotor Exercise	20-30 minutes	Training that involves motor skills such as balance, coordination, agility	- Yoga (Hatha sitting/Vinyasa/ Nadisodhana)<br>- Tai chi (qi gong, sitting)<br>- Pilates (traditional)<br>- Balance exercise (15-30 seconds per hold, 5-6 cycles):<br>• two-legged stance<br>• semi-tandem stance<br>• reaching from a narrow stance<br>• tandem walking<br>• standing with eyes closed

Week 3

Aerobic exercise: 3-4 days per week for 30-40 minutes* per day at light to moderate intensity, to total 125 minutes per week. **Can be accumulated in 10+ minute bouts or sessions over the day.*

Resistance exercise: 1 day per week for ~30 minutes per session at moderate intensity.

Neuromotor exercise: 1 day per week for 20-30 minutes per session at light to moderate intensity.

Flexibility exercise: 2 days per week for ~5 minutes per session*, stretching to the point of tightness or slight discomfort. **Can be implemented into warm-ups or cool-downs.*

Aerobic Exercise Workout Examples at Light to Moderate Intensity Week 3.

Component	Time	Type	Examples
1) Warm-up	5 minutes	Aerobic activity or dynamic stretching	- Walking at a talking pace on a treadmill<br>- Walking to the gym<br>- Dynamic stretching (3-6 sets of 30-90 sec with 15 sec rest periods between sets):<br>• Marching in place<br>• Walking lunges<br>• Hip circles or openers<br>• Arm swings/arm circles<br>• Torso twists
2) Aerobic Exercise	30-40 minutes	Steady state moderate intensity aerobic activity	- Walking at a pace that increases your breathing rate noticeably<br>- Peddling slowly on a stationary bicycle or leisurely cycling<br>- Peddling slowly on an elliptical machine<br>- Rowing slowly on a machine<br>- Slow dancing<br>- Aqua-aerobics
3) Cool-down	5 minutes	Static flexibility OR slowly reduce the pace of aerobic exercise	- Static stretching (10-30 seconds per stretch, 2-4 repetitions of each exercise):<br>• Standing calf stretch<br>• Quadricep stretch<br>• Kneeling hip flexor stretch (iliopsoas)<br>• Seated hamstring stretch<br>• Child’s pose stretch<br>• Chest stretch<br>• Cross-body shoulder stretch<br>• Neck lateral flexion stretch

Resistance Exercise Workout Examples at Moderate Intensity Week 3.

Component	Time	Type	Example exercises with machines, free weights, bodyweight, or resistance bands
Resistance Exercise (Day 1)	~30 minutes	Resistance training for all major muscle groups	2-3 sets of 8-12 repetitions (rest 1.5-2 minutes between sets)<br>RPE 12-13; 1-RM: 50-69%<br>• Chest press<br>• Seated row<br>• Leg press<br>• Hip bridges<br>• Leg curls<br>• Arm curls<br>• Abdominal flexions<br>• Planks
Resistance Exercise (Day 2)	~30 minutes	Resistance training for all major muscle groups	2-3 sets of 8-12 repetitions (rest 1.5-2 minutes between sets)<br>RPE 12-13; 1-RM: 50-69%<br>• Shoulder press<br>• Lat pull-downs<br>• Hip kickbacks<br>• Hip abductions<br>• Leg extensions<br>• Triceps extensions<br>• Abdominal twists<br>• Side planks

Neuromotor Exercise Workout Examples at Light to Moderate Intensity Week 3.

Component	Time	Type	Examples
Neuromotor Exercise	20-30 minutes	Training that involves motor skills such as balance, coordination, agility	- Yoga (Hatha sitting/Vinyasa/ Nadisodhana)<br>- Tai chi (qi gong, sitting)<br>- Pilates (traditional)<br>- Balance exercise (15-30 seconds per hold, 5-6 cycles):<br>• two-legged stance<br>• semi-tandem stance<br>• reaching from a narrow stance<br>• tandem walking<br>• standing with eyes closed

Week 4

Aerobic exercise: 3-4 days per week for 30-40 minutes* per day at moderate intensity, to total 125 minutes per week. **Can be accumulated in 10+ minute bouts or sessions over the day.*

Resistance exercise: 2 days per week for ~30 minutes per session at moderate intensity.

Neuromotor exercise: 1 day per week for 20-30 minutes per session at light to moderate intensity.

Flexibility exercise: 2 days per week for ~5 minutes per session*, stretching to the point of tightness or slight discomfort. **Can be implemented into warm-ups or cool-downs.*

Aerobic Exercise Workout Examples at Moderate Intensity Week 4.

Component	Time	Type	Examples
1) Warm-up	5 minutes	Aerobic activity or dynamic stretching	- Walking at a talking pace on a treadmill<br>- Walking to the gym<br>- Dynamic stretching (3-6 sets of 30-90 sec with 15 sec rest periods between sets):<br>• Marching in place<br>• Walking lunges<br>• Hip circles or openers<br>• Arm swings/arm circles<br>• Torso twists
2) Aerobic Exercise	30-40 minutes	Steady state moderate intensity aerobic activity	- Walking at a pace that increases your breathing rate noticeably<br>- Peddling slowly on a stationary bicycle or leisurely cycling<br>- Peddling slowly on an elliptical machine<br>- Rowing slowly on a machine<br>- Slow dancing<br>- Aqua-aerobics
3) Cool-down	5 minutes	Static flexibility OR slowly reduce the pace of aerobic exercise	- Static stretching (10-30 seconds per stretch, 2-4 repetitions of each exercise):<br>• Standing calf stretch<br>• Quadricep stretch<br>• Kneeling hip flexor stretch (iliopsoas)<br>• Seated hamstring stretch<br>• Child's pose stretch<br>• Chest stretch<br>• Cross-body shoulder stretch<br>• Neck lateral flexion stretch

Resistance Exercise Workout Examples at Moderate Intensity Week 4.

Component	Time	Type	Example exercises with machines, free weights, bodyweight, or resistance bands
Resistance Exercise (Day 1)	~30 minutes	Resistance training for all major muscle groups	2-3 sets of 8-12 repetitions (rest 1.5-2 minutes between sets)<br>RPE 12-13; 1-RM: 50-69%<br>• Chest press<br>• Seated row<br>• Leg press<br>• Hip bridges<br>• Leg curls<br>• Arm curls<br>• Abdominal flexions<br>• Planks
Resistance Exercise (Day 2)	~30 minutes	Resistance training for all major muscle groups	2-3 sets of 8-12 repetitions (rest 1.5-2 minutes between sets)<br>RPE 12-13; 1-RM: 50-69%<br>• Shoulder press<br>• Lat pull-downs<br>• Hip kickbacks<br>• Hip abductions<br>• Leg extensions<br>• Triceps extensions<br>• Abdominal twists<br>• Side planks

Neuromotor Exercise Workout Examples at Light to Moderate Intensity Week 4.

Component	Time	Type	Examples
Neuromotor Exercise	20-30 minutes	Training that involves motor skills such as balance, coordination, agility	- Yoga (Hatha sitting/Vinyasa/ Nadisodhana)<br>- Tai chi (qi gong, sitting)<br>- Pilates (traditional)<br>- Balance exercise (15-30 seconds per hold, 5-6 cycles):<br>• two-legged stance<br>• semi-tandem stance<br>• reaching from a narrow stance<br>• tandem walking<br>• standing with eyes closed

Week 5

Aerobic exercise: 3-4 days per week for 30-50 minutes* per day at moderate intensity, to total 150 minutes per week. **Can be accumulated in 10+ minute bouts or sessions over the day.*

Resistance exercise: 2 days per week for ~30 minutes per session at moderate intensity.

Neuromotor exercise: 2 days per week for 20-30 minutes per session at moderate intensity.

Flexibility exercise: 2 days per week for ~5 minutes per session*, stretching to the point of tightness or slight discomfort. **Can be implemented into warm-ups or cool-downs.*

Aerobic Exercise Workout Examples at Moderate Intensity Week 5.

Component	Time	Type	Examples
1) Warm-up	5 minutes	Aerobic activity or dynamic stretching	- Walking at a talking pace on a treadmill<br>- Walking to the gym<br>- Dynamic stretching (3-6 sets of 30-90 sec with 15 sec rest periods between sets):<br>• Marching in place<br>• Walking lunges<br>• Hip circles or openers<br>• Arm swings/arm circles<br>• Torso twists
2) Aerobic Exercise	30-50 minutes	Steady state moderate intensity aerobic activity	- Walking at a pace that increases your breathing rate noticeably<br>- Peddling slowly on a stationary bicycle or leisurely cycling<br>- Peddling slowly on an elliptical machine<br>- Rowing slowly on a machine<br>- Slow dancing<br>- Aqua-aerobics
3) Cool-down	5 minutes	Static flexibility OR slowly reduce the pace of aerobic exercise	- Static stretching (10-30 seconds per stretch, 2-4 repetitions of each exercise):<br>• Standing calf stretch<br>• Quadricep stretch<br>• Kneeling hip flexor stretch (iliopsoas)<br>• Seated hamstring stretch<br>• Child's pose stretch<br>• Chest stretch<br>• Cross-body shoulder stretch<br>• Neck lateral flexion stretch

Resistance Exercise Workout Examples at Moderate Intensity Week 5.

Component	Time	Type	Example exercises with machines, free weights, bodyweight, or resistance bands
Resistance Exercise (Day 1)	~30 minutes	Resistance training for all major muscle groups	2-3 sets of 8-12 repetitions (rest 1.5-2 minutes between sets)<br>RPE 12-13; 1-RM: 50-69%<br>• Chest press<br>• Seated row<br>• Leg press<br>• Hip bridges<br>• Leg curls<br>• Arm curls<br>• Abdominal flexions<br>• Planks
Resistance Exercise (Day 2)	~30 minutes	Resistance training for all major muscle groups	2-3 sets of 8-12 repetitions (rest 1.5-2 minutes between sets)<br>RPE 12-13; 1-RM: 50-69%<br>• Shoulder press<br>• Lat pull-downs<br>• Hip kickbacks<br>• Hip abductions<br>• Leg extensions<br>• Triceps extensions<br>• Abdominal twists<br>• Side planks

Neuromotor Exercise Workout Examples at Light to Moderate Intensity Week 5.

Component	Time	Type	Examples
Neuromotor Exercise	20-30 minutes	Training that involves motor skills such as balance, coordination, agility	- Yoga (Hatha sitting/Vinyasa/ Nadisodhana)<br>- Tai chi (qi gong, sitting)<br>- Pilates (traditional)<br>- Balance exercise (15-30 seconds per hold, 5-6 cycles):<br>• two-legged stance<br>• semi-tandem stance<br>• reaching from a narrow stance<br>• tandem walking<br>• standing with eyes closed

Week 6

Aerobic exercise: 3-4 days per week for 30-50 minutes* per day at moderate intensity, to total 150 minutes per week. **Can be accumulated in 10+ minute bouts or sessions over the day.*

Resistance exercise: 2 days per week for ~30 minutes per session at moderate intensity.

Neuromotor exercise: 2 days per week for 20-30 minutes per session at moderate intensity.

Flexibility exercise: 2 days per week for ~5 minutes per session*, stretching to the point of tightness or slight discomfort. **Can be implemented into warm-ups or cool-downs.*

Aerobic Exercise Workout Examples at Moderate Intensity Week 6.

Component	Time	Type	Examples
1) Warm-up	5 minutes	Aerobic activity or dynamic stretching	- Walking at a talking pace on a treadmill<br>- Walking to the gym<br>- Dynamic stretching (3-6 sets of 30-90 sec with 15 sec rest periods between sets):<br>• Marching in place<br>• Walking lunges<br>• Hip circles or openers<br>• Arm swings/arm circles<br>• Torso twists
2) Aerobic Exercise	30-50 minutes	Steady state moderate intensity aerobic activity	- Walking at a pace that increases your breathing rate noticeably<br>- Peddling slowly on a stationary bicycle or leisurely cycling<br>- Peddling slowly on an elliptical machine<br>- Rowing slowly on a machine<br>- Slow dancing<br>- Aqua-aerobics
3) Cool-down	5 minutes	Static flexibility OR slowly reduce the pace of aerobic exercise	- Static stretching (10-30 seconds per stretch, 2-4 repetitions of each exercise):<br>• Standing calf stretch<br>• Quadricep stretch<br>• Kneeling hip flexor stretch (iliopsoas)<br>• Seated hamstring stretch<br>• Child's pose stretch<br>• Chest stretch<br>• Cross-body shoulder stretch<br>• Neck lateral flexion stretch

Resistance Exercise Workout Examples at Moderate Intensity Week 6.

Component	Time	Type	Example exercises with machines, free weights, bodyweight, or resistance bands
Resistance Exercise (Day 1)	~30 minutes	Resistance training for all major muscle groups	2-3 sets of 8-12 repetitions (rest 1.5-2 minutes between sets)<br>RPE 12-13; 1-RM: 50-69%<br>• Chest press<br>• Seated row<br>• Leg press<br>• Hip bridges<br>• Leg curls<br>• Arm curls<br>• Abdominal flexions<br>• Planks
Resistance Exercise (Day 2)	~30 minutes	Resistance training for all major muscle groups	2-3 sets of 8-12 repetitions (rest 1.5-2 minutes between sets)<br>RPE 12-13; 1-RM: 50-69%<br>• Shoulder press<br>• Lat pull-downs<br>• Hip kickbacks<br>• Leg Extensions<br>• Triceps extensions<br>• Abdominal twists<br>• Side planks

Neuromotor Exercise Workout Examples at Light to Moderate Intensity Week 6.

Component	Time	Type	Examples
Neuromotor Exercise	20-30 minutes	Training that involves motor skills such as balance, coordination, agility	- Yoga (Hatha sitting/Vinyasa/ Nadisodhana)<br>- Tai chi (qi gong, sitting)<br>- Pilates (traditional)<br>- Balance exercise (15-30 seconds per hold, 5-6 cycles):<br>• two-legged stance<br>• semi-tandem stance<br>• reaching from a narrow stance<br>• tandem walking<br>• standing with eyes closed

Week 7

Aerobic exercise: 4-5 days per week for 30-50 minutes* per day at moderate intensity or 15-25 minutes* per day at vigorous intensity (or a combination), to total 200 minutes per week. **Can be accumulated in 10+ minute bouts or sessions over the day.*

Resistance exercise: 2-3 days per week for ~45 minutes per session at moderate to vigorous intensity.

Neuromotor exercise: 2 days per week for 20-30 minutes per session at moderate intensity.

Flexibility exercise: ≥2-3 days per week for ~5 minutes per session*, stretching to the point of tightness or slight discomfort. **Can be implemented into warm-ups or cool-downs.*

Aerobic Exercise Workout Examples at Moderate Intensity Week 7.

Component	Time	Type	Examples
1) Warm-up	5 minutes	Aerobic activity or dynamic stretching	- Walking at a talking pace on a treadmill<br>- Walking to the gym<br>- Dynamic stretching (3-6 sets of 30-90 sec with 15 sec rest periods between sets):<br>• Marching in place<br>• Walking lunges<br>• Hip circles or openers<br>• Arm swings/arm circles<br>• Torso twists
2) Aerobic Exercise	30-50 minutes<br><br><br><br><br><br><br>OR<br><br>15-25 minutes	Steady state moderate intensity aerobic activity<br><br><br><br><br>OR<br><br>Steady state vigorous intensity aerobic activity or high intensity interval training	- Walking at a pace that increases your breathing rate noticeably<br>- Peddling slowly on a stationary bicycle or leisurely cycling<br>- Peddling slowly on an elliptical machine<br>- Rowing slowly on a machine<br>- Slow dancing<br>- Aqua-aerobics<br><br>- Walking very briskly, jogging, or running<br>- Peddling fast on a stationary bicycle or cycling fast or uphill<br>- Peddling fast on an elliptical machine<br>- Rowing fast on a machine<br>- Stepping on the stair stepper machine<br>- Fast dancing<br>- Aerobics
3) Cool-down	5 minutes	Static flexibility OR slowly reduce the pace of aerobic exercise	- Static stretching (10-30 seconds per stretch, 2-4 repetitions of each exercise):<br>• Standing calf stretch<br>• Quadricep stretch<br>• Kneeling hip flexor stretch (iliopsoas)<br>• Seated hamstring stretch<br>• Child's pose stretch

			• Chest stretch<br>• Cross-body shoulder stretch<br>• Neck lateral flexion stretch

Resistance Exercise Workout Examples at Moderate to Vigorous Intensity Week 7.

Component	Time	Type	Example exercises with machines, free weights, bodyweight, or resistance bands
Resistance Exercise (Day 1)	~45 minutes	Resistance training for all major muscle groups	3-4 sets of 8-12 repetitions (rest 1.5-2.5 minutes between sets)<br>RPE 12-13 to 14-17; 1-RM: 50-85%<br>• Chest press<br>• Seated row<br>• Leg press<br>• Hip bridges<br>• Leg curls<br>• Arm curls<br>• Abdominal flexions<br>• Planks
Resistance Exercise (Day 2)	~45 minutes	Resistance training for all major muscle groups	3-4 sets of 8-12 repetitions (rest 1.5-2.5 minutes between sets)<br>RPE 12-13 to 14-17; 1-RM: 50-85%<br>• Shoulder press<br>• Lat pull-downs<br>• Hip kickbacks<br>• Hip abductions<br>• Leg extensions<br>• Triceps extensions<br>• Abdominal twists<br>• Side planks
Resistance Exercise (Day 3)	~45 minutes	Resistance training for all major muscle groups	3-4 sets of 8-12 repetitions (rest 1.5-2.5 minutes between sets)<br>RPE 12-13 to 14-17; 1-RM: 50-85%)<br>• Pick 6-10 exercises from above to train major muscle groups

Neuromotor Exercise Workout Examples at Moderate Intensity Week 7.

Component	Time	Type	Examples
Neuromotor Exercise	20-30 minutes	Training that involves motor skills such as balance, coordination, agility	- Yoga (power/Surya Namaskar)<br>- Tai chi (qi gong standing/yang style)<br>- Pilates (POUND® with drumming)<br>- Single leg or Bosu ball exercises<br>- Balance exercises (15-30 seconds per hold, 5-6 cycles):<br>• tandem stance<br>• one-legged stance<br>• stepping over obstacles<br>• heel or toe walks<br>• walking while turning the head

Week 8

Aerobic exercise: 4-5 days per week for 30-50 minutes* per day at moderate intensity or 15-25 minutes* per day at vigorous intensity (or a combination), to total 200 minutes per week. **Can be accumulated in 10+ minute bouts or sessions over the day.*

Resistance exercise: 2-3 days per week for ~45 minutes per session at moderate to vigorous intensity.

Neuromotor exercise: 2 days per week for 20-30 minutes per session at moderate intensity.

Flexibility exercise: ≥2-3 days per week for ~5 minutes per session*, stretching to the point of tightness or slight discomfort. **Can be implemented into warm-ups or cool-downs.*

Aerobic Exercise Workout Examples at Moderate Intensity Week 8.

Component	Time	Type	Examples
1) Warm-up	5 minutes	Aerobic activity or dynamic stretching	- Walking at a talking pace on a treadmill<br>- Walking to the gym<br>- Dynamic stretching (3-6 sets of 30-90 sec with 15 sec rest periods between sets):<br>• Marching in place<br>• Walking lunges<br>• Hip circles or openers<br>• Arm swings/arm circles<br>• Torso twists
2) Aerobic Exercise	30-50 minutes<br><br><br><br><br><br><br>OR<br><br>15-25 minutes	Steady state moderate intensity aerobic activity<br><br><br><br>OR<br><br>Steady state vigorous intensity aerobic activity or high intensity interval training	- Walking at a pace that increases your breathing rate noticeably<br>- Peddling slowly on a stationary bicycle or leisurely cycling<br>- Peddling slowly on an elliptical machine<br>- Rowing slowly on a machine<br>- Slow dancing<br>- Aqua-aerobics<br><br>- Walking very briskly, jogging, or running<br>- Peddling fast on a stationary bicycle or cycling fast or uphill<br>- Peddling fast on an elliptical machine<br>- Rowing fast on a machine<br>- Stepping on the stair stepper machine<br>- Fast dancing<br>- Aerobics
3) Cool-down	5 minutes	Static flexibility OR slowly reduce the pace of aerobic exercise	- Static stretching (10-30 seconds per stretch, 2-4 repetitions of each exercise):<br>• Standing calf stretch<br>• Quadricep stretch<br>• Kneeling hip flexor stretch (iliopsoas)<br>• Seated hamstring stretch<br>• Child's pose stretch

			• Chest stretch<br>• Cross-body shoulder stretch<br>• Neck lateral flexion stretch

Resistance Exercise Workout Examples at Moderate to Vigorous Intensity Week 8.

Component	Time	Type	Example exercises with machines, free weights, bodyweight, or resistance bands
Resistance Exercise (Day 1)	~45 minutes	Resistance training for all major muscle groups	3-4 sets of 8-12 repetitions (rest 1.5-2.5 minutes between sets)<br>RPE 12-13 to 14-17; 1-RM: 50-85%<br>• Chest press<br>• Seated row<br>• Leg press<br>• Hip bridges<br>• Leg curls<br>• Arm curls<br>• Abdominal flexions<br>• Planks
Resistance Exercise (Day 2)	~45 minutes	Resistance training for all major muscle groups	3-4 sets of 8-12 repetitions (rest 1.5-2.5 minutes between sets)<br>RPE 12-13 to 14-17; 1-RM: 50-85%<br>• Shoulder press<br>• Lat pull-downs<br>• Hip kickbacks<br>• Hip abductions<br>• Leg extensions<br>• Triceps extensions<br>• Abdominal twists<br>• Side planks
Resistance Exercise (Day 3)	~45 minutes	Resistance training for all major muscle groups	3-4 sets of 8-12 repetitions (rest 1.5-2.5 minutes between sets)<br>RPE 12-13 to 14-17; 1-RM: 50-85%)<br>• Pick 6-10 exercises from above to train major muscle groups

Neuromotor Exercise Workout Examples at Moderate Intensity Week 8.

Component	Time	Type	Examples
Neuromotor Exercise	20-30 minutes	Training that involves motor skills such as balance, coordination, agility	- Yoga (power/Surya Namaskar)<br>- Tai chi (qi gong standing/yang style)<br>- Pilates (POUND® with drumming)<br>- Single leg or Bosu ball exercises<br>- Balance exercises (15-30 seconds per hold, 5-6 cycles):<br>• tandem stance<br>• one-legged stance<br>• stepping over obstacles<br>• heel or toe walks<br>• walking while turning the head

Week 9

Aerobic exercise: 4-5 days per week for 30-50 minutes* per day at moderate intensity or 15-25 minutes* per day at vigorous intensity (or a combination), to total 200 minutes per week. **Can be accumulated in 10+ minute bouts or sessions over the day.*

Resistance exercise: 2-3 days per week for ~45 minutes per session at moderate to vigorous intensity.

Neuromotor exercise: 2 days per week for 20-30 minutes per session at moderate intensity.

Flexibility exercise: ≥2-3 days per week for ~5 minutes per session*, stretching to the point of tightness or slight discomfort. **Can be implemented into warm-ups or cool-downs.*

Aerobic Exercise Workout Examples at Moderate Intensity Week 9.

Component	Time	Type	Examples
1) Warm-up	5 minutes	Aerobic activity or dynamic stretching	- Walking at a talking pace on a treadmill<br>- Walking to the gym<br>- Dynamic stretching (3-6 sets of 30-90 sec with 15 sec rest periods between sets):<br>• Marching in place<br>• Walking lunges<br>• Hip circles or openers<br>• Arm swings/arm circles<br>• Torso twists
2) Aerobic Exercise	30-50 minutes<br><br>OR<br><br>15-25 minutes	Steady state moderate intensity aerobic activity<br><br>OR<br><br>Steady state vigorous intensity aerobic activity or high intensity interval training	- Walking at a pace that increases your breathing rate noticeably<br>- Peddling slowly on a stationary bicycle or leisurely cycling<br>- Peddling slowly on an elliptical machine<br>- Rowing slowly on a machine<br>- Slow dancing<br>- Aqua-aerobics<br><br>- Walking very briskly, jogging, or running<br>- Peddling fast on a stationary bicycle or cycling fast or uphill<br>- Peddling fast on an elliptical machine<br>- Rowing fast on a machine<br>- Stepping on the stair stepper machine<br>- Fast dancing<br>- Aerobics
3) Cool-down	5 minutes	Static flexibility OR slowly reduce the pace of aerobic exercise	- Static stretching (10-30 seconds per stretch, 2-4 repetitions of each exercise):<br>• Standing calf stretch<br>• Quadricep stretch<br>• Kneeling hip flexor stretch (iliopsoas)<br>• Seated hamstring stretch<br>• Child’s pose stretch

			• Chest stretch<br>• Cross-body shoulder stretch<br>• Neck lateral flexion stretch

Resistance Exercise Workout Examples at Moderate to Vigorous Intensity Week 9.

Component	Time	Type	Example exercises with machines, free weights, bodyweight, or resistance bands
Resistance Exercise (Day 1)	~45 minutes	Resistance training for all major muscle groups	3-4 sets of 8-12 repetitions (rest 1.5-2.5 minutes between sets)<br>RPE 12-13 to 14-17; 1-RM: 50-85%<br>• Chest press<br>• Seated row<br>• Leg press<br>• Hip bridges<br>• Leg curls<br>• Arm curls<br>• Abdominal flexions<br>• Planks
Resistance Exercise (Day 2)	~45 minutes	Resistance training for all major muscle groups	3-4 sets of 8-12 repetitions (rest 1.5-2.5 minutes between sets)<br>RPE 12-13 to 14-17; 1-RM: 50-85%<br>• Shoulder press<br>• Lat pull-downs<br>• Hip kickbacks<br>• Hip abductions<br>• Leg extensions<br>• Triceps extensions<br>• Abdominal twists<br>• Side planks
Resistance Exercise (Day 3)	~45 minutes	Resistance training for all major muscle groups	3-4 sets of 8-12 repetitions (rest 1.5-2.5 minutes between sets)<br>RPE 12-13 to 14-17; 1-RM: 50-85%)<br>• Pick 6-10 exercises from above to train major muscle groups

Neuromotor Exercise Workout Examples at Moderate Intensity Week 9.

Component	Time	Type	Examples
Neuromotor Exercise	20-30 minutes	Training that involves motor skills such as balance, coordination, agility	- Yoga (power/Surya Namaskar)<br>- Tai chi (qi gong standing/yang style)<br>- Pilates (POUND® with drumming)<br>- Single leg or Bosu ball exercises<br>- Balance exercises (15-30 seconds per hold, 5-6 cycles):<br>• tandem stance<br>• one-legged stance<br>• stepping over obstacles<br>• heel or toe walks<br>• walking while turning the head

Week 10

Aerobic exercise: ≥5 days per week for 30-60 minutes* per day at moderate intensity or 15-30 minutes* per day at vigorous intensity (or a combination), to total 250-300 minutes per week. **Can be accumulated in 10+ minute bouts or sessions over the day.*

Resistance exercise: 2-3 days per week for ~45 minutes per session at moderate to vigorous intensity.

Neuromotor exercise: 2 days per week for 20-30 minutes per session at moderate intensity.

Flexibility exercise: ≥2-3 days per week for ~5 minutes per session*, stretching to the point of tightness or slight discomfort. **Can be implemented into warm-ups or cool-downs.*

Aerobic Exercise Workout Examples at Moderate Intensity Week 10.

Component	Time	Type	Examples
1) Warm-up	5 minutes	Aerobic activity or dynamic stretching	- Walking at a talking pace on a treadmill<br>- Walking to the gym<br>- Dynamic stretching (3-6 sets of 30-90 sec with 15 sec rest periods between sets):<br>• Marching in place<br>• Walking lunges<br>• Hip circles or openers<br>• Arm swings/arm circles<br>• Torso twists
2) Aerobic Exercise	30-60 minutes<br><br>OR<br><br>15-30 minutes	Steady state moderate intensity aerobic activity<br><br>OR<br><br>Steady state vigorous intensity aerobic activity or high intensity interval training	- Walking at a pace that increases your breathing rate noticeably<br>- Peddling slowly on a stationary bicycle or leisurely cycling<br>- Peddling slowly on an elliptical machine<br>- Rowing slowly on a machine<br>- Slow dancing<br>- Aqua-aerobics<br><br>- Walking very briskly, jogging, or running<br>- Peddling fast on a stationary bicycle or cycling fast or uphill<br>- Peddling fast on an elliptical machine<br>- Rowing fast on a machine<br>- Stepping on the stair stepper machine<br>- Fast dancing<br>- Aerobics
3) Cool-down	5 minutes	Static flexibility OR slowly reduce the pace of aerobic exercise	- Static stretching (10-30 seconds per stretch, 2-4 repetitions of each exercise):<br>• Standing calf stretch<br>• Quadricep stretch<br>• Kneeling hip flexor stretch (iliopsoas)<br>• Seated hamstring stretch<br>• Child's pose stretch

			• Chest stretch<br>• Cross-body shoulder stretch<br>• Neck lateral flexion stretch

Resistance Exercise Workout Examples at Moderate to Vigorous Intensity Week 10.

Component	Time	Type	Example exercises with machines, free weights, bodyweight, or resistance bands
Resistance Exercise (Day 1)	~45 minutes	Resistance training for all major muscle groups	3-4 sets of 8-12 repetitions (rest 1.5-2.5 minutes between sets)<br>RPE 12-13 to 14-17; 1-RM: 50-85%<br>• Chest press<br>• Seated row<br>• Leg press<br>• Hip bridges<br>• Leg curls<br>• Arm curls<br>• Abdominal flexions<br>• Planks
Resistance Exercise (Day 2)	~45 minutes	Resistance training for all major muscle groups	3-4 sets of 8-12 repetitions (rest 1.5-2.5 minutes between sets)<br>RPE 12-13 to 14-17; 1-RM: 50-85%<br>• Shoulder press<br>• Lat pull-downs<br>• Hip kickbacks<br>• Hip abductions<br>• Leg extensions<br>• Triceps extensions<br>• Abdominal twists<br>• Side planks
Resistance Exercise (Day 3)	~45 minutes	Resistance training for all major muscle groups	3-4 sets of 8-12 repetitions (rest 1.5-2.5 minutes between sets)<br>RPE 12-13 to 14-17; 1-RM: 50-85%)<br>• Pick 6-10 exercises from above to train major muscle groups

Neuromotor Exercise Workout Examples at Moderate Intensity Week 10.

Component	Time	Type	Examples
Neuromotor Exercise	20-30 minutes	Training that involves motor skills such as balance, coordination, agility	- Yoga (power/Surya Namaskar)<br>- Tai chi (qi gong standing/yang style)<br>- Pilates (POUND® with drumming)<br>- Single leg or Bosu ball exercises<br>- Balance exercises (15-30 seconds per hold, 5-6 cycles):<br>• tandem stance<br>• one-legged stance<br>• stepping over obstacles<br>• heel or toe walks<br>• walking while turning the head

Week 11

Aerobic exercise: ≥5 days per week for 30-60 minutes* per day at moderate intensity or 15-30 minutes* per day at vigorous intensity (or a combination), to total 250-300 minutes per week. **Can be accumulated in 10+ minute bouts or sessions over the day.*

Resistance exercise: 2-3 days per week for ~45 minutes per session at moderate to vigorous intensity.

Neuromotor exercise: 2 days per week for 20-30 minutes per session at moderate intensity.

Flexibility exercise: ≥2-3 days per week for ~5 minutes per session*, stretching to the point of tightness or slight discomfort. **Can be implemented into warm-ups or cool-downs.*

Aerobic Exercise Workout Examples at Moderate Intensity Week 11.

Component	Time	Type	Examples
1) Warm-up	5 minutes	Aerobic activity or dynamic stretching	- Walking at a talking pace on a treadmill<br>- Walking to the gym<br>- Dynamic stretching (3-6 sets of 30-90 sec with 15 sec rest periods between sets):<br>• Marching in place<br>• Walking lunges<br>• Hip circles or openers<br>• Arm swings/arm circles<br>• Torso twists
2) Aerobic Exercise	30-60 minutes<br><br>OR<br><br>15-30 minutes	Steady state moderate intensity aerobic activity<br><br>OR<br><br>Steady state vigorous intensity aerobic activity or high intensity interval training	- Walking at a pace that increases your breathing rate noticeably<br>- Peddling slowly on a stationary bicycle or leisurely cycling<br>- Peddling slowly on an elliptical machine<br>- Rowing slowly on a machine<br>- Slow dancing<br>- Aqua-aerobics<br><br>- Walking very briskly, jogging, or running<br>- Peddling fast on a stationary bicycle or cycling fast or uphill<br>- Peddling fast on an elliptical machine<br>- Rowing fast on a machine<br>- Stepping on the stair stepper machine<br>- Fast dancing<br>- Aerobics
3) Cool-down	5 minutes	Static flexibility OR slowly reduce the pace of aerobic exercise	- Static stretching (10-30 seconds per stretch, 2-4 repetitions of each exercise):<br>• Standing calf stretch<br>• Quadricep stretch<br>• Kneeling hip flexor stretch (iliopsoas)<br>• Seated hamstring stretch<br>• Child's pose stretch

			• Chest stretch<br>• Cross-body shoulder stretch<br>• Neck lateral flexion stretch

Resistance Exercise Workout Examples at Moderate to Vigorous Intensity Week 11.

Component	Time	Type	Example exercises with machines, free weights, bodyweight, or resistance bands
Resistance Exercise (Day 1)	~45 minutes	Resistance training for all major muscle groups	3-4 sets of 8-12 repetitions (rest 1.5-2.5 minutes between sets)<br>RPE 12-13 to 14-17; 1-RM: 50-85%<br>• Chest press<br>• Seated row<br>• Leg press<br>• Hip bridges<br>• Leg curls<br>• Arm curls<br>• Abdominal flexions<br>• Planks
Resistance Exercise (Day 2)	~45 minutes	Resistance training for all major muscle groups	3-4 sets of 8-12 repetitions (rest 1.5-2.5 minutes between sets)<br>RPE 12-13 to 14-17; 1-RM: 50-85%<br>• Shoulder press<br>• Lat pull-downs<br>• Hip kickbacks<br>• Hip abductions<br>• Leg extensions<br>• Triceps extensions<br>• Abdominal twists<br>• Side planks
Resistance Exercise (Day 3)	~45 minutes	Resistance training for all major muscle groups	3-4 sets of 8-12 repetitions (rest 1.5-2.5 minutes between sets)<br>RPE 12-13 to 14-17; 1-RM: 50-85%)<br>• Pick 6-10 exercises from above to train major muscle groups

Neuromotor Exercise Workout Examples at Moderate Intensity Week 11.

Component	Time	Type	Examples
Neuromotor Exercise	20-30 minutes	Training that involves motor skills such as balance, coordination, agility	- Yoga (power/Surya Namaskar)<br>- Tai chi (qi gong standing/yang style)<br>- Pilates (POUND® with drumming)<br>- Single leg or Bosu ball exercises<br>- Balance exercises (15-30 seconds per hold, 5-6 cycles):<br>• tandem stance<br>• one-legged stance<br>• stepping over obstacles<br>• heel or toe walks<br>• walking while turning the head

Week 12

Aerobic exercise: ≥5 days per week for 30-60 minutes* per day at moderate intensity or 15-30 minutes* per day at vigorous intensity (or a combination), to total 250-300 minutes per week. **Can be accumulated in 10+ minute bouts or sessions over the day.*

Resistance exercise: 2-3 days per week for ~45 minutes per session at moderate to vigorous intensity.

Neuromotor exercise: 2 days per week for 20-30 minutes per session at moderate intensity.

Flexibility exercise: ≥2-3 days per week for ~5 minutes per session*, stretching to the point of tightness or slight discomfort. **Can be implemented into warm-ups or cool-downs.*

Aerobic Exercise Workout Examples at Moderate Intensity Week 12.

Component	Time	Type	Examples
1) Warm-up	5 minutes	Aerobic activity or dynamic stretching	- Walking at a talking pace on a treadmill<br>- Walking to the gym<br>- Dynamic stretching (3-6 sets of 30-90 sec with 15 sec rest periods between sets):<br>• Marching in place<br>• Walking lunges<br>• Hip circles or openers<br>• Arm swings/arm circles<br>• Torso twists
2) Aerobic Exercise	30-60 minutes<br><br>OR<br><br>15-30 minutes	Steady state moderate intensity aerobic activity<br><br>OR<br><br>Steady state vigorous intensity aerobic activity or high intensity interval training	- Walking at a pace that increases your breathing rate noticeably<br>- Peddling slowly on a stationary bicycle or leisurely cycling<br>- Peddling slowly on an elliptical machine<br>- Rowing slowly on a machine<br>- Slow dancing<br>- Aqua-aerobics<br><br>- Walking very briskly, jogging, or running<br>- Peddling fast on a stationary bicycle or cycling fast or uphill<br>- Peddling fast on an elliptical machine<br>- Rowing fast on a machine<br>- Stepping on the stair stepper machine<br>- Fast dancing<br>- Aerobics
3) Cool-down	5 minutes	Static flexibility OR slowly reduce the pace of aerobic exercise	- Static stretching (10-30 seconds per stretch, 2-4 repetitions of each exercise):<br>• Standing calf stretch<br>• Quadricep stretch<br>• Kneeling hip flexor stretch (iliopsoas)<br>• Seated hamstring stretch<br>• Child's pose stretch

			• Chest stretch<br>• Cross-body shoulder stretch<br>• Neck lateral flexion stretch

Resistance Exercise Workout Examples at Moderate to Vigorous Intensity Week 12.

Component	Time	Type	Example exercises with machines, free weights, bodyweight, or resistance bands
Resistance Exercise (Day 1)	~45 minutes	Resistance training for all major muscle groups	3-4 sets of 8-12 repetitions (rest 1.5-2.5 minutes between sets)<br>RPE 12-13 to 14-17; 1-RM: 50-85%<br>• Chest press<br>• Seated row<br>• Leg press<br>• Hip bridges<br>• Leg curls<br>• Arm curls<br>• Abdominal flexions<br>• Planks
Resistance Exercise (Day 2)	~45 minutes	Resistance training for all major muscle groups	3-4 sets of 8-12 repetitions (rest 1.5-2.5 minutes between sets)<br>RPE 12-13 to 14-17; 1-RM: 50-85%<br>• Shoulder press<br>• Lat pull-downs<br>• Hip kickbacks<br>• Hip abductions<br>• Leg extensions<br>• Triceps extensions<br>• Abdominal twists<br>• Side planks
Resistance Exercise (Day 3)	~45 minutes	Resistance training for all major muscle groups	3-4 sets of 8-12 repetitions (rest 1.5-2.5 minutes between sets)<br>RPE 12-13 to 14-17; 1-RM: 50-85%)<br>• Pick 6-10 exercises from above to train major muscle groups

Neuromotor Exercise Workout Examples at Moderate Intensity Week 12.

Component	Time	Type	Examples
Neuromotor Exercise	20-30 minutes	Training that involves motor skills such as balance, coordination, agility	- Yoga (power/Surya Namaskar)<br>- Tai chi (qi gong standing/yang style)<br>- Pilates (POUND® with drumming)<br>- Single leg or Bosu ball exercises<br>- Balance exercises (15-30 seconds per hold, 5-6 cycles):<br>• tandem stance<br>• one-legged stance<br>• stepping over obstacles<br>• heel or toe walks<br>• walking while turning the head

Participant 12-Week Exercise Program Information Packet

Participant ID:

Date Provided:

12-Week Start Date:

12-Week End Date:

Principle Investigators:

Dr. Antonio Fernandez, MD, FACC, FAHA
Medical Director of Preventive Cardiology, Hartford Hospital

Dr. Linda Pescatello, PhD, FACSM
Board of Trustees Distinguished Professor of Kinesiology, University of Connecticut

Dr. Peter Robinson, MD
Assistant Professor of Cardiology, UConn Health

UConn Graduate Research Assistant:

Alexander Wright, MS
Email: Alexander.Wright@hhchealth.org
Phone: (860) 486-6814

This research is approved by the Hartford HealthCare Institutional Review Board, with reliance agreement at UConn Storrs and UConn Health.

Contents Page

Exercise Program for Diabetes 3
Special Considerations 4
Exercise Type Definitions 5
Exercise Type Examples 6
Exercise Intensity Definitions 8
How to Take Your Pulse 9
Self-Monitor Your Physical Activity 9
Guidelines for Exercise Progression 10
Contact Details for the UConn Graduate Research Assistant 10
12 Week Exercise Program Progression Guidance 11
Week 1 12
Week 2 14
Week 3 16
Week 4 18
Week 5 20
Week 6 22
Week 7 24
Week 8 26
Week 9 28
Week 10 30
Week 11 32
Week 12 34

Exercise Program for Diabetes

FITT	Aerobic	Resistance	Neuromotor*	Flexibility
Frequency	3-7 days per week<br>No more than 2 consecutive days without activity	A minimum of 2 nonconsecutive days per week, but preferably 3 days	≥2-3 days per week	≥2-3 days per week
Intensity	**Moderate intensity:** you can talk comfortably but not sing<br>64-76% HRmax; 40%-59% of VO_2R or HRR; RPE 12-13<br>to **vigorous intensity:** you cannot say more than 5 words without grasping for breath<br>77-95% HRmax; 60%-89% of VO_2R or HRR; RPE 14-17	**Moderate intensity:** 50%-69% of 1-RM for 12-18 repetitions<br>to **vigorous intensity:** 70%-85% of 1-RM for 8-12 repetitions, to improve strength.	Light to moderate intensity	Stretch to the point of tightness or slight discomfort
Time	T1DM and T2DM<br>150-300 min per week at moderate or 75-150 min per week at vigorous intensity, or a combination thereof	At least 8-10 exercises with 1-3 sets of 10-15 repetitions to near fatigue per set early in training	Balance for any duration.	Hold static stretch for 10-30 sec; 2-4 repetitions of each exercise.
Type	Prolonged, rhythmic activities using large muscle groups (e.g., walking, cycling, swimming)<br>Continuous activity or HIIT	Resistance machines, free weights, resistance bands, and/or body weight	Exercise involving motor skills and/or functional body weight and flexibility exercise such as yoga, Pilates, and tai chi.	Static, dynamic, other stretching, yoga

FITT = Frequency, Intensity, Time and Type; 1-RM = 1 Repetition Maximum; RPE = Rating of Perceived Exertion (Borg 6-20 scale); HRmax = Heart Rate Maximum; HRR = Heart Rate Reserve; T1DM = Type 2 Diabetes; T2DM = Type 2 Diabetes; VO_2R = Oxygen Uptake Reserve; PNF = Proprioceptive Neuromuscular Facilitation.

*Neuromotor functional body weight exercise can be substituted for resistance exercise, and depending on the amount of flexibility exercise integrated into a session, neuromotor flexibility exercise can be substituted for flexibility exercise depending on patient/client preference.

Reference: American College of Sports Medicine, Ozemek C, Bonikowske AR, Christle JW, Gallo PM, eds. ACSM’s Guidelines for Exercise Testing and Prescription. 12th Ed, p.353. Wolters Kluwer; 2026.

Special Considerations

Type of diabetes: Type1/Type2

Medication Considerations: Yes/No

Diabetes Related Health Conditions (e.g., autonomic neuropathy): Yes/No

If YES, will list medication(s) taken and dose:

If YES, will list diabetes related health conditions:

If YES, will list potential side effects of medication or diabetes related health conditions as related to exercise.

If YES, will list special considerations for medication or diabetes related health conditions as related to exercise.

You should wait to exercise if your blood sugar is below 70 mg/dL (3.9 mmol/L). Your blood sugar should be 90 to 250 mg/dL (5.0 to 14.9 mmol/L) before exercise.

Your blood sugar may drop quickly during exercise. You may experience symptoms of low blood sugar even when your level is above 70 mg/dL (3.9 mmol/L). On the other hand, you may not experience symptoms of low blood sugar even when your level is below 70 mg/dL (3.9 mmol/L).

You should be aware of symptoms of low blood sugar. Common symptoms may include shakiness, weakness, abnormal sweating, nervousness, anxiety, tingling of the mouth and fingers, and hunger. More serious symptoms may include headache, vision disturbances, trouble thinking clearly, confusion, memory loss, seizures and coma.

During exercise, you may consider carrying a medical ID that shows you have diabetes, a cell phone, and glucose tablets or a fast-acting sugar snack.

Your blood sugar may drop immediately after exercise. Your blood sugar can stay lower for up to 12 hours after performing aerobic exercise at moderate intensity such as walking.

Exercise Type Definitions

Aerobic Exercise: Continuous exercise involving large muscle groups such as walking, running, riding a bicycle, cardio machines and rowing.

> **Steady State Aerobic Exercise:** Maintaining exercise at light to moderate intensity.

> **High Intensity Interval Training:** Performing bouts of higher intensity exercise separated by rest.

Resistance Exercise: Exercises that use opposing forces to strengthen or develop muscles such as weight training with free weights, resistance machines, or using resistance bands or body weight.

Neuromotor Exercise: Exercises that combine balance, coordination, and agility such as yoga, tai chi, and Pilates.

Flexibility: Movements that improve the range of motion of a joint.

> **Dynamic Flexibility:** Slow moving stretches that increase reach and range of motion as the movement is repeated. Examples: Leg swings, arm swings, torso twists.

> **Static Flexibility:** Slowly stretching a muscle/tendon group and holding the position for 10-30 sec. Examples: Pike stretch, glute stretch, quad stretch

> **Proprioceptive Neuromuscular Facilitation (PNF):** Applying force with a muscle against an opposing force followed by performing a static stretch of the muscle.

Concurrent Exercise: Performing aerobic and resistance exercise in the same session or near one another.

Major Muscle Groups: Shoulders, chest, back, hamstrings, quadriceps, calves, biceps, triceps, core.

Exercise Type Examples

Aerobic Exercise

Light Intensity (RPE 8-11)	Moderate Intensity (RPE 12-13)	Vigorous Intensity (RPE 14-20)
Walking slowly, leisurely	Walking the dog or walking outside	Brisk walking or jogging
Cycling slowly with a pedal desk	Cycling at a self-selected comfortable pace	Cycling at a moderate pace
Group class - Vinyasa yoga, tai chi (qi gong), or stretching	Group class – Power yoga or tai chi (yang style)	Group class - Zumba or fast ballroom dancing
Cleaning, sweeping, or washing dishes slowly	Water aerobics	Swimming laps, freestyle
Pickleball and Tennis, serving practice	Pickleball and Tennis, doubles	Pickleball and Tennis, singles

Resistance Exercise

Light Intensity (RPE 8-11)	Moderate Intensity (RPE 12-13)	Vigorous Intensity (RPE 14-20)
Yard work at a slow pace	Gardening - watering, weeding, planting	Gardening - using heavy tools, digging or filling garden
Canoeing at a slow pace	Kayaking at a self-selected comfortable pace	Rowing on a stationary ergometer
Group class - Vinyasa yoga, tai chi (qi gong)	Group class – Power yoga or tai chi (yang style)	Group class - circuit training with minimal rest
Bodyweight exercises (curl-ups, planks)	Bodyweight exercises (push-ups, lunges)	Bodyweight exercises (jumping jacks, burpees)
Lifting weights <50% of one repetition maximum (being able to perform 15-20 reps at this weight)	Lifting weights 50-69% of one repetition maximum (being able to perform 12-18 reps at this weight)	Lifting weights 70 to 85% of one repetition maximum (being able to perform 8-12 reps at this weight)

Neuromotor Exercise

**Light to Moderate Intensity (RPE 8-13)**	**Moderate Intensity (RPE 12-13)**
Yoga (Hatha sitting/Vinyasa/ Nadisodhana)	Yoga (power/Surya Namaskar)
Tai chi (qi gong, sitting)	Tai chi (qi gong standing/yang style)
Pilates (traditional)	Pilates (POUND® with drumming)
Balance exercises (beginner)	Balance exercises (general)
Slow dancing	Functional bodyweight exercises

Exercise Intensity Definitions

Adapted Version of Borg 6-20 Rating of Perceived Exertion (RPE) Scale with Exercise Intensity Differentiation:

Borg 6-20 RPE	Intensity	Intensity Definition
6	No exertion	
7	Very Light	
8		
9	Light	A level of physical exertion that causes slight increases in heart rate and breathing (i.e., warm up with dynamic flexibility, cool down with slow aerobic movements and static stretching). ***Use the talk test:*** *Light intensity should make your breathing slightly increase, but you can still talk and sing easily.*<br>Aerobic exercise: <64% HRmax, <40% VO_2R or HRR
10		
11		
12	Moderate	A level of physical exertion that causes increases in heart rate and breathing (i.e., brisk walking, weight training at an intensity that is not hard, but takes effort). ***Use the talk test:*** *Moderate intensity should make your breathing rate increase noticeably. You can still talk but not sing.*<br>Aerobic exercise: 64-76% HRmax, 40-59% VO_2R or HRR.<br>Resistance exercise: 50-69% of 1-RM intensity of 12-18 reps.
13		
14	Vigorous	A level of physical exertion that causes substantial increases in heart rate and breathing (i.e., running, weight training until fatigue). ***Use the talk test:*** *Vigorous intensity should make you breathe hard enough that you can only say a few words before you have to take a breath and you can't sing.*<br>Aerobic exercise: 77-95% HRmax, ≥ 60% VO_2R or HRR<br>Resistance exercise: 70-85% of 1-RM intensity of 8-12 reps.
15		
16		
17		
18	Near Maximal to Maximal	
19		
20		

HRmax = Heart Rate Maximum. The HRmax is the maximum heart rate you can achieve during exercise. HRmax declines with age.

HRR = Heart Rate Reserve. The percentage of HRR is calculated as: (the heart rate during exercise – the heart rate at rest) ÷ (the maximum heart rate during exercise – the heart rate at rest) × 100%.

VO_2R = Oxygen Uptake Reserve. The percentage of VO_2R is calculated as: (the rate of oxygen consumption during exercise – the rate of oxygen consumption at rest) ÷ (the maximum rate of oxygen consumption during exercise – the rate of oxygen consumption at rest) × 100%.

1-RM = 1 Repetition Maximum. The 1-RM is the maximum weight lifted for a single repetition for a given exercise.

How to Take Your Pulse

1) You can use a technique called pulse palpitation, which involves “feeling” the pulse.
2) Place your index finger and middle fingers over the radial artery, located near the thump side of the wrist.
3) Count the pulse for 30-60 seconds. The 30-second count is multiplied by 2 to determine the 1-minute resting heart rate in beats per minute (bpm).

Self-Monitor Your Physical Activity

1) The *Timeline Followback for Exercise* is a self-report tool for exercise and will be completed weekly.

2) Please fill out each day of the week in terms of the exercise you perform to the best of your abilities as described below:

   - **Did you exercise?** At any point in the day, did you exercise? This is answered as “yes” or “no”. This includes both planned exercise and any other physical activity that is completed that day.
   - **Type(s):** For each type of exercise bout you completed that day, record the type(s) of the exercise you performed (i.e., walking, swimming, weightlifting etc.)
   - **Time (minutes):** For each type of exercise bout you completed that day, record how long it took you to complete the bout in minutes. Time for each exercise should be listed in the same order that it was listed for type(s) of exercise.
   - **Borg Rating of Perceived Exertion (RPE) on a scale of 6 to 20:** Record the Borg RPE for each exercise bout you completed that day using the scale and instructions below. RPE for each exercise should be listed in the same order that it was listed for type(s) of exercise.

   Please refer to the *Timeline Followback for Exercise* for further instructions.

3) For each week of the exercise program, transfer your recordings to an electronic diary in REDCap by the first day of each week (Sundays by 11:59 PM).

Guidelines for Exercise Progression

Aerobic training:

"Start low and go slow"

1) Start at light-to-moderate intensity exercise
2) Increase exercise duration (time) per day by 5-10 min every 1-2 weeks.
3) Increase the number of days per week gradually over 12 weeks.
4) Increase exercise intensity when you perceive reductions in your exertion during exercise sessions and gradually transition to vigorous intensity exercise.

Resistance training:

1) When you can perform 2 more repetitions than what was prescribed during two consecutive sessions for a given exercise, increase the load by 2.5%-5%, all while maintaining proper form/technique.
2) Increase the number of days per week the muscle groups are trained over 12 weeks.
3) Increase the number of sets per muscle group per session gradually as tolerated.

Neuromotor training:

1) Increase exercise intensity by performing more challenging or advanced balances, postures, or movements over 12 weeks.

Flexibility training:

1) Increase the number of days per week of stretching over 12 weeks.

Contact Details for the UConn Graduate Research Assistant

Alexander Wright

Email: Alexander.Wright@hhchealth.org

Phone: (860) 486-6814

12 Week Exercise Program Progression Guidance

<table>
<tr><th></th><th>Aerobic</th><th>Resistance</th><th>Neuromotor</th><th>Flexibility</th></tr>
<tr><td>Week 1</td><td rowspan="2">3-4 days per week at light to moderate intensity for ~30 minutes</td><td rowspan="3">1-2 days per week at moderate intensity for ~30 minutes</td><td rowspan="4">1 day per week at light to moderate intensity for 20-30 minutes</td><td rowspan="6">2 days per week for 5 minutes</td></tr>
<tr><td>Week 2</td></tr>
<tr><td>Week 3</td><td rowspan="2">3-4 days per week at light to moderate intensity for 30-40 minutes</td></tr>
<tr><td>Week 4</td><td rowspan="3">2 days per week at moderate intensity for ~30 minutes</td></tr>
<tr><td>Week 5</td><td rowspan="2">3-4 days per week at moderate intensity for 30-50 minutes</td><td rowspan="8">≥2-3 days per week at moderate intensity for 20-30 minutes</td></tr>
<tr><td>Week 6</td></tr>
<tr><td>Week 7</td><td rowspan="3">3-5 days per week at moderate intensity for 30-50 minutes and/or vigorous intensity for 15-25 minutes</td><td rowspan="6">2-3 days* per week at moderate to vigorous intensity for ~45 minutes
*3 days preferred</td><td rowspan="6">2-3 days per week for 5 minutes</td></tr>
<tr><td>Week 8</td></tr>
<tr><td>Week 9</td></tr>
<tr><td>Week 10</td><td rowspan="3">3-7 days per week at moderate intensity for 30-60 minutes and/or vigorous intensity for 15-30 minutes</td></tr>
<tr><td>Week 11</td></tr>
<tr><td>Week 12</td></tr>
</table>

Week 1

Aerobic exercise: 3-4 days* per week for ~30 minutes** per day at light to moderate intensity, to total 90 minutes per week. **No more than 2 consecutive days without exercise. **Can be small doses accumulated throughout the day.*

Resistance exercise: 1-2 days* per week for ~30 minutes per session at moderate intensity. **Perform on nonconsecutive days.*

Neuromotor exercise: 1 day per week for 20-30 minutes per session at light to moderate intensity.

Flexibility exercise: 2 days per week for ~5 minutes per session*, stretching to the point of tightness or slight discomfort. **Can be included in warm-ups or cool-downs.*

Aerobic Exercise Workout Examples at Light to Moderate Intensity Week 1.

Component	Time	Type	Examples
1) Warm-up	5 minutes	Aerobic activity or dynamic stretching	- Walking at a talking pace on a treadmill<br>- Walking to the gym<br>- Dynamic stretching (3-6 sets of 30-90 sec with 15 sec rest periods between sets):<br>• Marching in place<br>• Walking lunges<br>• Hip circles or openers<br>• Arm swings/arm circles<br>• Torso twists
2) Aerobic Exercise	~30 minutes	Steady state moderate intensity aerobic activity	- Walking at a pace that increases your breathing rate noticeably<br>- Peddling slowly on a stationary bicycle or leisurely cycling<br>- Peddling slowly on an elliptical machine<br>- Rowing slowly on a machine<br>- Slow dancing<br>- Aqua-aerobics
3) Cool-down	5 minutes	Static flexibility OR slowly reduce the pace of aerobic exercise	- Static stretching (10-30 seconds per stretch, 2-4 repetitions of each exercise):<br>• Standing calf stretch<br>• Quadricep stretch<br>• Kneeling hip flexor stretch (iliopsoas)<br>• Seated hamstring stretch<br>• Child’s pose stretch<br>• Chest stretch<br>• Cross-body shoulder stretch<br>• Neck lateral flexion stretch

Resistance Exercise Workout Examples at Moderate Intensity Week 1.

Component	Time	Type	Example exercises with machines, free weights, bodyweight, or resistance bands
Resistance Exercise (Day 1)	~30 minutes	Resistance training for all major muscle groups	1-2 sets of 10-15 repetitions (rest 1.5-2 minutes between sets)<br>RPE 12-13; 1-RM: 50-69%<br>• Chest press<br>• Seated row<br>• Leg press<br>• Hip bridges<br>• Leg curls<br>• Arm curls<br>• Abdominal flexions<br>• Planks
Resistance Exercise (Day 2)	~30 minutes	Resistance training for all major muscle groups	1-2 sets of 10-15 repetitions (rest 1.5-2 minutes between sets):<br>RPE 12-13; 1-RM: 50-69%<br>• Shoulder press<br>• Lat pull-downs<br>• Hip kickbacks<br>• Hip abductions<br>• Leg extensions<br>• Triceps extensions<br>• Abdominal twists<br>• Side planks

Neuromotor Exercise Workout Examples at Light to Moderate Intensity Week 1.

Component	Time	Type	Examples
Neuromotor Exercise	20-30 minutes	Training that involves motor skills such as balance, coordination, agility	- Yoga (Hatha sitting/Vinyasa/ Nadisodhana)<br>- Tai chi (qi gong, sitting)<br>- Pilates (traditional)<br>- Balance exercise (15-30 seconds per hold, 5-6 cycles):<br>• two-legged stance<br>• semi-tandem stance<br>• reaching from a narrow stance<br>• tandem walking<br>• standing with eyes closed

Week 2

Aerobic exercise: 3-4 days* per week for ~30 minutes** per day at light to moderate intensity, to total 90 minutes per week. **No more than 2 consecutive days without exercise. **Can be small doses accumulated throughout the day.*

Resistance exercise: 1-2 days* per week for ~30 minutes per session at moderate intensity. **Perform on nonconsecutive days.*

Neuromotor exercise: 1 day per week for 20-30 minutes per session at light to moderate intensity.

Flexibility exercise: 2 days per week for ~5 minutes per session*, stretching to the point of tightness or slight discomfort. **Can be implemented into warm-ups or cool-downs.*

Aerobic Exercise Workout Examples at Light to Moderate Intensity Week 2.

Component	Time	Type	Examples
1) Warm-up	5 minutes	Aerobic activity or dynamic stretching	- Walking at a talking pace on a treadmill<br>- Walking to the gym<br>- Dynamic stretching (3-6 sets of 30-90 sec with 15 sec rest periods between sets):<br>• Marching in place<br>• Walking lunges<br>• Hip circles or openers<br>• Arm swings/arm circles<br>• Torso twists
2) Aerobic Exercise	~30 minutes	Steady state moderate intensity aerobic activity	- Walking at a pace that increases your breathing rate noticeably<br>- Peddling slowly on a stationary bicycle or leisurely cycling<br>- Peddling slowly on an elliptical machine<br>- Rowing slowly on a machine<br>- Slow dancing<br>- Aqua-aerobics
3) Cool-down	5 minutes	Static flexibility OR slowly reduce the pace of aerobic exercise	- Static stretching (10-30 seconds per stretch, 2-4 repetitions of each exercise):<br>• Standing calf stretch<br>• Quadricep stretch<br>• Kneeling hip flexor stretch (iliopsoas)<br>• Seated hamstring stretch<br>• Child's pose stretch<br>• Chest stretch<br>• Cross-body shoulder stretch<br>• Neck lateral flexion stretch

Resistance Exercise Workout Examples at Moderate Intensity Week 2.

Component	Time	Type	Example exercises with machines, free weights, bodyweight, or resistance bands
Resistance Exercise (Day 1)	~30 minutes	Resistance training for all major muscle groups	1-2 sets of 10-15 repetitions (rest 1.5-2 minutes between sets)<br>RPE 12-13; 1-RM: 50-69%<br>• Chest press<br>• Seated row<br>• Leg press<br>• Hip bridges<br>• Leg curls<br>• Arm curls<br>• Abdominal flexions<br>• Planks
Resistance Exercise (Day 2)	~30 minutes	Resistance training for all major muscle groups	1-2 sets of 10-15 repetitions (rest 1.5-2 minutes between sets)<br>RPE 12-13; 1-RM: 50-69%<br>• Shoulder press<br>• Lat pull-downs<br>• Hip kickbacks<br>• Hip abductions<br>• Leg extensions<br>• Triceps extensions<br>• Abdominal twists<br>• Side planks

Neuromotor Exercise Workout Examples at Light to Moderate Intensity Week 2.

Component	Time	Type	Examples
Neuromotor Exercise	20-30 minutes	Training that involves motor skills such as balance, coordination, agility	- Yoga (Hatha sitting/Vinyasa/ Nadisodhana)<br>- Tai chi (qi gong, sitting)<br>- Pilates (traditional)<br>- Balance exercise (15-30 seconds per hold, 5-6 cycles):<br>• two-legged stance<br>• semi-tandem stance<br>• reaching from a narrow stance<br>• tandem walking<br>• standing with eyes closed

Week 3

Aerobic exercise: 3-4 days* per week for 30-40 minutes** per day at light to moderate intensity, to total 125 minutes per week. **No more than 2 consecutive days without exercise. **Can be small doses accumulated throughout the day.*

Resistance exercise: 1-2 days* per week for ~30 minutes per session at moderate intensity. **Perform on nonconsecutive days.*

Neuromotor exercise: 1 day per week for 20-30 minutes per session at light to moderate intensity.

Flexibility exercise: 2 days per week for ~5 minutes per session*, stretching to the point of tightness or slight discomfort. **Can be implemented into warm-ups or cool-downs.*

Aerobic Exercise Workout Examples at Light to Moderate Intensity Week 3.

Component	Time	Type	Examples
1) Warm-up	5 minutes	Aerobic activity or dynamic stretching	- Walking at a talking pace on a treadmill<br>- Walking to the gym<br>- Dynamic stretching (3-6 sets of 30-90 sec with 15 sec rest periods between sets):<br>• Marching in place<br>• Walking lunges<br>• Hip circles or openers<br>• Arm swings/arm circles<br>• Torso twists
2) Aerobic Exercise	30-40 minutes	Steady state moderate intensity aerobic activity	- Walking at a pace that increases your breathing rate noticeably<br>- Peddling slowly on a stationary bicycle or leisurely cycling<br>- Peddling slowly on an elliptical machine<br>- Rowing slowly on a machine<br>- Slow dancing<br>- Aqua-aerobics
3) Cool-down	5 minutes	Static flexibility OR slowly reduce the pace of aerobic exercise	- Static stretching (10-30 seconds per stretch, 2-4 repetitions of each exercise):<br>• Standing calf stretch<br>• Quadricep stretch<br>• Kneeling hip flexor stretch (iliopsoas)<br>• Seated hamstring stretch<br>• Child's pose stretch<br>• Chest stretch<br>• Cross-body shoulder stretch<br>• Neck lateral flexion stretch

Resistance Exercise Workout Examples at Moderate Intensity Week 3.

Component	Time	Type	Example exercises with machines, free weights, bodyweight, or resistance bands
Resistance Exercise (Day 1)	~30 minutes	Resistance training for all major muscle groups	1-2 sets of 10-15 repetitions (rest 1.5-2 minutes between sets)<br>RPE 12-13; 1-RM: 50-69%<br>• Chest press<br>• Seated row<br>• Leg press<br>• Hip bridges<br>• Leg curls<br>• Arm curls<br>• Abdominal flexions<br>• Planks
Resistance Exercise (Day 2)	~30 minutes	Resistance training for all major muscle groups	1-2 sets of 10-15 repetitions (rest 1.5-2 minutes between sets)<br>RPE 12-13; 1-RM: 50-69%<br>• Shoulder press<br>• Lat pull-downs<br>• Hip kickbacks<br>• Hip abductions<br>• Leg extensions<br>• Triceps extensions<br>• Abdominal twists<br>• Side planks

Neuromotor Exercise Workout Examples at Light to Moderate Intensity Week 3.

Component	Time	Type	Examples
Neuromotor Exercise	20-30 minutes	Training that involves motor skills such as balance, coordination, agility	- Yoga (Hatha sitting/Vinyasa/ Nadisodhana)<br>- Tai chi (qi gong, sitting)<br>- Pilates (traditional)<br>- Balance exercise (15-30 seconds per hold, 5-6 cycles):<br>• two-legged stance<br>• semi-tandem stance<br>• reaching from a narrow stance<br>• tandem walking<br>• standing with eyes closed

Week 4

Aerobic exercise: 3-4 days* per week for 30-40 minutes* per day at moderate intensity, to total 125 minutes per week. **No more than 2 consecutive days without exercise. **Can be small doses accumulated throughout the day.*

Resistance exercise: 1-2 days* per week for ~30 minutes per session at moderate intensity. ***Perform on nonconsecutive days.*

Neuromotor exercise: 1 day per week for 20-30 minutes per session at light to moderate intensity.

Flexibility exercise: 2 days per week for ~5 minutes per session*, stretching to the point of tightness or slight discomfort. **Can be implemented into warm-ups or cool-downs.*

Aerobic Exercise Workout Examples at Moderate Intensity Week 4.

Component	Time	Type	Examples
1) Warm-up	5 minutes	Aerobic activity or dynamic stretching	- Walking at a talking pace on a treadmill<br>- Walking to the gym<br>- Dynamic stretching (3-6 sets of 30-90 sec with 15 sec rest periods between sets):<br>• Marching in place<br>• Walking lunges<br>• Hip circles or openers<br>• Arm swings/arm circles<br>• Torso twists
2) Aerobic Exercise	30-40 minutes	Steady state moderate intensity aerobic activity	- Walking at a pace that increases your breathing rate noticeably<br>- Peddling slowly on a stationary bicycle or leisurely cycling<br>- Peddling slowly on an elliptical machine<br>- Rowing slowly on a machine<br>- Slow dancing<br>- Aqua-aerobics
3) Cool-down	5 minutes	Static flexibility OR slowly reduce the pace of aerobic exercise	- Static stretching (10-30 seconds per stretch, 2-4 repetitions of each exercise):<br>• Standing calf stretch<br>• Quadricep stretch<br>• Kneeling hip flexor stretch (iliopsoas)<br>• Seated hamstring stretch<br>• Child’s pose stretch<br>• Chest stretch<br>• Cross-body shoulder stretch<br>• Neck lateral flexion stretch

Resistance Exercise Workout Examples at Moderate Intensity Week 4.

Component	Time	Type	Example exercises with machines, free weights, bodyweight, or resistance bands
Resistance Exercise (Day 1)	~30 minutes	Resistance training for all major muscle groups	2-3 sets of 8-12 repetitions (rest 1.5-2 minutes between sets)<br>RPE 12-13; 1-RM: 50-69%<br>• Chest press<br>• Seated row<br>• Leg press<br>• Hip bridges<br>• Leg curls<br>• Arm curls<br>• Abdominal flexions<br>• Planks
Resistance Exercise (Day 2)	~30 minutes	Resistance training for all major muscle groups	2-3 sets of 8-12 repetitions (rest 1.5-2 minutes between sets)<br>RPE 12-13; 1-RM: 50-69%<br>• Shoulder press<br>• Lat pull-downs<br>• Hip kickbacks<br>• Hip abductions<br>• Leg extensions<br>• Triceps extensions<br>• Abdominal twists<br>• Side planks

Neuromotor Exercise Workout Examples at Light to Moderate Intensity Week 4.

Component	Time	Type	Examples
Neuromotor Exercise	20-30 minutes	Training that involves motor skills such as balance, coordination, agility	- Yoga (Hatha sitting/Vinyasa/ Nadisodhana)<br>- Tai chi (qi gong, sitting)<br>- Pilates (traditional)<br>- Balance exercise (15-30 seconds per hold, 5-6 cycles):<br>• two-legged stance<br>• semi-tandem stance<br>• reaching from a narrow stance<br>• tandem walking<br>• standing with eyes closed

Week 5

Aerobic exercise: 3-4 days* per week for 30-50 minutes* per day at moderate intensity, to total 150 minutes per week. **No more than 2 consecutive days without exercise. **Can be small doses accumulated throughout the day.*

Resistance exercise: 1-2 days* per week for ~30 minutes per session at moderate intensity. **Perform on nonconsecutive days.*

Neuromotor exercise: 2 days per week for 20-30 minutes per session at moderate intensity.

Flexibility exercise: 2 days per week for ~5 minutes per session*, stretching to the point of tightness or slight discomfort. **Can be implemented into warm-ups or cool-downs.*

Aerobic Exercise Workout Examples at Moderate Intensity Week 5.

Component	Time	Type	Examples
1) Warm-up	5 minutes	Aerobic activity or dynamic stretching	- Walking at a talking pace on a treadmill<br>- Walking to the gym<br>- Dynamic stretching (3-6 sets of 30-90 sec with 15 sec rest periods between sets):<br>• Marching in place<br>• Walking lunges<br>• Hip circles or openers<br>• Arm swings/arm circles<br>• Torso twists
2) Aerobic Exercise	30-50 minutes	Steady state moderate intensity aerobic activity	- Walking at a pace that increases your breathing rate noticeably<br>- Peddling slowly on a stationary bicycle or leisurely cycling<br>- Peddling slowly on an elliptical machine<br>- Rowing slowly on a machine<br>- Slow dancing<br>- Aqua-aerobics
3) Cool-down	5 minutes	Static flexibility OR slowly reduce the pace of aerobic exercise	- Static stretching (10-30 seconds per stretch, 2-4 repetitions of each exercise):<br>• Standing calf stretch<br>• Quadricep stretch<br>• Kneeling hip flexor stretch (iliopsoas)<br>• Seated hamstring stretch<br>• Child’s pose stretch<br>• Chest stretch<br>• Cross-body shoulder stretch<br>• Neck lateral flexion stretch

Resistance Exercise Workout Examples at Moderate Intensity Week 5.

Component	Time	Type	Example exercises with machines, free weights, bodyweight, or resistance bands
Resistance Exercise (Day 1)	~30 minutes	Resistance training for all major muscle groups	2-3 sets of 8-12 repetitions (rest 1.5-2 minutes between sets)<br>RPE 12-13; 1-RM: 50-69%<br>• Chest press<br>• Seated row<br>• Leg press<br>• Hip bridges<br>• Leg curls<br>• Arm curls<br>• Abdominal flexions<br>• Planks
Resistance Exercise (Day 2)	~30 minutes	Resistance training for all major muscle groups	2-3 sets of 8-12 repetitions (rest 1.5-2 minutes between sets)<br>RPE 12-13; 1-RM: 50-69%<br>• Shoulder press<br>• Lat pull-downs<br>• Hip kickbacks<br>• Hip abductions<br>• Leg extensions<br>• Triceps extensions<br>• Abdominal twists<br>• Side planks

Neuromotor Exercise Workout Examples at Light to Moderate Intensity Week 5.

Component	Time	Type	Examples
Neuromotor Exercise	20-30 minutes	Training that involves motor skills such as balance, coordination, agility	- Yoga (Hatha sitting/Vinyasa/ Nadisodhana)<br>- Tai chi (qi gong, sitting)<br>- Pilates (traditional)<br>- Balance exercise (15-30 seconds per hold, 5-6 cycles):<br>• two-legged stance<br>• semi-tandem stance<br>• reaching from a narrow stance<br>• tandem walking<br>• standing with eyes closed

Week 6

Aerobic exercise: 3-4 days* per week for 30-50 minutes* per day at moderate intensity, to total 150 minutes per week. **No more than 2 consecutive days without exercise. **Can be small doses accumulated throughout the day.*

Resistance exercise: 1-2 days* per week for ~30 minutes per session at moderate intensity. **Perform on nonconsecutive days.*

Neuromotor exercise: 2 days per week for 20-30 minutes per session at moderate intensity.

Flexibility exercise: 2 days per week for ~5 minutes per session*, stretching to the point of tightness or slight discomfort. **Can be implemented into warm-ups or cool-downs.*

Aerobic Exercise Workout Examples at Moderate Intensity Week 6.

Component	Time	Type	Examples
1) Warm-up	5 minutes	Aerobic activity or dynamic stretching	- Walking at a talking pace on a treadmill<br>- Walking to the gym<br>- Dynamic stretching (3-6 sets of 30-90 sec with 15 sec rest periods between sets):<br>• Marching in place<br>• Walking lunges<br>• Hip circles or openers<br>• Arm swings/arm circles<br>• Torso twists
2) Aerobic Exercise	30-50 minutes	Steady state moderate intensity aerobic activity	- Walking at a pace that increases your breathing rate noticeably<br>- Peddling slowly on a stationary bicycle or leisurely cycling<br>- Peddling slowly on an elliptical machine<br>- Rowing slowly on a machine<br>- Slow dancing<br>- Aqua-aerobics
3) Cool-down	5 minutes	Static flexibility OR slowly reduce the pace of aerobic exercise	- Static stretching (10-30 seconds per stretch, 2-4 repetitions of each exercise):<br>• Standing calf stretch<br>• Quadricep stretch<br>• Kneeling hip flexor stretch (iliopsoas)<br>• Seated hamstring stretch<br>• Child's pose stretch<br>• Chest stretch<br>• Cross-body shoulder stretch<br>• Neck lateral flexion stretch

Resistance Exercise Workout Examples at Moderate Intensity Week 6.

Component	Time	Type	Example exercises with machines, free weights, bodyweight, or resistance bands
Resistance Exercise (Day 1)	~30 minutes	Resistance training for all major muscle groups	2-3 sets of 8-12 repetitions (rest 1.5-2 minutes between sets)<br>RPE 12-13; 1-RM: 50-69%<br>• Chest press<br>• Seated row<br>• Leg press<br>• Hip bridges<br>• Leg curls<br>• Arm curls<br>• Abdominal flexions<br>• Planks
Resistance Exercise (Day 2)	~30 minutes	Resistance training for all major muscle groups	2-3 sets of 8-12 repetitions (rest 1.5-2 minutes between sets)<br>RPE 12-13; 1-RM: 50-69%<br>• Shoulder press<br>• Lat pull-downs<br>• Hip kickbacks<br>• Hip abductions<br>• Leg extensions<br>• Triceps extensions<br>• Abdominal twists<br>• Side planks

Neuromotor Exercise Workout Examples at Light to Moderate Intensity Week 6.

Component	Time	Type	Examples
Neuromotor Exercise	20-30 minutes	Training that involves motor skills such as balance, coordination, agility	- Yoga (Hatha sitting/Vinyasa/ Nadisodhana)<br>- Tai chi (qi gong, sitting)<br>- Pilates (traditional)<br>- Balance exercise (15-30 seconds per hold, 5-6 cycles):<br>• two-legged stance<br>• semi-tandem stance<br>• reaching from a narrow stance<br>• tandem walking<br>• standing with eyes closed

Week 7

Aerobic exercise: 3-5 days* per week for 30-50 minutes** per day at moderate intensity or 15-25 minutes per day at vigorous intensity (or a combination), to total 175 minutes per week. **No more than 2 consecutive days without exercise. **Can be small doses accumulated throughout the day.*

Resistance exercise: 2-3 days* per week for ~45 minutes per session at moderate to vigorous intensity. **Preferably 3 days per week on nonconsecutive days.*

Neuromotor exercise: 2 days per week for 20-30 minutes per session at moderate intensity.

Flexibility exercise: 2-3 days per week for ~5 minutes per session*, stretching to the point of tightness or slight discomfort. **Can be implemented into warm-ups or cool-downs.*

Aerobic Exercise Workout Examples at Moderate Intensity Week 7.

Component	Time	Type	Examples
1) Warm-up	5 minutes	Aerobic activity or dynamic stretching	- Walking at a talking pace on a treadmill<br>- Walking to the gym<br>- Dynamic stretching (3-6 sets of 30-90 sec with 15 sec rest periods between sets):<br>• Marching in place<br>• Walking lunges<br>• Hip circles or openers<br>• Arm swings/arm circles<br>• Torso twists
2) Aerobic Exercise	30-50 minutes<br><br>OR<br><br>15-25 minutes	Steady state moderate intensity aerobic activity<br><br>OR<br><br>Steady state vigorous intensity aerobic activity or high intensity interval training	- Walking at a pace that increases your breathing rate noticeably<br>- Peddling slowly on a stationary bicycle or leisurely cycling<br>- Peddling slowly on an elliptical machine<br>- Rowing slowly on a machine<br>- Slow dancing<br>- Aqua-aerobics<br><br>- Walking very briskly, jogging, or running<br>- Peddling fast on a stationary bicycle or cycling fast or uphill<br>- Peddling fast on an elliptical machine<br>- Rowing fast on a machine<br>- Stepping on the stair stepper machine<br>- Fast dancing<br>- Aerobics
3) Cool-down	5 minutes	Static flexibility OR slowly reduce the pace of aerobic exercise	- Static stretching (10-30 seconds per stretch, 2-4 repetitions of each exercise):<br>• Standing calf stretch<br>• Quadricep stretch<br>• Kneeling hip flexor stretch (iliopsoas)<br>• Seated hamstring stretch

			• Child's pose stretch<br>• Chest stretch<br>• Cross-body shoulder stretch<br>• Neck lateral flexion stretch

Resistance Exercise Workout Examples at Moderate to Vigorous Intensity Week 7.

Component	Time	Type	Example exercises with machines, free weights, bodyweight, or resistance bands
Resistance Exercise (Day 1)	~45 minutes	Resistance training for all major muscle groups	2-3 sets of 8-12 repetitions (rest 1.5-2.5 minutes between sets)<br>RPE 12-13 to 14-17; 1-RM: 50-85%<br>• Chest press<br>• Seated row<br>• Leg press<br>• Hip bridges<br>• Leg curls<br>• Arm curls<br>• Abdominal flexions<br>• Planks
Resistance Exercise (Day 2)	~45 minutes	Resistance training for all major muscle groups	2-3 sets of 8-12 repetitions (rest 1.5-2.5 minutes between sets)<br>RPE 12-13 to 14-17; 1-RM: 50-85%<br>• Shoulder press<br>• Lat pull-downs<br>• Hip kickbacks<br>• Hip abductions<br>• Leg extensions<br>• Triceps extensions<br>• Abdominal twists<br>• Side planks
Resistance Exercise (Day 3)	~45 minutes	Resistance training for all major muscle groups	2-3 sets of 8-12 repetitions (rest 1.5-2.5 minutes between sets)<br>RPE 12-13 to 14-17; 1-RM: 50-85%<br>• Pick 8-10 exercises from above to train major muscle groups

Neuromotor Exercise Workout Examples at Moderate Intensity Week 7.

Component	Time	Type	Examples
Neuromotor Exercise	20-30 minutes	Training that involves motor skills such as balance, coordination, agility	- Yoga (power/Surya Namaskar)<br>- Tai chi (qi gong standing/yang style)<br>- Pilates (POUND® with drumming)<br>- Single leg or Bosu ball exercises<br>- Balance exercises (15-30 seconds per hold, 5-6 cycles):<br>• tandem stance<br>• one-legged stance<br>• stepping over obstacles<br>• heel or toe walks<br>• walking while turning the head

Week 8

Aerobic exercise: 3-5 days* per week for 30-50 minutes** per day at moderate intensity or 15-25 minutes per day at vigorous intensity (or a combination), to total 175 minutes per week. **No more than 2 consecutive days without exercise. **Can be small doses accumulated throughout the day.*

Resistance exercise: 2-3 days* per week for ~45 minutes per session at moderate to vigorous intensity. **Preferably 3 days per week on nonconsecutive days.*

Neuromotor exercise: 2 days per week for 20-30 minutes per session at moderate intensity.

Flexibility exercise: 2-3 days per week for ~5 minutes per session*, stretching to the point of tightness or slight discomfort. **Can be implemented into warm-ups or cool-downs.*

Aerobic Exercise Workout Examples at Moderate Intensity Week 8.

Component	Time	Type	Examples
1) Warm-up	5 minutes	Aerobic activity or dynamic stretching	- Walking at a talking pace on a treadmill<br>- Walking to the gym<br>- Dynamic stretching (3-6 sets of 30-90 sec with 15 sec rest periods between sets):<br>• Marching in place<br>• Walking lunges<br>• Hip circles or openers<br>• Arm swings/arm circles<br>• Torso twists
2) Aerobic Exercise	30-50 minutes<br><br>OR<br><br>15-25 minutes	Steady state moderate intensity aerobic activity<br><br>OR<br><br>Steady state vigorous intensity aerobic activity or high intensity interval training	- Walking at a pace that increases your breathing rate noticeably<br>- Peddling slowly on a stationary bicycle or leisurely cycling<br>- Peddling slowly on an elliptical machine<br>- Rowing slowly on a machine<br>- Slow dancing<br>- Aqua-aerobics<br><br>- Walking very briskly, jogging, or running<br>- Peddling fast on a stationary bicycle or cycling fast or uphill<br>- Peddling fast on an elliptical machine<br>- Rowing fast on a machine<br>- Stepping on the stair stepper machine<br>- Fast dancing<br>- Aerobics
3) Cool-down	5 minutes	Static flexibility OR slowly reduce the pace of aerobic exercise	- Static stretching (10-30 seconds per stretch, 2-4 repetitions of each exercise):<br>• Standing calf stretch<br>• Quadricep stretch<br>• Kneeling hip flexor stretch (iliopsoas)<br>• Seated hamstring stretch

			• Child's pose stretch<br>• Chest stretch<br>• Cross-body shoulder stretch<br>• Neck lateral flexion stretch

Resistance Exercise Workout Examples at Moderate to Vigorous Intensity Week 8.

Component	Time	Type	Example exercises with machines, free weights, bodyweight, or resistance bands
Resistance Exercise (Day 1)	~45 minutes	Resistance training for all major muscle groups	2-3 sets of 8-12 repetitions (rest 1.5-2.5 minutes between sets)<br>RPE 12-13 to 14-17; 1-RM: 50-85%<br>• Chest press<br>• Seated row<br>• Leg press<br>• Hip bridges<br>• Leg curls<br>• Arm curls<br>• Abdominal flexions<br>• Planks
Resistance Exercise (Day 2)	~45 minutes	Resistance training for all major muscle groups	2-3 sets of 8-12 repetitions (rest 1.5-2.5 minutes between sets)<br>RPE 12-13 to 14-17; 1-RM: 50-85%<br>• Shoulder press<br>• Lat pull-downs<br>• Hip kickbacks<br>• Hip abductions<br>• Leg extensions<br>• Triceps extensions<br>• Abdominal twists<br>• Side planks
Resistance Exercise (Day 3)	~45 minutes	Resistance training for all major muscle groups	2-3 sets of 8-12 repetitions (rest 1.5-2.5 minutes between sets)<br>RPE 12-13 to 14-17; 1-RM: 50-85%<br>• Pick 8-10 exercises from above to train major muscle groups

Neuromotor Exercise Workout Examples at Moderate Intensity Week 8.

Component	Time	Type	Examples
Neuromotor Exercise	20-30 minutes	Training that involves motor skills such as balance, coordination, agility	- Yoga (power/Surya Namaskar)<br>- Tai chi (qi gong standing/yang style)<br>- Pilates (POUND® with drumming)<br>- Single leg or Bosu ball exercises<br>- Balance exercises (15-30 seconds per hold, 5-6 cycles):<br>• tandem stance<br>• one-legged stance<br>• stepping over obstacles<br>• heel or toe walks<br>• walking while turning the head

Week 9

Aerobic exercise: 3-5 days* per week for 30-50 minutes** per day at moderate intensity or 15-25 minutes per day at vigorous intensity (or a combination), to total 175 minutes per week. **No more than 2 consecutive days without exercise. **Can be small doses accumulated throughout the day.*

Resistance exercise: 2-3 days* per week for ~45 minutes per session at moderate to vigorous intensity. **Preferably 3 days per week on nonconsecutive days.*

Neuromotor exercise: 2 days per week for 20-30 minutes per session at moderate intensity.

Flexibility exercise: 2-3 days per week for ~5 minutes per session*, stretching to the point of tightness or slight discomfort. **Can be implemented into warm-ups or cool-downs.*

Aerobic Exercise Workout Examples at Moderate Intensity Week 9.

Component	Time	Type	Examples
1) Warm-up	5 minutes	Aerobic activity or dynamic stretching	- Walking at a talking pace on a treadmill<br>- Walking to the gym<br>- Dynamic stretching (3-6 sets of 30-90 sec with 15 sec rest periods between sets):<br>• Marching in place<br>• Walking lunges<br>• Hip circles or openers<br>• Arm swings/arm circles<br>• Torso twists
2) Aerobic Exercise	30-50 minutes<br><br>OR<br><br>15-25 minutes	Steady state moderate intensity aerobic activity<br><br>OR<br><br>Steady state vigorous intensity aerobic activity or high intensity interval training	- Walking at a pace that increases your breathing rate noticeably<br>- Peddling slowly on a stationary bicycle or leisurely cycling<br>- Peddling slowly on an elliptical machine<br>- Rowing slowly on a machine<br>- Slow dancing<br>- Aqua-aerobics<br><br>- Walking very briskly, jogging, or running<br>- Peddling fast on a stationary bicycle or cycling fast or uphill<br>- Peddling fast on an elliptical machine<br>- Rowing fast on a machine<br>- Stepping on the stair stepper machine<br>- Fast dancing<br>- Aerobics
3) Cool-down	5 minutes	Static flexibility OR slowly reduce the pace of aerobic exercise	- Static stretching (10-30 seconds per stretch, 2-4 repetitions of each exercise):<br>• Standing calf stretch<br>• Quadricep stretch<br>• Kneeling hip flexor stretch (iliopsoas)<br>• Seated hamstring stretch

			• Child's pose stretch<br>• Chest stretch<br>• Cross-body shoulder stretch<br>• Neck lateral flexion stretch

Resistance Exercise Workout Examples at Moderate to Vigorous Intensity Week 9.

Component	Time	Type	Example exercises with machines, free weights, bodyweight, or resistance bands
Resistance Exercise (Day 1)	~45 minutes	Resistance training for all major muscle groups	2-3 sets of 8-12 repetitions (rest 1.5-2.5 minutes between sets)<br>RPE 12-13 to 14-17; 1-RM: 50-85%<br>• Chest press<br>• Seated row<br>• Leg press<br>• Hip bridges<br>• Leg curls<br>• Arm curls<br>• Abdominal flexions<br>• Planks
Resistance Exercise (Day 2)	~45 minutes	Resistance training for all major muscle groups	2-3 sets of 8-12 repetitions (rest 1.5-2.5 minutes between sets)<br>RPE 12-13 to 14-17; 1-RM: 50-85%<br>• Shoulder press<br>• Lat pull-downs<br>• Hip kickbacks<br>• Hip abductions<br>• Leg extensions<br>• Triceps extensions<br>• Abdominal twists<br>• Side planks
Resistance Exercise (Day 3)	~45 minutes	Resistance training for all major muscle groups	2-3 sets of 8-12 repetitions (rest 1.5-2.5 minutes between sets)<br>RPE 12-13 to 14-17; 1-RM: 50-85%<br>• Pick 8-10 exercises from above to train major muscle groups

Neuromotor Exercise Workout Examples at Moderate Intensity Week 9.

Component	Time	Type	Examples
Neuromotor Exercise	20-30 minutes	Training that involves motor skills such as balance, coordination, agility	- Yoga (power/Surya Namaskar)<br>- Tai chi (qi gong standing/yang style)<br>- Pilates (POUND® with drumming)<br>- Single leg or Bosu ball exercises<br>- Balance exercises (15-30 seconds per hold, 5-6 cycles):<br>• tandem stance<br>• one-legged stance<br>• stepping over obstacles<br>• heel or toe walks<br>• walking while turning the head

Week 10

Aerobic exercise: 3-7 days* per week for 30-60 minutes** per day at moderate intensity or 15-30 minutes per day at vigorous intensity (or a combination), to total 200-300 minutes per week. **No more than 2 consecutive days without exercise. **Can be small doses accumulated throughout the day.*

Resistance exercise: 2-3 days* per week for ~45 minutes per session at moderate to vigorous intensity. **Preferably 3 days per week on nonconsecutive days.*

Neuromotor exercise: 2 days per week for 20-30 minutes per session at moderate intensity.

Flexibility exercise: 2-3 days per week for ~5 minutes per session*, stretching to the point of tightness or slight discomfort. **Can be implemented into warm-ups or cool-downs.*

Aerobic Exercise Workout Examples at Moderate Intensity Week 10.

Component	Time	Type	Examples
1) Warm-up	5 minutes	Aerobic activity or dynamic stretching	- Walking at a talking pace on a treadmill<br>- Walking to the gym<br>- Dynamic stretching (3-6 sets of 30-90 sec with 15 sec rest periods between sets):<br>• Marching in place<br>• Walking lunges<br>• Hip circles or openers<br>• Arm swings/arm circles<br>• Torso twists
2) Aerobic Exercise	30-60 minutes<br><br>OR<br><br>15-30 minutes	Steady state moderate intensity aerobic activity<br><br>OR<br><br>Steady state vigorous intensity aerobic activity or high intensity interval training	- Walking at a pace that increases your breathing rate noticeably<br>- Peddling slowly on a stationary bicycle or leisurely cycling<br>- Peddling slowly on an elliptical machine<br>- Rowing slowly on a machine<br>- Slow dancing<br>- Aqua-aerobics<br><br>- Walking very briskly, jogging, or running<br>- Peddling fast on a stationary bicycle or cycling fast or uphill<br>- Peddling fast on an elliptical machine<br>- Rowing fast on a machine<br>- Stepping on the stair stepper machine<br>- Fast dancing<br>- Aerobics
3) Cool-down	5 minutes	Static flexibility OR slowly reduce the pace of aerobic exercise	- Static stretching (10-30 seconds per stretch, 2-4 repetitions of each exercise):<br>• Standing calf stretch<br>• Quadricep stretch<br>• Kneeling hip flexor stretch (iliopsoas)<br>• Seated hamstring stretch

			• Child's pose stretch<br>• Chest stretch<br>• Cross-body shoulder stretch<br>• Neck lateral flexion stretch

Resistance Exercise Workout Examples at Moderate to Vigorous Intensity Week 10.

Component	Time	Type	Example exercises with machines, free weights, bodyweight, or resistance bands
Resistance Exercise (Day 1)	~45 minutes	Resistance training for all major muscle groups	2-3 sets of 8-12 repetitions (rest 1.5-2.5 minutes between sets)<br>RPE 12-13 to 14-17; 1-RM: 50-85%<br>• Chest press<br>• Seated row<br>• Leg press<br>• Hip bridges<br>• Leg curls<br>• Arm curls<br>• Abdominal flexions<br>• Planks
Resistance Exercise (Day 2)	~45 minutes	Resistance training for all major muscle groups	2-3 sets of 8-12 repetitions (rest 1.5-2.5 minutes between sets)<br>RPE 12-13 to 14-17; 1-RM: 50-85%<br>• Shoulder press<br>• Lat pull-downs<br>• Hip kickbacks<br>• Hip abductions<br>• Leg extensions<br>• Triceps extensions<br>• Abdominal twists<br>• Side planks
Resistance Exercise (Day 3)	~45 minutes	Resistance training for all major muscle groups	2-3 sets of 8-12 repetitions (rest 1.5-2.5 minutes between sets)<br>RPE 12-13 to 14-17; 1-RM: 50-85%<br>• Pick 8-10 exercises from above to train major muscle groups

Neuromotor Exercise Workout Examples at Moderate Intensity Week 10.

Component	Time	Type	Examples
Neuromotor Exercise	20-30 minutes	Training that involves motor skills such as balance, coordination, agility	- Yoga (power/Surya Namaskar)<br>- Tai chi (qi gong standing/yang style)<br>- Pilates (POUND® with drumming)<br>- Single leg or Bosu ball exercises<br>- Balance exercises (15-30 seconds per hold, 5-6 cycles):<br>• tandem stance<br>• one-legged stance<br>• stepping over obstacles<br>• heel or toe walks<br>• walking while turning the head

Week 11

Aerobic exercise: 3-7 days* per week for 30-60 minutes** per day at moderate intensity or 15-30 minutes per day at vigorous intensity (or a combination), to total 200-300 minutes per week. **No more than 2 consecutive days without exercise. **Can be small doses accumulated throughout the day.*

Resistance exercise: 2-3 days* per week for ~45 minutes per session at moderate to vigorous intensity. **Preferably 3 days per week on nonconsecutive days.*

Neuromotor exercise: 2 days per week for 20-30 minutes per session at moderate intensity.

Flexibility exercise: 2-3 days per week for ~5 minutes per session*, stretching to the point of tightness or slight discomfort. **Can be implemented into warm-ups or cool-downs.*

Aerobic Exercise Workout Examples at Moderate Intensity Week 11.

Component	Time	Type	Examples
1) Warm-up	5 minutes	Aerobic activity or dynamic stretching	- Walking at a talking pace on a treadmill<br>- Walking to the gym<br>- Dynamic stretching (3-6 sets of 30-90 sec with 15 sec rest periods between sets):<br>• Marching in place<br>• Walking lunges<br>• Hip circles or openers<br>• Arm swings/arm circles<br>• Torso twists
2) Aerobic Exercise	30-60 minutes<br>OR<br>15-30 minutes	Steady state moderate intensity aerobic activity<br>OR<br>Steady state vigorous intensity aerobic activity or high intensity interval training	- Walking at a pace that increases your breathing rate noticeably<br>- Peddling slowly on a stationary bicycle or leisurely cycling<br>- Peddling slowly on an elliptical machine<br>- Rowing slowly on a machine<br>- Slow dancing<br>- Aqua-aerobics<br><br>- Walking very briskly, jogging, or running<br>- Peddling fast on a stationary bicycle or cycling fast or uphill<br>- Peddling fast on an elliptical machine<br>- Rowing fast on a machine<br>- Stepping on the stair stepper machine<br>- Fast dancing<br>- Aerobics
3) Cool-down	5 minutes	Static flexibility OR slowly reduce the pace of aerobic exercise	- Static stretching (10-30 seconds per stretch, 2-4 repetitions of each exercise):<br>• Standing calf stretch<br>• Quadricep stretch<br>• Kneeling hip flexor stretch (iliopsoas)<br>• Seated hamstring stretch

			• Child's pose stretch<br>• Chest stretch<br>• Cross-body shoulder stretch<br>• Neck lateral flexion stretch

Resistance Exercise Workout Examples at Moderate to Vigorous Intensity Week 11.

Component	Time	Type	Example exercises with machines, free weights, bodyweight, or resistance bands
Resistance Exercise (Day 1)	~45 minutes	Resistance training for all major muscle groups	2-3 sets of 8-12 repetitions (rest 1.5-2.5 minutes between sets)<br>RPE 12-13 to 14-17; 1-RM: 50-85%<br>• Chest press<br>• Seated row<br>• Leg press<br>• Hip bridges<br>• Leg curls<br>• Arm curls<br>• Abdominal flexions<br>• Planks
Resistance Exercise (Day 2)	~45 minutes	Resistance training for all major muscle groups	2-3 sets of 8-12 repetitions (rest 1.5-2.5 minutes between sets)<br>RPE 12-13 to 14-17; 1-RM: 50-85%<br>• Shoulder press<br>• Lat pull-downs<br>• Hip kickbacks<br>• Hip abductions<br>• Leg extensions<br>• Triceps extensions<br>• Abdominal twists<br>• Side planks
Resistance Exercise (Day 3)	~45 minutes	Resistance training for all major muscle groups	2-3 sets of 8-12 repetitions (rest 1.5-2.5 minutes between sets)<br>RPE 12-13 to 14-17; 1-RM: 50-85%<br>• Pick 8-10 exercises from above to train major muscle groups

Neuromotor Exercise Workout Examples at Moderate Intensity Week 11.

Component	Time	Type	Examples
Neuromotor Exercise	20-30 minutes	Training that involves motor skills such as balance, coordination, agility	- Yoga (power/Surya Namaskar)<br>- Tai chi (qi gong standing/yang style)<br>- Pilates (POUND® with drumming)<br>- Single leg or Bosu ball exercises<br>- Balance exercises (15-30 seconds per hold, 5-6 cycles):<br>• tandem stance<br>• one-legged stance<br>• stepping over obstacles<br>• heel or toe walks<br>• walking while turning the head

Week 12

Aerobic exercise: 3-7 days* per week for 30-60 minutes** per day at moderate intensity or 15-30 minutes per day at vigorous intensity (or a combination), to total 200-300 minutes per week. **No more than 2 consecutive days without exercise. **Can be small doses accumulated throughout the day.*

Resistance exercise: 2-3 days* per week for ~45 minutes per session at moderate to vigorous intensity. **Preferably 3 days per week on nonconsecutive days.*

Neuromotor exercise: 2 days per week for 20-30 minutes per session at moderate intensity.

Flexibility exercise: 2-3 days per week for ~5 minutes per session*, stretching to the point of tightness or slight discomfort. **Can be implemented into warm-ups or cool-downs.*

Aerobic Exercise Workout Examples at Moderate Intensity Week 12.

Component	Time	Type	Examples
1) Warm-up	5 minutes	Aerobic activity or dynamic stretching	- Walking at a talking pace on a treadmill<br>- Walking to the gym<br>- Dynamic stretching (3-6 sets of 30-90 sec with 15 sec rest periods between sets):<br>• Marching in place<br>• Walking lunges<br>• Hip circles or openers<br>• Arm swings/arm circles<br>• Torso twists
2) Aerobic Exercise	30-60 minutes<br><br>OR<br><br>15-30 minutes	Steady state moderate intensity aerobic activity<br><br>OR<br><br>Steady state vigorous intensity aerobic activity or high intensity interval training	- Walking at a pace that increases your breathing rate noticeably<br>- Peddling slowly on a stationary bicycle or leisurely cycling<br>- Peddling slowly on an elliptical machine<br>- Rowing slowly on a machine<br>- Slow dancing<br>- Aqua-aerobics<br><br>- Walking very briskly, jogging, or running<br>- Peddling fast on a stationary bicycle or cycling fast or uphill<br>- Peddling fast on an elliptical machine<br>- Rowing fast on a machine<br>- Stepping on the stair stepper machine<br>- Fast dancing<br>- Aerobics
3) Cool-down	5 minutes	Static flexibility OR slowly reduce the pace of aerobic exercise	- Static stretching (10-30 seconds per stretch, 2-4 repetitions of each exercise):<br>• Standing calf stretch<br>• Quadricep stretch<br>• Kneeling hip flexor stretch (iliopsoas)<br>• Seated hamstring stretch

			• Child's pose stretch<br>• Chest stretch<br>• Cross-body shoulder stretch<br>• Neck lateral flexion stretch

Resistance Exercise Workout Examples at Moderate to Vigorous Intensity Week 12.

Component	Time	Type	Example exercises with machines, free weights, bodyweight, or resistance bands
Resistance Exercise (Day 1)	~45 minutes	Resistance training for all major muscle groups	2-3 sets of 8-12 repetitions (rest 1.5-2.5 minutes between sets)<br>RPE 12-13 to 14-17; 1-RM: 50-85%<br>• Chest press<br>• Seated row<br>• Leg press<br>• Hip bridges<br>• Leg curls<br>• Arm curls<br>• Abdominal flexions<br>• Planks
Resistance Exercise (Day 2)	~45 minutes	Resistance training for all major muscle groups	2-3 sets of 8-12 repetitions (rest 1.5-2.5 minutes between sets)<br>RPE 12-13 to 14-17; 1-RM: 50-85%<br>• Shoulder press<br>• Lat pull-downs<br>• Hip kickbacks<br>• Hip abductions<br>• Leg extensions<br>• Triceps extensions<br>• Abdominal twists<br>• Side planks
Resistance Exercise (Day 3)	~45 minutes	Resistance training for all major muscle groups	2-3 sets of 8-12 repetitions (rest 1.5-2.5 minutes between sets)<br>RPE 12-13 to 14-17; 1-RM: 50-85%<br>• Pick 8-10 exercises from above to train major muscle groups

Neuromotor Exercise Workout Examples at Moderate Intensity Week 12.

Component	Time	Type	Examples
Neuromotor Exercise	20-30 minutes	Training that involves motor skills such as balance, coordination, agility	- Yoga (power/Surya Namaskar)<br>- Tai chi (qi gong standing/yang style)<br>- Pilates (POUND® with drumming)<br>- Single leg or Bosu ball exercises<br>- Balance exercises (15-30 seconds per hold, 5-6 cycles):<br>• tandem stance<br>• one-legged stance<br>• stepping over obstacles<br>• heel or toe walks<br>• walking while turning the head
